# New world goat populations are a genetically diverse reservoir for future use

**DOI:** 10.1038/s41598-019-38812-3

**Published:** 2019-02-06

**Authors:** Tiago do Prado Paim, Danielle Assis Faria, El Hamidi Hay, Concepta McManus, Maria Rosa Lanari, Laura Chaverri Esquivel, María Isabel Cascante, Esteban Jimenez Alfaro, Argerie Mendez, Olivardo Faco, Kleibe de Moraes Silva, Carlos Alberto Mezzadra, Arthur Mariante, Samuel Rezende Paiva, Harvey D. Blackburn

**Affiliations:** 1 Universidade de Brasília, Campus Darcy Ribeiro, Asa Norte, Brasília, Distrito Federal 70910-900 Brazil; 2 Instituto Federal de Educação, Ciência e Tecnologia Goiano, Fazenda Escola Campus Iporá GO-060 km 222, Iporá, Goias 76200-000 Brazil; 3 USDA Agricultural Research Service, Fort Keogh Livestock and Range Research Laboratory, 243 Fort Keogh Rd., Miles City, MT 59301 USA; 4 Instituto Nacional de Tecnologia Agropecuaria (INTA), 4450 Modesta Victoria, San Carlos de Bariloche, Río Negro, 8400 Argentina; 5 Universidad Nacional de Costa Rica, Avenida 1 Calle 9, Heredia, Heredia Province 86–3000 Costa Rica; 6 Instituto de Innovación y Transferencia en Tecnologia Agropecuaria (INTA), San José, 382–1007 Centro Colón Costa Rica; 7 Embrapa Caprinos e Ovinos, Fazenda Três Lagoas - Estrada Sobral/Groaíras Km 4, Sobral, Ceará P.O. Box: 71, 62010-970 Brazil; 8 Instituto Nacional de Tecnologia Agropecuaria (INTA), Ruta 226 km 73,5, Balcarce, Buenos Aires 7620 Argentina; 9 Embrapa Recursos Genéticos e Biotecnologia, Parque Estação Biológica, PqEB, Av. W5 Norte (final), Brasília, Distrito Federal P.O. Box: 02372, 70770-917 Brazil; 10 National Center for Genetic Resources Preservation, USDA, 1111 South Mason Street, Fort Collins, CO 80521 USA

## Abstract

Western hemisphere goats have European, African and Central Asian origins, and some local or rare breeds are reported to be adapted to their environments and economically important. By-in-large these genetic resources have not been quantified. Using 50 K SNP genotypes of 244 animals from 12 goat populations in United States, Costa Rica, Brazil and Argentina, we evaluated the genetic diversity, population structure and selective sweeps documenting goat migration to the “New World”. Our findings suggest the concept of breed, particularly among “locally adapted” breeds, is not a meaningful way to characterize goat populations. The USA Spanish goats were found to be an important genetic reservoir, sharing genomic composition with the wild ancestor and with specialized breeds (e.g. Angora, Lamancha and Saanen). Results suggest goats in the Americas have substantial genetic diversity to use in selection and promote environmental adaptation or product driven specialization. These findings highlight the importance of maintaining goat conservation programs and suggest an awaiting reservoir of genetic diversity for breeding and research while simultaneously discarding concerns about breed designations.

## Introduction

Unlike other livestock species, goats are unique in terms of their function and environments where they are utilized. Their body size, levels of production, dietary preferences, and low cost of investment make them a pliable species for livestock producers to use^1,2^. Globally, goats tend to be raised in low input production systems and generally lack high levels of artificial selection, suggesting their genetic composition may be less structured than other species^3^.

Goat domestication occurred in the Fertile Cresent^4^ from 9,900 to 10,500 YBP. The Bezoar ibex (*Capra aegagrus*) is thought to be the only living wild progenitor of the goat^5^. Upon domestication, goats accompanied human migration and trade, thereby developing subpopulations and breeds differentiated by various selection factors and genetic drift^6^.

During the colonization of the western hemisphere, settlers brought goats potentially from the Iberian Peninsula and west Africa^7^. These populations have become well adapted to low input agricultural environments typically found in northeastern Brazil, west Texas, and southern Argentina (Patagonia)^8,9^, creating locally adapted breeds. Further, multiple waves of importation to the western hemisphere have occurred and included product-specialized breeds, such as dairy (e.g., Saanen), fiber (Angora) and meat (Boer). However, western hemisphere breeding lags behind other livestock species, in part due to their low economic return^10^.

In general, local goat breeds may be largely panmictic, due to multiple importation waves, unsupervised crossbreeding and the lack of strong artificial selection. In this work, we used genotypic data (50 K SNP) from 12 goat breeds found in the Americas, augmented by genotypes from South Africa, Iran, Morocco and Bezoar ibex (Table 1) to: characterize western hemisphere goat diversity, understand genetic structure, and identify genomic regions under selection in these animals.

**Table 1:** Description of the data with 17 goat populations used in the analyses and the grouping realized for Fst and hapFLK analyses.

Abbreviation	Breed	Country of collection	n^a^	Ho^b^	F_IS_^c^	Groups^d^	16 populations^e^	12 populations^e^	Angora^e^	Argentinean and Spanish^e^
Angora_AR	Angora	Argentina	23	0.400	0.073	Fiber	x		x	
Angora_SA	Angora	South Africa	43	0.333	0.227	Fiber	x		x	
Angora_USA	Angora	United States	29	0.370	0.143	Fiber	x		x	
Boer_USA	Boer	United States	17	0.360	0.165	Meat	x	x		
C. Formosena_AR	Criolla Formosena	Argentina	13	0.378	0.124	Argentinean	x	x		x
C. Llanos_AR	Criollo de los Llanos	Argentina	13	0.391	0.093	Argentinean	x	x		x
C. Neuquino_AR	Criollo Neuquino	Argentina	17	0.410	0.050	Argentinean	x	x		x
C. Pampeana_AR	Colorada Pampeana	Argentina	11	0.400	0.072	Argentinean	x	x		x
C. Riojano_AR	Criollo Riojano	Argentina	6	0.389	0.099	Argentinean	x	x		x
Caninde_BR	Caninde	Brazil	19	0.329	0.236	Brazilian	x	x		
Moxoto_BR	Moxoto	Brazil	18	0.337	0.218	Brazilian	x	x		
LaMancha	LaMancha	United States	11	0.382	0.114	Milk	x	x		
Saanen_CR	Saanen	Costa Rica	28	0.413	0.044	Milk	x	x		
Spanish	Spanish	United States	19	0.427	0.011	Spanish	x	x		x
Morocco	not defined	Morocco	30	0.382	0.114		x	x		
Iran	not defined	Iran	9	0.377	0.125		x		x	
C_aegagrus	Bezoar ibex - *Capra aegagrus*	Iran	7	0.275	0.362	used as root				

^a^Number of samples after all filtering process applied (Sample call rate > 0.90 and genomic relationship <0.25). ^b^Ho: Heterozygosity observed. The expected heterozygosity was close to 0.431 for all breeds. ^c^F_IS_: inbreeding coefficient. ^d^Identification of groups used in Fst per marker and hapFLK analyses. ^e^Identification of each hapFLK run, populations marked were included in that comparison.

## Results

### Genetic diversity and admixture

**Figure 1:**
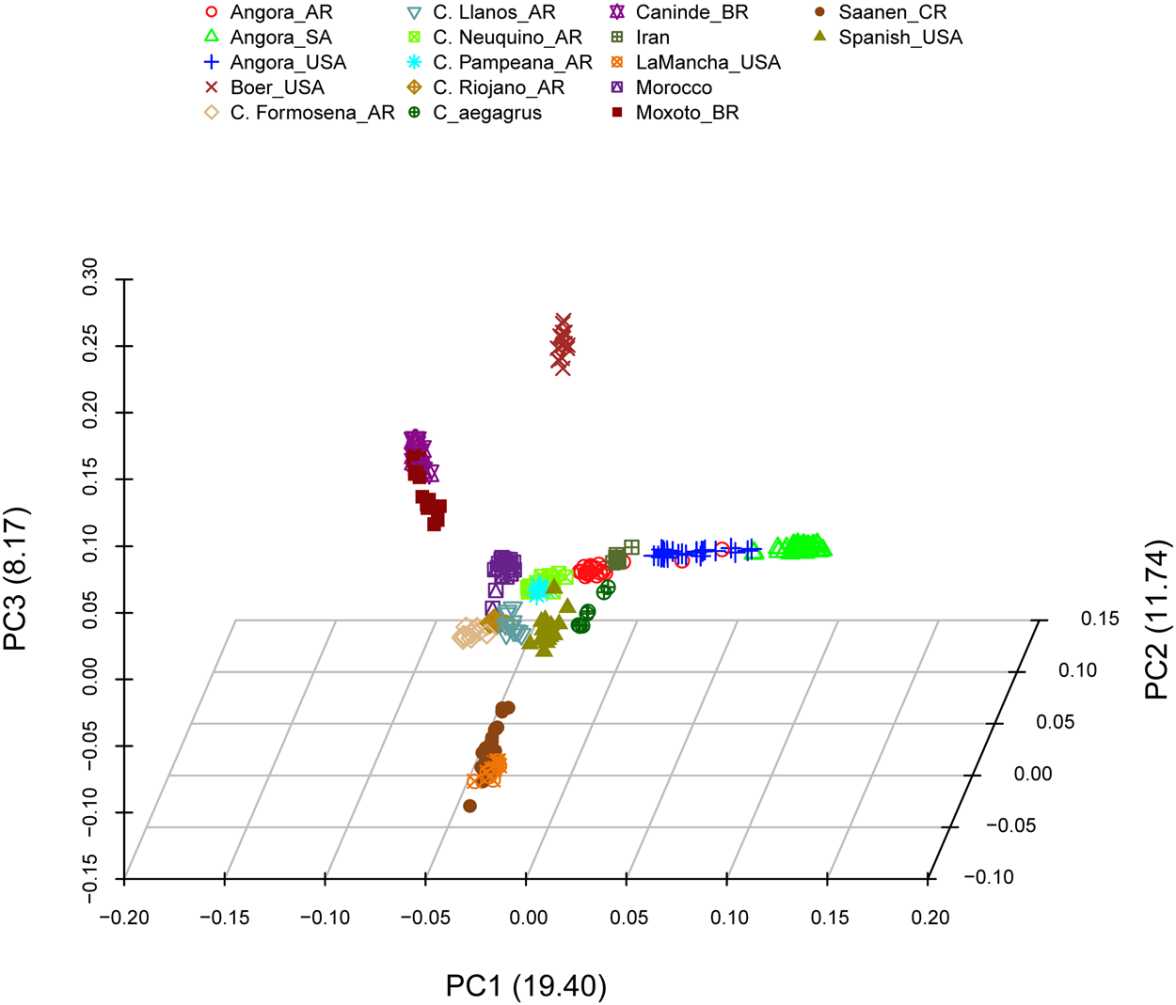
First three principal components using 17 goat populations. Values between parentheses in each axis are the eigenvalues of each component.

Biological function of the tested populations appeared to be responsible for the observed differences in the principal components analysis (PCA – Fig. 1 and Supplementary Fig. S1). The eigenvalues (Supplementary Fig. S2) showed five as a reasonable number of components to be evaluated (explain 76.8% of the variation). Five distinct groups were identified: meat (Boer), Brazilian (Moxoto and Caninde), dairy (Saanen and LaMancha), fiber (Angora) and the remaining populations in a neutral clustering position. Angora populations showed a dispersed pattern where; the admixed Argentinean (AR) population was placed closer to the graph’s origin, while South African (SA) Angora with higher inbreeding levels were the most distant from the origin, and USA Angora were in an intermediate position.

Bezoar ibex had the highest inbreeding coefficient (0.36). Brazilian breeds had the highest number of monomorphic SNPs and inbreeding coefficients (12%, 0.24 and 6%, 0.22 for Caninde and Moxoto, respectively) from the New World samples. Compared to Angora_USA, Angora_SA had a higher inbreeding coefficient (0.14 vs 0.23, respectively). The Spanish breed had the lowest inbreeding coefficient (0.01), while Saanen_CR and C. Neuquino_AR also had low inbreeding levels (Table 1).

**Figure 2:**
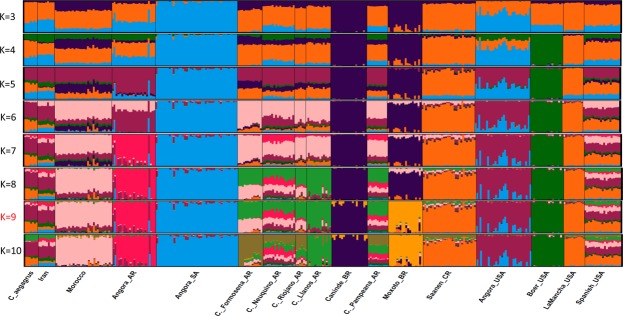
Plot of model-based clustering (ADMIXTURE) results from K equal 3 to 10 using 17 goat populations.

Cross validation error of ADMIXTURE^11^ indicated K = 9 as the optimal number of populations (Supplementary Fig. S3). Seven populations were substantially admixed at K = 9 (Fig. 2). High proportions of the Angora_USA cluster (81.6% of assignment to this cluster) were found in *C*. *aegagrus* (48.1%), Iran (42.2%), Spanish_USA (26.2%), Morocco (12.1%) and all Argentinean breeds (11.6%). The dairy breed cluster (Lamancha_USA, 92.3%; and Saanen_CR, 80.5%) was observed in Spanish_USA (31.0%) and *C*. *aegagrus* (30.4%). The cluster that represented 80.2% of genomic composition of C. Llanos_AR was observed in Spanish_USA (11.0%) and other nondescript Argentinean breeds (Table 1). The Moroccan goat cluster (77.4%) was observed in some Argentinean breeds (Formosena – 39.7%, Riojano – 26.6%, Neuquino – 21.3% and Pampeana – 15.3%), as well as in Spanish_USA (16.2%) and Iran (12.3%). As these result suggest, Spanish_USA was found to be highly admixed and when K was increased (10 to 16) no specific cluster for Spanish_USA was identified (Supplementary Fig. S4). Local breeds from Argentina were grouped together or shared the same clusters in the PCA and ADMIXTURE results, with the exception of C. Llanos (Supplementary Fig. S4).

**Figure 3:**
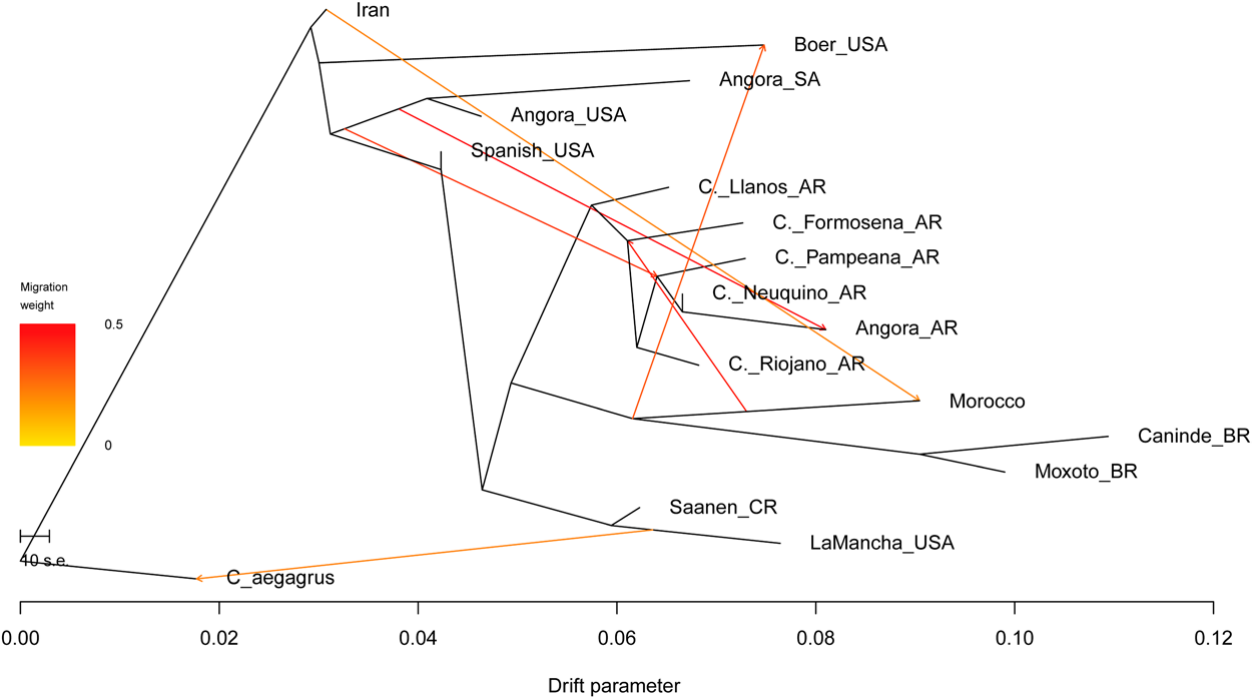
Population tree with 17 goat populations from TreeMix software using *Capra aegagrus* as root and showing six migration events.

Population trees were constructed using Treemix software^12^ by running simulations of 0 to 20 migration events (applying three replications per migration event). Likelihood estimates indicated 6 to 9 migration events best fit the model (Supplementary Figs S5 and S6). According to the residual values of the model for six migration events (Supplementary Fig. S7), the relationship between only few pairs of populations (C. Formosena with Morocco; Lamancha with Spanish breed; Saanen_CR with C. Formosena; C. Formosena and C. Riojano with Brazilian breeds) are not well explained. Therefore, the tree with six migrations was chosen as the reference for analysis of the ancestral relationships of these goat populations (Fig. 3).

### Selection signatures and gene annotation

Selection sweeps were conducted using the main groups (Boer/Meat, Dairy, Brazilian, Argentinean, Spanish and Angora) identified by the genetic structure analyses (PCA and ADMIXTURE). Pairwise Fst suggested high differentiation of Brazilian breeds, Angora_SA and Boer_USA (Supplementary Fig. S8). Spanish, Morocco and C. Neuquino_AR had low levels of genetic differentiation in relation to all the other populations. Fst analyses per marker were performed with various paired comparisons as shown in Supplementary Table S1. These comparisons showed various selection sweeps (Supplementary Tables S2 and S3).

**Figure 4:**
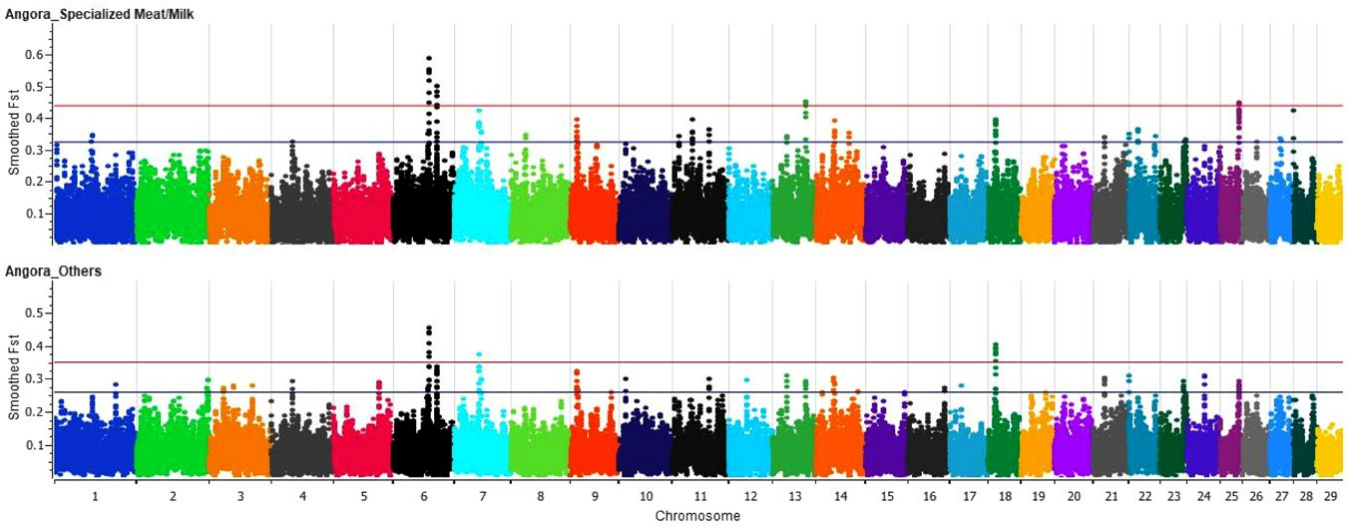
Smoothed Fst per SNP for comparison between Angora and Meat (Boer) and Milk (Saanen and LaMancha) specialized breeds and between Angora and all others goat breeds in the analyses. Red line: significant threshold of three standard deviations above the mean. Blue line: threshold of two standard deviations above the mean.

Using only the South Africa and USA Angora populations, significant loci (above three standard deviations) on chromosomes 6, 7, 13, 18 and 25 (Fig. 4) were identified. Significant regions also were seen for dairy, meat, Argentinean and Spanish populations (Supplementary Figs S9–S12). The Brazilian breeds did not show any significant regions (Supplementary Fig. S13). An additional Fst comparison among specialized breed groups (Fiber, Meat and Milk) versus local breeds (Argentinean, Spanish and Brazilian) did not show any significant regions (Supplementary Fig. S14).

Selection signatures were also identified using a haplotype based approach (hapFLK)^13^. HapFLK analyses used the same breed groupings except that Bezoar ibex was used as the root. Five regions with reduced haplotype diversity were identified and considered selection signatures for these groups (Table 2).

**Table 2:** Summary of significant selected regions identified using hapFLK analyses per group.

Chr	Starts (Mb)	End (Mb)	Size (Mb)	SNP^a^	Population selected^b^	N genes^c^	Causal variants^d^	Genes associated with causal variants^e^	Overlaps^f^
2	119.2	119.8	0.64	101	Meat; Brazil	2	5	CWC22, ZNF385B	Meat^50^
3	24.1	37.8	13.73	351	Meat	124	5	LOC106501971, MRPL37, OSBPL9	Barki (Hot and arid)^29^; Meat(Boer)^50^
7	60.6	62.7	2.12	132	Meat; Brazil	23	7	SPOCK1, MYOT, TRPC7, LOC106502333	
10	34.2	35.7	1.48	110	Brazil	14	8	PSMA3, CCDC198, C10H14orf37	Milk^50^
23	45.1	48.8	4.18	125	Angora	15	5	DST, KHDRBS2	Wild^5^

Chr: Chromosome; Start, End: beginning and end of the genome window analyzed considering a window of 2 Mb for each side from the last significant SNP. ^a^Number of SNP in the region; ^b^Population that have received selection pressure in the region based on the tree and cluster plot analyses; ^c^Number of genes founded in each region; ^d^Number of SNPs in the region identified as causal variants using CAVIAR software; ^e^Genes associated with at least one SNP causal variant (distant at maximum 500 kb of the SNP). ^f^Overlaps with previous studies (showed by numbers in reference section) of selection signature in goats (showed the name of the population or trait).

**Figure 5:**
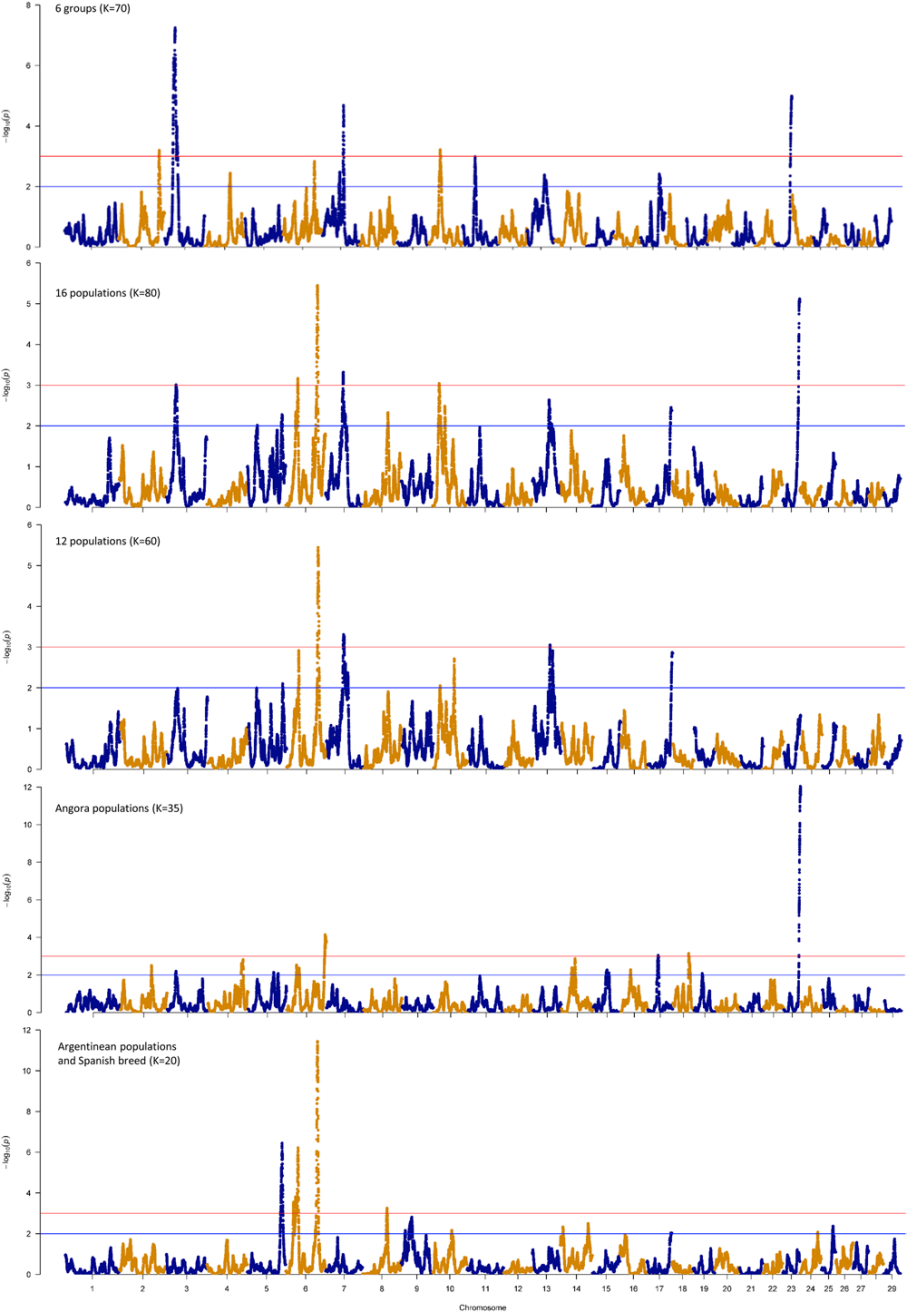
Genome scan for selection in five different scenarios of 16 populations of goat using a haplotype-based (hapFLK) test. 6 groups: Boer/Meat, Argentinean, Brazilian, Milk, Spanish and Angora breeds. 16 populations: Angora_AR, Angora_SA, Angora_USA, Iran, Boer_USA, Argentinean populations, Caninde_BR, Moxoto_BR, LaMancha_USA, Morocco, Saanen_CR, Spanish_USA. 12 populations: 16 populations minus Angora and Iran populations. Angora populations: Angora animals from South Africa, United States and Argentina. Argentinean populations: C. Formosena_AR, C. Llanos_ AR, C. Neuquino_AR, C. Pampeana_AR, C. Riojano_AR.

HapFLK’s detection power significantly decreases when populations are too genetically distant from each other^13^. To improve the haplotype estimation process, five different hapFLK runs were performed (Fig. 5) grouping the samples by the populations in Table 1. Thereby seeking to overcome bias generated by genetic distance between the populations. By taking this approach, we observed sixteen regions with reduced haplotype diversity (Supplementary Table S4).

**Figure 6:**
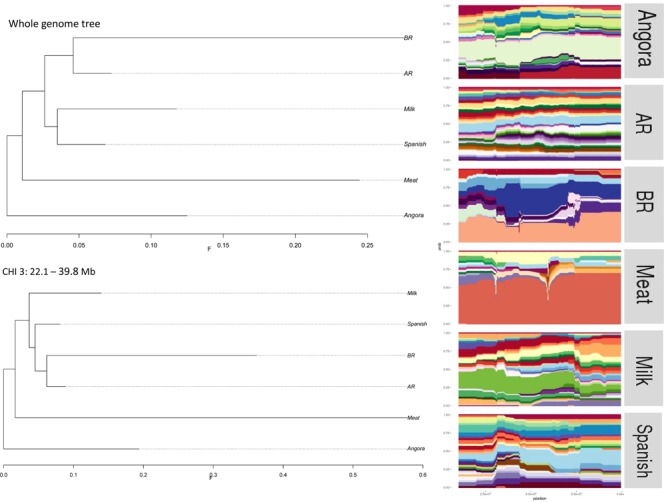
Group trees (at left) generated using all available SNPs and only 351 SNPs surrounding the hapFLK peak in chromosome 3 analyzing the six groups. Haplotype clusters frequencies (at right) in the region of chromosome 3 for each group used in the test. AR: Argentinean breeds; BR: Brazilian breeds. This peak are used as example here, the others significant regions are showed in supplementary files.

**Figure 7:**
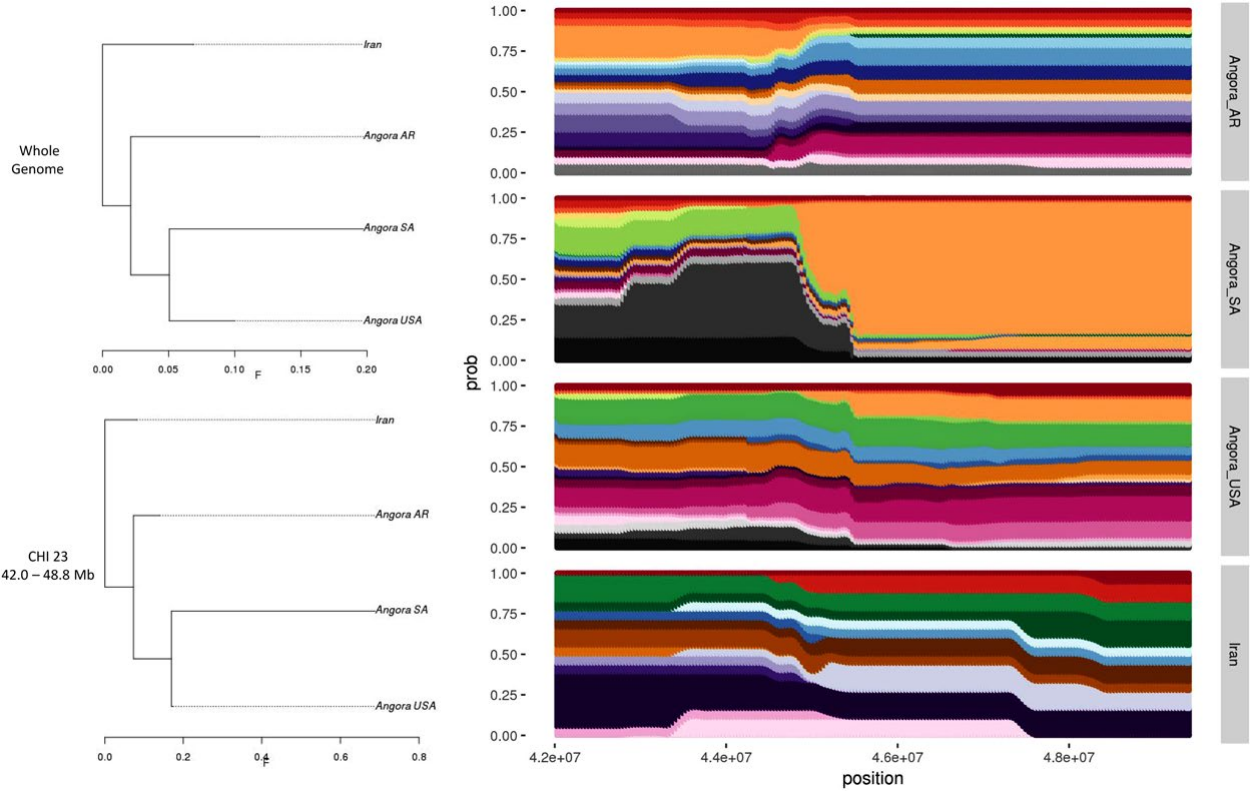
Population trees (at left) generated using all available SNPs and only 150 SNPs surrounding the hapFLK peak in chromosome 23 analyzing only the Angora populations. Haplotype clusters frequencies (at right) in the region of chromosome 23 for each population used in the test.

For each significant region detected in hapFLK analyses, population tree and haplotype clusters were plotted to identify the populations or group that had selection pressure in each region. A region on chromosome 3 (Fig. 6), for example, indicated a selection signal in Boer (Meat group). Three hapFLK runs (Groups, 16 populations and Angora, as described in Table 1) suggested a strong selection sweep signal in Angora_SA on chromosome 23 (Fig. 7).

A region on chromosome 6 observed in two hapFLK analyses (12 and 16 populations) highlighted a selection sweep signal in Caninde_BR and Moxoto_BR (Supplementary Figs S19, S23 and S28). C. Neuquino_AR also displays a soft selection sweep in this region, which was later confirmed when running hapFLK with only Spanish and Argentinean local breeds (Supplementary Fig. S36).

All other selected regions were evaluated and the selected populations in each region were determined (Supplementary Figs S15 to S37). The spectral decomposition of the signal in each region^13^ was also evaluated (Supplementary Fig. S38). Significant regions and gene annotation for each selected region are presented in Table 2 and Supplementary Tables S2 to S4. Several genes observed in the selected regions have been associated with various traits in other livestock species (pig, cattle or sheep) (Supplementary Table S5). Gene ontology of the significant regions for group comparison are shown in supplementary information (Supplementary Tables S7 and S8).

## Discussion

A plausible path from the center of domestication and migration to the western hemisphere^14^ is presented in Fig. 3. Post-domestication dispersal of goats to the west was characterized by migrations routes through Europe (Danubian and Mediterranean corridors), and North Africa (by Sinai Penisula or Mediterranena sea) and later a south migration via eastern Africa^14^. The early partition of Boer and Angora supported the southern distribution via eastern Africa for Boer formation in South Africa and the Angora development in Turkey. The remaining breeds formed a branch of European-ancestry. The Spanish breed was placed closely to the beginning of this branch and had a drift parameter indicating little change from the populations found near the center of domestication, suggesting the genetic diversity conserved in this population. At a later point, Treemix suggested this branch segregated into dairy and South American breeds.

South American breeds diverged in two branches (Argentinean and Brazilian), suggesting the progenitors were from both Spanish and Portuguese populations which were pivotal sources of genetics exported to the western hemisphere^7^. The close association of the Moroccan and Brazilian populations and migration events (Fig. 3) are consistent with information concerning trade flows among the Iberian Peninsula, North Africa, and the Canary and Cape Verde Islands^15,16^.

A weak genetic structure was observed (Fig. 1) for Spanish, Argentinean local breeds, Moroccan and Iranian populations which were closely placed in PCA and share genomic clusters, suggesting genetic drift and selection have not separated western hemisphere populations from old world progenitor groups. This finding differs markedly from other livestock species^17–20^.

Previous studies also showed a weak structuring of goat breeds^7,8,21–23^. Carvalho *et al*.^23^ reported the concept of breed for meat goats might not be relevant for goat production, reinforcing our perspective that many so called breeds are actually landraces at best and panmixia predominates in these genetic reservoirs. In addition, Lenstra *et al*.^10^ suggested pure genetic ancestry was not a prerequisite for goat breeds. The Spanish goat raised predominately in the southern USA seems to typify such an assessment^24,25^. Our results demonstrate how this population shared genomic components (>10%) with dairy breeds (Saanen and Lamancha), Argentinean, Morocco, Iran and *C*. *aegagrus*. It is known that no gene flow has occurred recently between the populations mentioned due to geographic distances, except to Lamancha. Therefore, this genomic sharing represents old (400–500 years ago) admixture events that remain conserved in the populations. Given these levels of admixture with old world populations suggests the Spanish breed is a genetic diversity reservoir in the western hemisphere.

Angora are unique in the sense that they are the only population in the western hemisphere originating from near the center of domestication. Their history is important, as South Africa and the USA have highly structured mohair industries, which has served to facilitate selection programs for fiber improvement and resulted in the two countries leading global mohair production. Angora in South Africa had higher levels of inbreeding, reflective of their national policy of not importing genetic resources^26^. Conversely the USA had imported South African Angora in the 1980’s which likely decreased inbreeding and is evident in the clustering analysis (Figs 1 and 2).

The Argentinean Angora were developed using imports of USA Angora^26^, thereby explaining their placement in the PCA (Fig. 1), ADMIXTURE clusters (Fig. 2) and Treemix (Fig. 3). In general, there are always strong migrations events between Angora breeds and C. Neuquino_AR to Angora_AR (as indicated by Treemix, Supplementary Fig. S6). Argentinean mohair production is located in northern Patagonia^27^ the same region where C. Neuquino are raised suggesting gene flow between the breeds as the Angora population was developed.

Brazilian goats were distinct based upon the number of monomorphic SNP, high inbreeding coefficients, mean Fst and pairwise Fst with all others populations. McManus *et al*.^28^ showed that Caninde and approximately 70% of Moxoto herds were concentrated in a specific region within a radius of 500 km from the breed’s geographic midpoint. Our results suggest these breeds had high genetic drift and founder effect coupled with inbreeding, which led to a relatively small population size, agreeing with the geographical distribution. In addition, most of the animals sampled are from two Conservation Nucleus where the acquisition of new animals is restricted.

Generally, genetic diversity measures suggested weak population structures, but this does not imply selection is totally absent from the breeds evaluated. Various genes within selected regions have functional roles that were notable in differentiating the populations (Table 2 and Supplementary Tables S5,S7 and S8).

Five significantly selected regions were observed in Moxoto and Caninde, the main breeds raised in Northeast Brazil noted for high temperatures and low humidity^28^. One selected region in chromosome 6 (32.5–37 Mb) were previously observed as a selection signature for hot and arid environments^29^. Another region in chromosome 6 (86.6–94.9 Mb) harbors two genes (*PPEF2* and *SHROOM3*) previously associated with platelet distribution width, mean corpuscular volume and mean corpuscular hemoglobin concentration in swine^30^, which is also related to heat tolerance and parasite resistance^31^.

Angora populations showed five genes within selected regions that were associated with body size, average daily gain, longissimus muscle area and carcass weight (*CCSER1*, *CPEB2*, *NMUR2*, *SPARC*, *DNMT3B*)^,32–37^. Angora goats have been intensely selected for increased mohair production while compromising body weight and potentially lowering their adaptability to sub-optimum conditions^38–40^. South Africa and United States populations shared selected regions (chromosome 17 and 6), which was previously observed as a selection signature for arid environment^29^ and crimp in wool^41^. The region on chromosome 17 (*FGF2*, *IL2*, *IL7* and *IL21)* has been associated with cytokine receptors and cell proliferation^42^ suggesting regions involved with mohair production and environmental adaptability. Therefore, this region can be simultaneously linked to the selection for mohair production and for harsh environments, or also can be a genetic hitchhiking based on the selection for one of the traits.

South Africa and United States population had two distinct selected regions. These could be linked to different environmental constraints or different genetic solutions that can arise to achieve similar phenotypic selection goals^5^.

Angora goats have been bred for mohair production in the United States since the introduction of these animals from Turkey in 1849^43^. The heritability estimates for fleece weight are medium to high (range 0.22 to 0.45 for greasy fleece weight^24,44–46^). Selection for fiber production among Angora_USA during 60’s and 2000’s was substantial^24,43^. These animals are able to continue producing mohair fiber even during periods of feed shortage or nutrient restriction^47^. Therefore, we expected to find a higher number of strong selection signatures in this breed than in local breeds that did not undergo any artificial selection. However, the number of selection signatures found was not very different from other genetic groups or populations. This could be related to the high polygenic nature of the fleece traits, which did not leave strong selection signals in the genome^48^. Moreover, this reinforced the different picture of goat genetic structure in comparison to other livestock species.

Boer have the largest body size of the studied populations and had strong selection signals for traits associated with size and muscularity similar to cattle and sheep^20,49^. Three regions identified in Boer (CHI2: 119.2–119.8, CHI3: 24.1–37.8 and CHI7: 46.3–64.7 Mb) have genes related to meat traits also found in Australian and Canadian Boer^50^. Another region selected on Boer (CHI13) harbors the bone morphogenetic protein 2 (*BMP2*) gene, which plays a role in skeletogenesis, osteoblastic differentiation and limb patterning^51^.

Ear structure in goats is variable with implications for adaptation to heat stress. A selection signature in chromosome 7 was identified in two hapFLK analyses. Brito *et al*.^50^ associated this region with ear size selection on Lamancha animals (short ears). Here, Boer (long ears), Caninde_BR (average ears) and Lamancha showed selective sweep on this region. Interestingly, these three breeds have contrasting ear phenotypes of Brito *et al*.^50^, validating this region as related to ear morphogenesis.

The Fst and hapFLK analysis showed different selected regions probably due to the known differences in these approaches^5^. The Fst approach is more sensitive to bias by genetic drift in populations^52^. The hapFLK is only slightly affected by migration and is not affected by bottlenecks^5^. Depending on the time scale of selection, the causative SNP eventually became fixed while genetic drift gradually reduces the signal-to-noise ratio^53^, which compromises the Fst approach. The two Brazilian breeds (high inbred and drifted populations), for example, did not have any selection sweep identified by Fst, while the hapFLK analyses identified five regions under selection.

Our use of different population sets increase the power of the hapFLK to identify selection signatures, agreeing with Fariello *et al*.^13^ The Argentinean breeds, for example, did not have any selection signature in the runs with groups and 16 populations. In the run with 12 populations, a first soft signal for C. Neuquino was detected. Then, in the run with only Spanish and Argentinean local breeds, the region in chromosome 6 was confirmed as selected on C. Neuquino (Fig. S36) and two other new selected regions appeared.

The comparison of the genome of the wild ancestor Bezoar ibex (*Capra aegagrus*) with the domestic goat (*Capra hircus*) suggested that the population bottleneck associated with the domestication process was not as severe as for other domesticated species^48^. Goat domestication occurred multiple times, which provided a high diversity to the species^54^. Goat populations presented seven mitochondrial haplogroups until Neolithic era. Modern goat populations, otherwise, have predominant mitochondrial haplogroup (haplogroup A) in the world, which also confirms this history of gene flow across different geographical regions^4,55,56^. A study using wild and domestic goats and sheep showed that the average relatedness was 0.859 and 0.823 for sheep and Asiatic mouflon, respectively, while the average relatedness was around 0.915 between the domestic goats, and 0.916 between Bezoar ibex^5^. Therefore, goat populations are more related to each other than are sheep populations.

Alberto *et al*.^5^ observed that the number of positive selections in goats were almost half of what was observed in sheep and goats had several spots with higher diversity in domestic populations than in the wild. Bezoar ibex showed lower nucleotide diversity than Iranian goats and higher inbreeding than Iranian and Moroccan goats^5^. Genetic load was higher in domestic sheep than in mouflon, while in goats the genetic load was significantly higher for wild individuals^5^. These authors concluded that *Capra* and *Ovis* genus showed opposite global patterns of genomic diversity reinforcing our observation of high goat diversity.

Goats in the western hemisphere have maintained substantial genetic diversity with comparable levels found in the species domestication center. A substantial part of genetic variation seen in Iran and Moroccan populations, as well as in *C*. *aegagrus*, was observed in the American populations evaluated. A similar pattern was observed with sheep and microsatellite data^57^. Therefore, despite being brought to Americas around 400 years ago, a strong genetic linkage is still present.

Breeds, by definition, are closed populations with restricted gene flow, phenotypic uniformity and likely a higher inbreeding than expected from outbred populations^15^. While this general definition is applicable for some goat breeds, such as Angora, Saanen and Boer, the majority of goat populations are better described as landraces^58^ rather than breeds. Goats worldwide are generally restricted to small herds with substantial regional/local germplasm exchange between herds and nonuniform approaches to artificial selection strategies^15^; therefore minimizing genetic distinctions between such populations.

Several goat diversity studies highlighted the high levels of polymorphisms and concluded that goats contain more polymorphic sites than other livestock species^3,8,9,22,23^. Lower levels of long range linkage disequilibrium than sheep and cattle has been observed^21^, which also supports the contention that the goats have not been under intense selection. In general, goats are raised without a specific product goal and without a strong breeding control, which contributes to these observations.

Our results reinforce the concept that breed is not an important discrimination criteria in goat genetic diversity, especially for meat type/local goats commonly used throughout the western hemisphere. Genetic linkage among local breeds to centers of domestication was surprising and suggested little genetic differentiation has occurred due to genetic drift and selection. Western hemisphere local breeds are reservoir of genetic diversity awaiting for genetic improvement and research endeavors. As such, the importance of conservation efforts for these genetic resources should be addressed within countries. Our findings represent an important step to address future breeding, conservation and management policies for a specie that is particularly relevant for the sustainability of marginal livestock producing regions of the world.

## Methods

### Samples

We genotyped 244 animals with Illumina Goat 50 K SNP BeadChip (53,347 SNPs)^59^ plus 124 genotypes from previous studies. The dataset consisted of 12 breeds (specialized and local breeds) raised in the western hemisphere (Table 1). Populations sampled in the USA were: Spanish, Lamancha, Boer, and Angora; and were derived from National Animal Germplasm Program’s genetic resource collection. Two Brazilian and five Argentinean local breeds were sampled also from the germplasm conservation efforts of each country. Twenty-eight Saanen were sampled in Costa Rica. Seventy-eight animals from Angora populations (Argentinean and South Africa) were added to the dataset^26^.

Samples with call rate <0.90 were removed. SNP with call rate <0.90 or with MAF = 0.0 were removed and only autosomes SNPs were used. The final number of SNPs after quality control was 48,442 SNP.

In order to remove highly related animals within breeds, a genomic relationship matrix for each breed was calculated using SNP & Variation Suite v8.7 (Golden Helix, Inc., Bozeman, MT, www.goldenhelix.com). One animal of each pair with a genomic relationship higher than 0.25 was removed, reducing the dataset to 267 animals.

Raw data (50 K SNP chip) of NextGen consortium (http://projects.ensembl.org/nextgen/) consisting of 7 samples from *Capra aegagrus*, 9 samples from Iran population and 30 samples from Moroccan goat population were also used. The filtering criteria for this data were less stringent as the objective was to use them as the root and outgroup (reference population) in different analyses. The autosomes SNP were filtered based on SNP call rate (<0.8) and sample call rate (<0.9), yielding 49,051 SNPs. The two datasets were merged, resulting in a final dataset with 313 animals and 48,203 SNPs (Table 1).

No samples were collected for this study; rather they were collected as part of other programs not associated with this study. Therefore, an institutional animal care and use committee license specific for this study was not necessary. All methods were carried out in accordance with guidelines and regulations of each country.

### Genetic diversity

Three analyses (principal components, ADMIXTURE and Treemix analyses) were applied to evaluate the genetic diversity in these goat populations. For these analyses, a stringent filtering criteria were applied to avoid bias related to linked markers. SNP with call rate lower than 0.95 and MAF < 0.05 were removed. Moreover, LD pruning was applied using a window size of 50 SNPs, an increment of 5 and r^2^ >0.5 (CHM method), resulting in 46,214 SNPs.

Principal component analysis (PCA) was carried out in SNP & Variation Suite v8.7 (Golden Helix, Inc., Bozeman, MT, www.goldenhelix.com) and the plotting of the first three components were performed in R 3.4.2 using the scatterplot3D package. The parameters for PCA analysis were set to find the first 10 components, normalizing each marker data by their actual standard deviation, using an additive model and outlier removal up to 5 times, which was considered as more than 6 standard deviations, from 5 components.

Genetic relationships among breeds and the level of admixture were evaluated through a model-based clustering algorithm implemented in the software ADMIXTURE v. 1.3.0. The cross-validation procedure (10-fold) was executed to estimate prediction errors for each K value (from 2 to 22). The value of K that minimizes the estimated prediction error represented the best predictive accuracy. Individual coefficients of membership to each K cluster produced by ADMIXTURE were visualized using the on-line CLUMPAK server with the feature DISTRUCT for many K’s.

The tree-based approach was used to reconstruct historical relationships between the analyzed populations and to test for the presence of gene flow using the TreeMix software^12^. The program was run on the dataset with animals classified in 17 populations, using *Capra aegagrus* as the root. A variable number of migration events (M) from 0 to 20 were tested and the log-likelihood was used to determine the most predictive model.

### Selection signatures

The selective sweeps were identified by two different approaches, Fst and hapFLK statistics. The main groups identified in genetic structure analyses were used further in the selection signatures. The populations were grouped as: Fiber specialized (Angora populations from South Africa and USA), Meat specialized (Boer), Milk specialized (Saanen and LaMancha), Spanish breed, Brazilian local breeds (Moxoto and Caninde), Argentinean local breeds (Criolla Formosena, Criollo de los Llanos, Criollo Neuquino, Colorada Pampeana and Criollo Riojano). Angora_AR was not used due to their recent formation and crossbreeding observed in the previous analyses. Moroccan population was not used for selection signature detection as this was not an objective of this study. *Capra aegagrus* and Iran populations were used in selection signature detection as a reference group (outgroups).

The two tests used are able to detect different selection signature. The FST indicates a difference among groups of individuals in each marker that could be caused by different selection events. FST test detects highly differentiated alleles, where positive selection in a given genome region causes exaggerated frequency differences between populations^50^. The hapFLK is a haplotype FLK based test that identifies selection signatures among hierarchically structure populations^13^. It differs from Fst in order that takes into account the hierarchical structure of the sampling, allowing genetic drift to differ for each population^5^.

Each group had three Fst results from the comparison with: all other groups combined, all specialized breeds (milk, meat and fiber) and the meat and milk specialized breeds. For Brazilian breeds, since they were placed in different clusters in the ADMIXTURE results, we chose to run the Fst analysis for each breed separately as well. Fst comparison between breeds group and wild populations (*C*. *aegagrus* and Iran population) were also performed.

The Fst values were smoothed using the smoothing tool of SNP & Variation Suite v8.7 (Golden Helix, Inc., Bozeman, MT, www.goldenhelix.com) considering the mean asymmetric method. Smoothing process consider a moving average of a certain number of markers. This process is an approximate method when looking for regions where selection is apparent over multiple markers, rather than one-off high values^50^. The number of SNP to be included in the smoothing window in each comparison were determined based on the number of monomorphic SNP in each group and aiming a false discovery rate lower than 0.5 according to Ramey *et al*.^60^ (Supplementary Table S6). For each comparison, smoothed Fst values greater than the average plus three standard deviations were considered to be under selection.

The hapFLK analyses were performed with five population sets (Table 1). First, the six groups were used in order to have the same comparison pattern of the Fst analyses. Then, we moved to evaluation of the populations separately. As this method uses the haplotype estimation, the population groups could be causing some noise and bias in this process. One run used all populations (16), another only with Angora populations, other with the remaining 12 populations and the last one using just Argentinean local breeds and Spanish animals. The *C*. *aegagrus* was used as the outgroup in all runs. These different population sets were applied because, according to Fariello *et al*.^13^, little power is expected from analyses based on genetic differentiation if populations are too distant. Therefore, Angora populations were set apart since they are too distant and could be lowering the detection power of the analysis. Moreover, only Argentinean local breeds and Spanish was evaluated together to have these closely related populations in a specific run.

The hapFLK analysis involves first the generation of a genome wide Reynolds distance matrix to estimate the hierarchical population structure within each population set. To determine the number of haplotype clusters (K) to be used further, several runs of fastPHASE were performed to register the likelihoods. The point where the increase in number of clusters represent a small increase in log-likelihood was selected as the K to use in the hapFLK analysis. Then, the five hapFLK analyses (6 groups, 16 populations, Angora, 12 populations and Argentinean + Spanish) were run using K equal 70, 80, 35, 60 and 20, respectively. The hapFLK statistic was computed as the average across 20 expectation maximization (EM) runs to fit the LD model (–nfit = 20).

The hapFLK software was run chromosome by chromosome and the results merged to a single file. A python script (https://forge-dga.jouy.inra.fr/projects/hapflk) was used to estimate the hapFLK chi-squared density, standardize hapFLK values and calculate the corresponding p-values of hapFLK results. The plots were generated using the R packages ape and qqman. The significant regions (log p-value > 3) were identified and local population trees and haplotype clusters of each regions were plotted. The local population trees used only those SNPs located within the regions of signatures of selection identified to show the breeds undergoing selection.

### Gene annotation

The regions identified in hapFLK methodology were applied in the Causal Variants Identification in Associated Regions (CAVIAR) software^61^. This statistical framework quantifies the probability of each variant to be causal while allowing an arbitrary number of causal variants. In this case, we allowed up to 10 associated variants in each region and selected only ones that showed p-value lower than 0.05. We used the eigen-decomposition based on the correlation matrix between SNPs selected for the analysis^62^.

The causal variants identified with CAVIAR and the significant SNP observed in Fst comparisons were used to identify genes in each region using the Genome Data Viewer in the NCBI platform (http://www.ncbi.nlm.nih.gov/). The genes were identified based on the Annotation Release 102 and ARS1 genome assembly.

The biological functions and pathways in which these genes are involved were assessed using PANTHER (http://www.pantherdb.org/) (Supplementary Tables S7 and S8). Thereafter, a search in the literature and in the Cattle, Pigs and Sheep QTL database (available online at http://www.animalgenome.org) was executed to identify phenotypes known to be affected by variation in the genes located in each significant genomic region.

## Supplementary information

Supplementary material

## Data Availability

The datasets generated and/or analyzed during the current study will be available in the Animal GRIN Repository from USDA (https://nrrc.ars.usda.gov/A-GRIN/).
