# Supplementary material for: New world goat populations are a genetically diverse reservoir for future use
Source: Sci Rep. 2019 Feb 6;9:1476. doi: 10.1038/s41598-019-38812-3 (PMC6365549; doi:10.1038/s41598-019-38812-3)
Supplement: Supplementary file 1 — Supplementary material [file 41598_2019_38812_MOESM1_ESM.pdf]

**Supplementary Information**

# New world goat populations are a genetically diverse reservoir for future use

Tiago do Prado Paim*, El Hamidi Hay, Concepta McManus, Danielle Assis Faria, Maria Lanari, Laura Chaverri Esquivel, María Isabel Cascante, Esteban Jimenez Alfaro, Argerie Mendez, Olivardo Faco, Kleibe Silva, Carlos Mezzadra, Arthur Mariante, Samuel Rezende Paiva & Harvey D. Blackburn*

*corresponding authors. e-mail: tiago.paim@ifgoiano.edu.br; harvey.blackburn@ars.usda.gov

Supplementary Tables S1-S8
Supplementary Figures S1-S38
References

**Supplementary Table S1.** Summary of Fst per SNP for each of the comparisons performed.

| Comparison | Min | Median | Mean | Max | Var | SD | M | 3SD | 2SD |
|---|---|---|---|---|---|---|---|---|---|
| Angora_Special | -0.013 | 0.050 | 0.093 | 0.792 | 0.013 | 0.115 | 12 | 0.437 | 0.322 |
| Angora_Others | -0.009 | 0.043 | 0.076 | 0.725 | 0.008 | 0.091 | 0 | 0.349 | 0.258 |
| AR_Special | -0.012 | 0.018 | 0.042 | 0.527 | 0.004 | 0.061 | 6 | 0.226 | 0.165 |
| AR_Milk/Meat | -0.015 | 0.016 | 0.042 | 0.538 | 0.004 | 0.065 | 33 | 0.236 | 0.171 |
| AR_Others | -0.011 | 0.011 | 0.030 | 0.449 | 0.002 | 0.047 | 0 | 0.169 | 0.123 |
| BR_Special | -0.022 | 0.077 | 0.121 | 0.843 | 0.018 | 0.134 | 8 | 0.523 | 0.389 |
| BR_Milk/Meat | -0.022 | 0.080 | 0.123 | 0.958 | 0.018 | 0.135 | 30 | 0.528 | 0.393 |
| BR_Others | -0.016 | 0.067 | 0.103 | 0.832 | 0.013 | 0.114 | 0 | 0.444 | 0.330 |
| Moxoto_ Special | -0.071 | 0.073 | 0.117 | 0.872 | 0.019 | 0.138 | 9 | 0.533 | 0.394 |
| Moxoto_ Milk/Meat | -0.052 | 0.075 | 0.114 | 0.947 | 0.018 | 0.132 | 32 | 0.512 | 0.379 |
| Moxoto_Others | -0.051 | 0.063 | 0.099 | 0.854 | 0.014 | 0.117 | 1 | 0.451 | 0.334 |
| Caninde_ Special | -0.061 | 0.089 | 0.132 | 0.873 | 0.021 | 0.147 | 10 | 0.572 | 0.425 |
| Caninde_ Milk/Meat | -0.052 | 0.090 | 0.130 | 0.948 | 0.020 | 0.143 | 33 | 0.558 | 0.415 |
| Caninde_Others | -0.042 | 0.077 | 0.113 | 0.844 | 0.016 | 0.125 | 1 | 0.488 | 0.363 |
| Milk_Special | -0.019 | 0.048 | 0.095 | 0.873 | 0.015 | 0.123 | 12 | 0.465 | 0.341 |
| Milk_Meat | -0.039 | 0.077 | 0.130 | 0.929 | 0.025 | 0.159 | 47 | 0.606 | 0.448 |
| Milk_Others | -0.014 | 0.024 | 0.055 | 0.708 | 0.006 | 0.080 | 0 | 0.294 | 0.214 |
| Meat_Special | -0.042 | 0.060 | 0.109 | 0.887 | 0.020 | 0.140 | 12 | 0.528 | 0.388 |
| Meat_Others | -0.031 | 0.055 | 0.095 | 0.870 | 0.015 | 0.122 | 0 | 0.460 | 0.338 |
| Spanish_ Special | -0.031 | 0.004 | 0.025 | 0.533 | 0.003 | 0.055 | 7 | 0.188 | 0.134 |
| Spanish_ Milk/Meat | -0.038 | 0.003 | 0.025 | 0.487 | 0.003 | 0.057 | 35 | 0.197 | 0.140 |
| Spanish_ Others | -0.039 | -0.001 | 0.016 | 0.471 | 0.002 | 0.043 | 0 | 0.143 | 0.101 |
| Special_Local | -0.007 | 0.021 | 0.045 | 0.500 | 0.004 | 0.061 | 0 | 0.229 | 0.168 |
| USA_Wild | -0.036 | 0.006 | 0.030 | 0.742 | 0.004 | 0.063 | 14 | 0.219 | 0.156 |
| AR_Wild | -0.031 | 0.014 | 0.044 | 0.724 | 0.006 | 0.079 | 21 | 0.280 | 0.202 |
| BR_Wild | -0.047 | 0.085 | 0.151 | 0.974 | 0.034 | 0.183 | 133 | 0.700 | 0.517 |

Min: Minimum Fst; Max: Maximum Fst; Var: variance of Fst results; SD: Standard deviations; M: number of missing SNPs in each comparison; 3SD: threshold used for significance in the Fst plots (three times standard deviation above the mean); 2SD: second threshold used for significance in the Fst plots (two times standard deviation above the mean); AR: Argentinean breeds; BR: BRian breeds; USA: United States breeds; Meat: Boer; Milk: Saanen and LaMancha; Angora: Angora breed from USA and South Africa; Special: Boer, Saanen, LaMancha and Angora breeds (breeds considered specialized in production of meat, milk and fiber, respectively); Wild: *C. aegagrus*.

**Supplementary Table S2.** Summary of significant selected regions identified by Fst analyses for Angora breed.

| Chr | Starts (Mb) | End (Mb) | Size (Mb) | Comparison | SNPs[a] | Genes in the region[b] | Overlaps[c] |
|---|---|---|---|---|---|---|---|
| 6 | 67.3 | 69.5 | 2.23 | Angora vs Milk/Meat, Angora vs Others | 7 | TXK, TEC, SLAIN2, SLC10A4, ZAR1, FRYL, OCIAD1, OCIAD2, LOC108636223, CWH43, DCUN1D4, LRRC66, SGCB, LOC106502204, SPATA18, LOC102169845, LOC102179181, USP46, LOC108636224, LOC102170130, RASL11B, SCFD2, TRNAC-GCA, FIP1L1 | Appenzell[1]; Saanen Nubian Boer Rangeland[2] |
| 6 | 81.7 | 83.7 | 2.00 | Angora vs Milk/Meat | 4 | EPHA5, LOC102186101, LOC106502193, TRNAC-GCA | Barki (Hot and arid)[3]; Milk[4] |
| 7 | 45.9 | 47.9 | 2.00 | Angora vs Others | 1 | LOC102175399, LOC102175123, NMUR2, TRNAS-GGA, LOC108636347, LOC102170226, GLRA1, G3BP1, ATOX1, SPARC, FAT2, LOC102191423, TRNAC-GCA, SLC36A2, LOC102174838, GM2A, CCDC69 | LaMancha; Toggenburg; Boer[2] |
| 13 | 60.4 | 62.6 | 2.13 | Angora vs Milk/Meat | 4 | MAPRE1, DNMT3B, SUN5, + 47 genes | Swiss breeds[1]; Saanen[2] |
| 18 | 12.8 | 15.1 | 2.31 | Angora vs Others | 6 | LOC108637958, LOC102187793, LOC108637957, GINS2C18H16orf74, LOC102170467, EMC8, LOC102188434, IRF8, LOC108637959, FOXF1, MTHFSD, FOXC2, FOXL1, LOC106503074, LOC102185297, C18H16orf95, FBXO31, MAP1LC3B, ZCCHC14, JPH3, KLHDC4, LOC102171732, LOC108637960, SLC7A5, CA5A, BANP, LOC102168566, LOC108637961, LOC102168756, ZNF469, ZFPM1 | Saanen[1] |
| 25 | 33.6 | 36.1 | 2.53 | Angora vs Milk/Meat | 10 | 64 genes (LOC genes) | Boer[2] |

Chr: Chromosome; Start, End: beginning and end of the genome window analyzed considering a window of 1 Mb for each side from the last significant SNP. [a]Number of significant SNPs that determined the region; [b]Genes in the region, when the number of genes were greater than 30 are showed only the genes associated with the significant SNPs (SNPs are in the segment of the genes) and the number of others genes in the region are indicated by "+" signal; [c]Overlaps with previous studies (showed by numbers in reference section) of selection signature in goats (showed the name of the population or trait).

**Supplementary Table S3.** Summary of significant selected regions identified by Fst analyses for Argentinean, Spanish, Meat (Boer) and Milk (Saanen and LaMancha) specialized breeds.

| Chr | Starts (Mb) | End (Mb) | Size (Mb) | Comparison | SNPs[a] | Genes in the region[b] | Overlaps[c] |
|---|---|---|---|---|---|---|---|
| 1 | 145.8 | 147.8 | 2.00 | Argentina vs Milk/Meat, Argentina vs Others | 1 | RUNX1, LOC102177758 | Saanen[1] |
| 6 | 48.5 | 50.8 | 2.30 | Spanish vs Specialized, Spanish vs Others | 6 | LOC102186570, LOC102171961, PCDH7 | Appenzell[1] |
| 9 | 50.6 | 52.6 | 2.00 | Argentina vs Milk/Meat | 1 | TBX18 | Boer[2] |
| 11 | 36.7 | 38.7 | 2.00 | Meat vs Others | 1 | SPTBN1, EML6, RTN4, CLHC1, TRNAE-UUC, MTIF2, RPS27A, LOC102176133, CCDC88A, CFAP36, LOC108637143, LOC106502619, PPP4R3B, PNPT1, LOC102190423, EFEMP1, MIR216B, MIR217, CCDC85A | Barki (Hot and arid)[3] |
| 11 | 69.4 | 71.4 | 2.07 | Milk vs Meat/Fiber | 3 | ALK, CLIP4, C11H2orf71, TOGARAM2, WDR43, TRMT61B, SPDYA, PPP1CB, PLB1, FOSL2, LOC102172165, BABAM2 | Barki (Hot and arid)[3]; Cashmere[2] |
| 14 | 12.8 | 14.9 | 2.10 | Spanish vs Milk/Meat | 3 | GDF6, NDUFAF6, PLEKHF2, TRNAS-AGA, LOC102170068, MTERF3, PTDSS1, LOC102183616, SDC2, LOC108637514, LOC108637513, CPQ, TRNAC-GCA | |
| 14 | 31.5 | 33.7 | 2.12 | Argentina vs Milk/Meat, Milk vs Others | 4 | TRNAW-CCA, LOC102185382, TRPS1 | Barki (Hot and arid)[3] |
| 15 | 71.8 | 73.8 | 2.00 | Milk vs Others | 1 | LOC108637685, LOC102175874, LOC102176145, LOC108637591, LOC108637686, LOC108637592, TRNAC-ACA, LOC108637620 | |
| 16 | 0 | 1.99 | 1.99 | Spanish vs Others | 1 | ZC3H11A, SNRPE, + 45 genes | |
| 18 | 13.1 | 15.1 | 2.00 | Spanish vs Specialized | 1 | EMC8, LOC102188434, IRF8, LOC108637959, FOXF1, MTHFSD, FOXC2, FOXL1, LOC106503074, LOC102185297, C18H16orf95, FBXO31, MAP1LC3B, ZCCHC14, JPH3, KLHDC4, LOC102171732, LOC108637960, SLC7A5, CA5A, BANP, LOC102168566, LOC108637961, LOC102168756, ZNF469 | Saanen[1] |
| 18 | 18.9 | 20.9 | 2.00 | Spanish vs Specialized | 1 | ZNF423, C18H16orf78, CBLN1, N4BP1, LOC102183901, LOC108637962, LOC102184184, ABCC11, LONP2, LOC108637963, SIAH1, ABCC12 | |
| 19 | 61.1 | 62.5 | 1.39 | Milk vs Others | 1 | GNA13, AXIN2, CEP112, APOH, LOC108638219, PRKCA, LOC108638200, CACNG5, CACNG4, CACNG1, HELZ, LOC102171821, LOC102172100 | |
| 23 | 48.3 | 48.8 | 0.48 | Argentina vs Others | 1 | KHDRBS2 | Wild[5] |

Chr: Chromosome; Start, End: beginning and end of the genome window analyzed considering a window of 1 Mb for each side from the last significant SNP. [a]Number of significant SNPs that determined the region; [b]Genes in the region, when the number of genes were greater than 30 are showed only the genes associated with the significant SNPs (SNPs are in the segment of the genes) and the number of others genes in the region are indicated by "+" signal. [c]Overlaps with previous studies (showed by numbers in reference section) of selection signature in goats (showed the name of the population or trait).

**Supplementary Table S4.** Summary of significant selected regions identified using hapFLK analyses per breed.

| Chr | Starts (Mb) | End (Mb) | Size (Mb) | SNP[a] | Analyses [b] | Population selected[c] | N genes [d] | Causal variants [e] | Genes associated with causal variants[f] | Overlaps[g] |
|---|---|---|---|---|---|---|---|---|---|---|
| 3 | 26.1 | 30.1 | 4.00 | 78 | 16 p | Boer | 66 | 6 | LOC102182091, HSPB11, PRPF38A, CC2D1B, SSBP3, ORC1 | Boer[2] |
| 5 | 94.3 | 104.8 | 10.4 | 204 | AR and Spanish | C. Pampeana_AR | 172 | 7 | LOC102185252, KLRG1, FAM234B, M6PR, LOC108633341 | |
| 6 | 19.6 | 38.7 | 19.1 | 381 | AR and Spanish | C. Pampeana_AR; C. Riojano_AR | 81 | 5 | CCSER1, GRID2, BANK1 | |
| 6 | 32.5 | 37.0 | 4.52 | 90 | 16 p | Angora_SA; Caninde_BR; Moxoto_BR; Boer | 24 | 7 | SNCA, HERC3, CCSER1 | Barki (Hot and arid)[3]; Nubian[2]; Wild[5] |
| 6 | 85.3 | 96.0 | 10.7 | 225 | AR and Spanish | C. Neuquino_AR | 124 | 4 | RASSF6, SHROOM3 | |
| 6 | 86.6 | 94.9 | 8.30 | 176 | 12 p | Caninde_BR; Moxoto_BR; C. Neuquino_AR | 85 | 8 | LOC106502208, NAAA, PPEF2, RASSF6, SHROOM3, BTC | |
| 6 | 86.9 | 94.9 | 7.9 | 168 | 16 p | Caninde_BR; Moxoto_BR | 81 | 7 | RASSF6, LOC108636281, LOC102179276, FRAS1, ADAMTS3, SHROOM3 | |
| 6 | 106.9 | 116.3 | 9.35 | 140 | Angora | Angora_USA | 71 | 7 | LDB2, TBC1D14, PROM1, CPEB2 | Swiss breeds[1] |
| 7 | 48.1 | 53.5 | 5.38 | 112 | 16 p | Caninde_BR; Boer; LaMancha | 74 | 3 | HTR4, LOC102174022, FBXO38 | LaMancha; Toggenburg; Boer[2] |
| 7 | 46.3 | 64.7 | 18.41 | 385 | 12 p | Caninde_BR; Boer; LaMancha | 208 | 7 | PPARGC1B, NDFIP1, CAMK2A, SPOCK1, LOC102181119 | LaMancha; Toggenburg; Boer[2] |
| 10 | 16.6 | 21.2 | 4.55 | 94 | 16 p | Caninde_BR; LaMancha | 72 | 7 | MED6, MLH3, PCNX1, ABCD4, SIPA1L1 | |
| 13 | 46.4 | 50.5 | 4.16 | 77 | 12 p | Boer | 28 | 9 | BMP2, GPCPD1, MCM8, LOC102175869 | Swiss breeds[1] |

**Supplementary Table S4.** Continuation…

| Chr | Starts (Mb) | End (Mb) | Size (Mb) | SNP[a] | Analyses [b] | Population selected[c] | N genes [d] | Causal variants [e] | Genes associated with causal variants[f] | Overlaps[g] |
|---|---|---|---|---|---|---|---|---|---|---|
| 17 | 27.7 | 31.8 | 4.13 | 82 | Angora | Angora (SA and USA) | 23 | 8 | CTSO, GLRB, FNIP2, RAPGEF2 | Barki (Hot and arid)[3]; Nubian[2] |
| 18 | 43.7 | 48.3 | 4.59 | 91 | Angora | Angora_USA | 145 | 8 | WTIP, LSM14A, PDCD2L, LOC102168757, CAPNS1 | |
| 23 | 43.4 | 48.8 | 5.40 | 119 | 16 p | Angora_SA | 19 | 7 | BMP5, KHDRBS2 | Wild[5] |
| 23 | 42.0 | 48.8 | 6.82 | 150 | Angora | Angora_SA | 26 | 5 | KHDRBS2 | Wild[5] |

Chr: Chromosome; Start, End: beginning and end of the genome window analyzed considering a window of 2 Mb for each side from the last significant SNP. [a]Number of SNPs in the region; [b]HapFLK analysis where the peak was observed. [c]Population that have received selection pressure in the region based on the tree and cluster plot analyses; [d]Number of genes founded in each region; [e]Number of SNPs in the region identified as causal variants using CAVIAR software; [f]Genes associated with at least one SNP causal variant (distant at maximum 500 kb of the SNP). [g]Overlaps with previous studies (showed by numbers in reference section) of selection signature in goats (showed the name of the population or trait).

**Supplementary Table S5.** Genes identified in hapFLK and Fst analyses associated with Quantitative Trait Loci (QTL) in Cattle, Pigs and Sheep QTL database[a].

| C | Population selected - hapFLK / Fst comparison | Gene | Specie | Traits | Ref[b] |
|---|---|---|---|---|---|
| 6 | Angora_SA; C. Pampeana_AR ; C. Riojano_AR | CCSER1 | Cattle | Average daily gain, Metabolic body weight, Quadriceps weight | 6,7 |
| 6 | Caninde_BR; Moxoto_BR; C. Neuquino_AR | PPEF2 | Pig | Platelet distribution width | 8 |
| 6 | Caninde_BR; Moxoto_BR; C. Neuquino_AR | RASSF6 | Pig / Cattle | Teat number / Curd firming rate | 9,10 |
| 6 | Caninde_BR; Moxoto_BR; C. Neuquino_AR | SHROOM3 | Pig | Mean corpuscular volume; Mean corpuscular hemoglobin concentration | 8 |
| 6 | Angora_USA | CPEB2 | Cattle | Stature | 11 |
| 17 | Angora (SA and USA) | RAPGEF2 | Pig | Teat number | 12 |
| 18 | Angora_USA | CAPNS1 | Cattle | Calpain activity, Marbling score, Quality grade | 13 |
| 23 | Angora | DST | Pig | Femur length, Humerus length, Tibia length, Backfat | 14,15 |
| 23 | Angora_SA Argentina vs Others | KHDRBS2 | Pig | Teat number | 12 |
| 1 | Argentina vs Milk/Meat, Argentina vs Others | RUNX1 | Cattle | Longissimus muscle area, Marbling score | 16 |
| 6 | Angora vs Milk/Meat, Angora vs Others | TXK | Pig | Mean corpuscular volume | 17 |
| 6 | Angora vs Milk/Meat, Angora vs Others | TEC | Pig | Backfat between 3rd and 4th last ribs | 14 |
| 6 | Angora vs Milk/Meat, Angora vs Others | OCIAD1 | Pig | Granulocyte percentage, Platelet distribution width | 8 |
| 6 | Angora vs Milk/Meat, Angora vs Others | OCIAD2 | Cattle | Milk fat percentage | 18 |
| 6 | Angora vs Milk/Meat, Angora vs Others | SCFD2 | Pig | Red blood cell count | 17 |
| 6 | Angora vs Milk/Meat | EPHA5 | Sheep | Wool crimp | 19 |
| 7 | Angora vs Others | NMUR2 | Pig | Average feeding rate | 20 |
| 7 | Angora vs Others | SPARC | Pig | Body width, Front leg conformation, Hind leg conformation, Rib shape | 21 |
| 11 | Milk vs Meat/Fiber | ALK | Cattle | Oleic acid content | 22 |
| 13 | Angora vs Milk/Meat | DNMT3B | Cattle | Body weight, Carcass weight, Dressing percentage, Longissimus muscle area, Marbling score, Subcutaneous fat | 23,24 |
| 18 | Angora vs Others, Spanish vs Specialized | ZCCHC14 | Sheep | Teat number | 25 |

[a]available at www.animalgenome.org. [b]References. C: Chromosome.

**Supplementary Table S6.** Characterization of SNPs in each group used in Fst analysis and the number of SNPs used for smoothing process.

| Groups | SNPs | Mono SNPs[1] | $P_M$[2] | N[3] | $P_N$ mono[4] (%) | FDR[5] | Average gap (kb) | Size[6] |
|---|---|---|---|---|---|---|---|---|
| Fiber (Angora) | 48430 | 520 | 0.01074 | 3 | 0.0001238 | 0.0599 | 49.567 | 99.134 |
| Meat (Boer) | 48430 | 2031 | 0.04194 | 5 | 0.0000130 | 0.0063 | 49.567 | 198.268 |
| Brazil | 48434 | 1582 | 0.03266 | 4 | 0.0001138 | 0.0550 | 49.567 | 148.701 |
| Argentina | 48436 | 80 | 0.00165 | 3 | 0.0000005 | 0.0002 | 49.567 | 99.134 |
| Milk | 48430 | 89 | 0.00184 | 3 | 0.0000006 | 0.0003 | 49.567 | 99.134 |
| Moxoto_BR | 48433 | 3043 | 0.06283 | 5 | 0.0000979 | 0.0473 | 49.567 | 198.268 |
| Caninde_BR | 48432 | 5857 | 0.12093 | 6 | 0.0003128 | 0.1510 | 49.567 | 247.835 |
| Spanish | 48442 | 167 | 0.00345 | 3 | 0.0000041 | 0.0020 | 49.567 | 99.134 |
| USA vs Wild | 48203 | 22 | 0.00046 | 2 | 0.0000208 | 0.0100 | 49.807 | 49.807 |
| AR vs Wild | 48203 | 42 | 0.00087 | 2 | 0.0000759 | 0.0366 | 49.807 | 49.807 |
| BR vs Wild | 48203 | 1566 | 0.03249 | 4 | 0.0001114 | 0.0536 | 49.807 | 149.421 |

[1]Number of SNPs monomorphic (MAF=0) in each group. [2]$P_M$: proportion of monomorphic SNPs in relation to total number of SNPs. [3]N: number (N) of contiguous SNPs for false discovery rate lower than 0.5 (number of SNPs used for the smoothing process in Fst analyses). [4] PN mono: probability (%) that the N (number) of contiguous SNPs are monomorphic by chance. [5]False discovery rate calculated according Ramey et al. (2013), means the number of regions we would expect to find which N contiguous SNPs had fixed alleles. [6]Size of the signature that can be detected in the analysis.

**Supplementary Table S7.** Biological process categories (using PANTHER) for significant hapFLK regions in the group analysis (showing the group that have selection signal for each region).

| Biological process category | Genes | Percent of gene hit against total # genes | Percent of gene hit against total # Process hits |
|---|---|---|---|
| **Meat and Brazil - hapFLK Groups chr 2** | | | |
| cellular process (GO:0009987) | 3 | 20.00% | 21.40% |
| developmental process (GO:0032502) | 3 | 20.00% | 21.40% |
| metabolic process (GO:0008152) | 3 | 20.00% | 21.40% |
| response to stimulus (GO:0050896) | 1 | 6.7% | 7.10% |
| biological adhesion (GO:0022610) | 1 | 6.70% | 7.10% |
| biological regulation (GO:0065007) | 1 | 6.70% | 7.10% |
| localization (GO:0051179) | 1 | 6.70% | 7.10% |
| multicellular organismal process (GO:0032501) | 1 | 6.70% | 7.10% |
| **Meat - hapFLK Groups chr 3** | | | |
| cellular process (GO:0009987) | 569 | 44.30% | 32.60% |
| metabolic process (GO:0008152) | 428 | 33.30% | 24.50% |
| biological regulation (GO:0065007) | 156 | 12.10% | 8.90% |
| developmental process (GO:0032502) | 145 | 11.30% | 8.30% |
| localization (GO:0051179) | 117 | 9.10% | 6.70% |
| response to stimulus (GO:0050896) | 111 | 8.60% | 6.40% |
| cellular component organization or biogenesis (GO:0071840) | 73 | 5.70% | 4.20% |
| multicellular organismal process (GO:0032501) | 71 | 5.50% | 4.10% |
| immune system process (GO:0002376) | 32 | 2.50% | 1.80% |
| reproduction (GO:0000003) | 25 | 1.90% | 1.40% |
| biological adhesion (GO:0022610) | 18 | 1.40% | 1.00% |
| **Brazil - hapFLK Groups chr 7** | | | |
| cellular process (GO:0009987) | 622 | 47.00% | 34.00% |
| metabolic process (GO:0008152) | 456 | 34.40% | 24.90% |
| biological regulation (GO:0065007) | 141 | 10.60% | 7.70% |
| response to stimulus (GO:0050896) | 129 | 9.70% | 7.00% |
| localization (GO:0051179) | 118 | 8.90% | 6.40% |
| developmental process (GO:0032502) | 106 | 8.00% | 5.80% |
| cellular component organization or biogenesis (GO:0071840) | 82 | 6.20% | 4.50% |
| multicellular organismal process (GO:0032501) | 81 | 6.10% | 4.40% |
| biological adhesion (GO:0022610) | 38 | 2.90% | 2.10% |
| immune system process (GO:0002376) | 32 | 2.40% | 1.70% |
| reproduction (GO:0000003) | 15 | 1.10% | 0.80% |
| locomotion (GO:0040011) | 12 | 0.90% | 0.70% |

**Supplementary Table S7.** Continuation…

| Biological process category | Genes | Percent of gene hit against total # genes | Percent of gene hit against total # Process hits |
|---|---|---|---|
| **Meat and Brazil - hapFLK Groups chr 10** | | | |
| cellular process (GO:0009987) | 313 | 56.10% | 36.70% |
| metabolic process (GO:0008152) | 203 | 36.40% | 23.80% |
| response to stimulus (GO:0050896) | 89 | 15.90% | 10.40% |
| localization (GO:0051179) | 85 | 15.20% | 10.00% |
| cellular component organization or biogenesis (GO:0071840) | 58 | 10.40% | 6.80% |
| biological regulation (GO:0065007) | 56 | 10.00% | 6.60% |
| developmental process (GO:0032502) | 48 | 8.60% | 5.60% |
| **Angora - hapFLK Groups chr 23** | | | |
| cellular process (GO:0009987) | 141 | 69.50% | 30.40% |
| metabolic process (GO:0008152) | 112 | 55.20% | 24.10% |
| response to stimulus (GO:0050896) | 63 | 31.00% | 13.60% |
| biological regulation (GO:0065007) | 42 | 20.70% | 9.10% |
| developmental process (GO:0032502) | 29 | 14.30% | 6.30% |
| cellular component organization or biogenesis (GO:0071840) | 28 | 13.80% | 6.00% |
| localization (GO:0051179) | 19 | 9.40% | 4.10% |
| reproduction (GO:0000003) | 16 | 7.90% | 3.40% |
| multicellular organismal process (GO:0032501) | 14 | 6.90% | 3.00% |

**Supplementary Table S8.** Pathways (using PANTHER) of genes found in the significant hapFLK regions in the group analysis (showing the group that have selection signal for each region).

| Pathway | Genes | Percent of gene hit against total # genes | Percent of gene hit against total # Pathway hits |
|---|---|---|---|
| **Meat and Brazil - hapFLK Groups chr 2** | | | |
| Ubiquitin proteasome pathway (P00060) | 2 | 13.3% | 40.00% |
| Integrin signalling pathway (P00034) | 1 | 6.7% | 20.00% |
| Inflammation mediated by chemokine and cytokine signaling pathway (P00031) | 1 | 6.7% | 20.00% |
| Apoptosis signaling pathway (P00006) | 1 | 6.7% | 20.00% |
| **Meat - hapFLK Groups chr 3** | | | |
| TGF-beta signaling pathway (P00052) | 35 | 2.70% | 9.90% |
| FAS signaling pathway (P00020) | 35 | 2.70% | 9.90% |
| Inflammation mediated by chemokine and cytokine signaling pathway (P00031) | 21 | 1.6% | 5.90% |
| Apoptosis signaling pathway (P00006) | 19 | 1.5% | 5.40% |
| Angiogenesis (P00005) | 19 | 1.5% | 5.40% |
| p53 pathway by glucose deprivation (P04397) | 19 | 1.5% | 5.40% |
| Gonadotropin-releasing hormone receptor pathway (P06664) | 19 | 1.5% | 5.40% |
| PDGF signaling pathway (P00047) | 19 | 1.5% | 5.40% |
| Oxidative stress response (P00046) | 19 | 1.5% | 5.40% |
| Ras Pathway (P04393) | 19 | 1.5% | 5.40% |
| B cell activation (P00010) | 19 | 1.5% | 5.40% |
| CCKR signaling map (P06959) | 19 | 1.5% | 5.40% |
| Huntington disease (P00029) | 19 | 1.5% | 5.40% |
| Toll receptor signaling pathway (P00054) | 19 | 1.50% | 5.40% |
| T cell activation (P00053) | 19 | 1.50% | 5.40% |
| Metabotropic glutamate receptor group III pathway (P00039) | 13 | 1.0% | 3.70% |
| Ionotropic glutamate receptor pathway (P00037) | 13 | 1.0% | 3.70% |
| Alzheimer disease-presenilin pathway (P00004) | 9 | 0.7% | 2.50% |
| **Brazil - hapFLK Groups chr 7** | | | |
| Tetrahydrofolate biosynthesis (P02742) | 48 | 8.60% | 32.00% |
| Wnt signaling pathway (P00057) | 152 | 11.50% | 21.30% |
| Parkinson disease (P00049) | 19 | 3.40% | 12.70% |
| De novo purine biosynthesis (P02738) | 88 | 6.60% | 12.30% |
| Alzheimer disease-presenilin pathway (P00004) | 80 | 6.00% | 11.20% |
| Gonadotropin-releasing hormone receptor pathway (P06664) | 16 | 2.90% | 10.70% |
| TGF-beta signaling pathway (P00052) | 16 | 2.90% | 10.70% |
| Wnt signaling pathway (P00057) | 14 | 2.50% | 9.30% |
| Transcription regulation by bZIP transcription factor (P00055) | 14 | 2.50% | 9.30% |
| General transcription by RNA polymerase I (P00022) | 14 | 2.50% | 9.30% |
| Gonadotropin-releasing hormone receptor pathway (P06664) | 61 | 4.60% | 8.50% |

**Supplementary Table S8.** Continuation…

| Pathway | Genes | Percent of gene hit against total # genes | Percent of gene hit against total # Pathway hits |
|---|---|---|---|
| EGF receptor signaling pathway (P00018) | 44 | 3.30% | 6.20% |
| Ubiquitin proteasome pathway (P00060) | 36 | 2.70% | 5.00% |
| Cadherin signaling pathway (P00012) | 32 | 2.40% | 4.50% |
| p53 pathway (P00059) | 31 | 2.30% | 4.30% |
| CCKR signaling map (P06959) | 29 | 2.20% | 4.10% |
| p53 pathway feedback loops 2 (P04398) | 18 | 1.40% | 2.50% |
| p53 pathway by glucose deprivation (P04397) | 18 | 1.40% | 2.50% |
| FGF signaling pathway (P00021) | 18 | 1.40% | 2.50% |
| Parkinson disease (P00049) | 17 | 1.30% | 2.40% |
| TGF-beta signaling pathway (P00052) | 16 | 1.20% | 2.20% |
| Angiogenesis (P00005) | 15 | 1.10% | 2.10% |
| Toll receptor signaling pathway (P00054) | 15 | 1.10% | 2.10% |
| Angiotensin II-stimulated signaling through G proteins and beta-arrestin (P05911) | 14 | 1.10% | 2.00% |
| Interleukin signaling pathway (P00036) | 13 | 1.00% | 1.80% |
| Cell cycle (P00013) | 2 | 0.40% | 1.30% |
| Interferon-gamma signaling pathway (P00035) | 2 | 0.40% | 1.30% |
| Inflammation mediated by chemokine and cytokine signaling pathway (P00031) | 2 | 0.40% | 1.30% |
| EGF receptor signaling pathway (P00018) | 2 | 0.40% | 1.30% |
| **Angora - hapFLK Groups chr 23** | | | |
| DNA replication (P00017) | 17 | 8.40% | 53.10% |
| TGF-beta signaling pathway (P00052) | 15 | 7.40% | 46.90% |

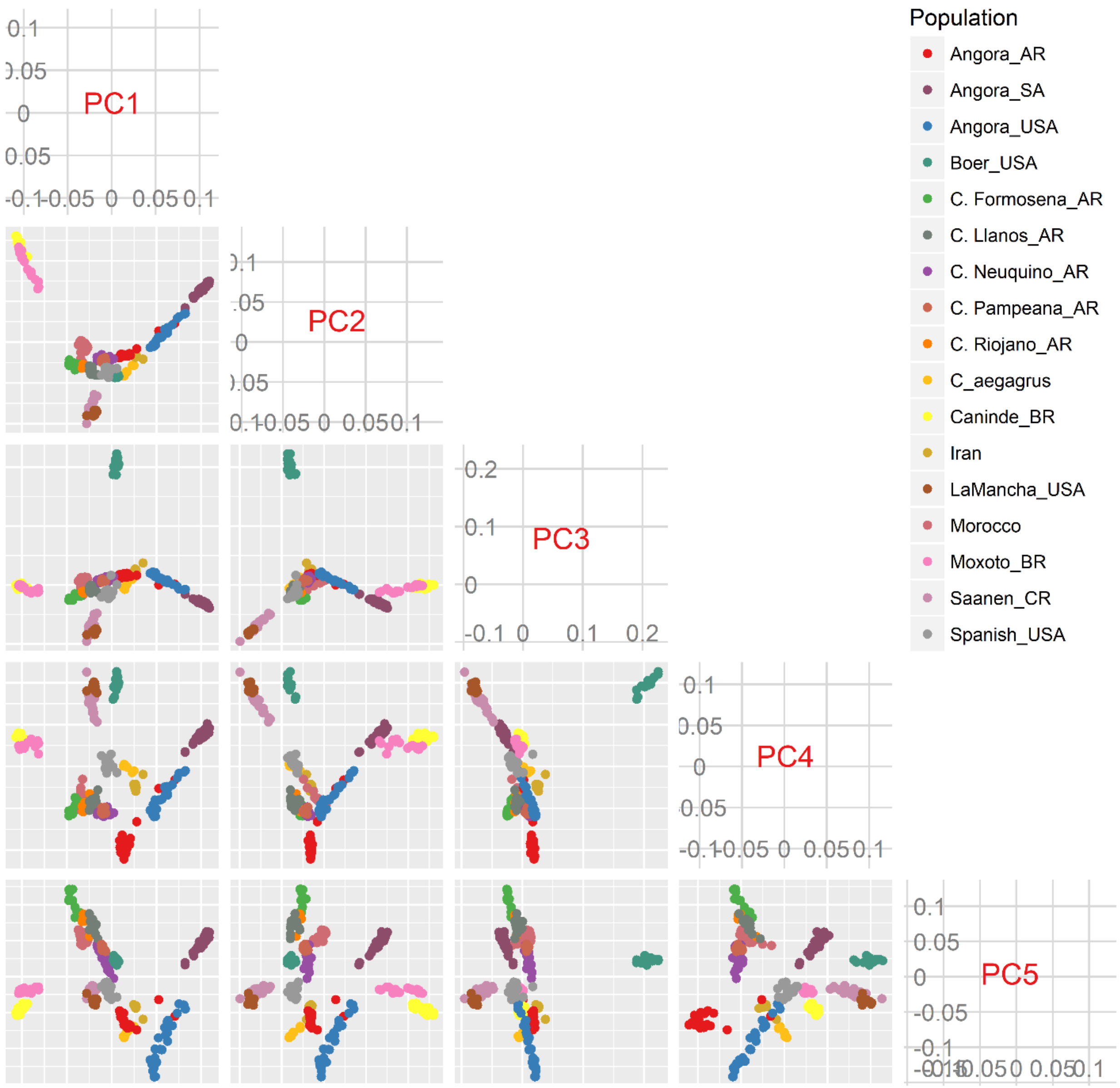


**Supplementary Figure S1**. Scatter plot of the pairwise comparison of the first five principal components using 17 goat populations.

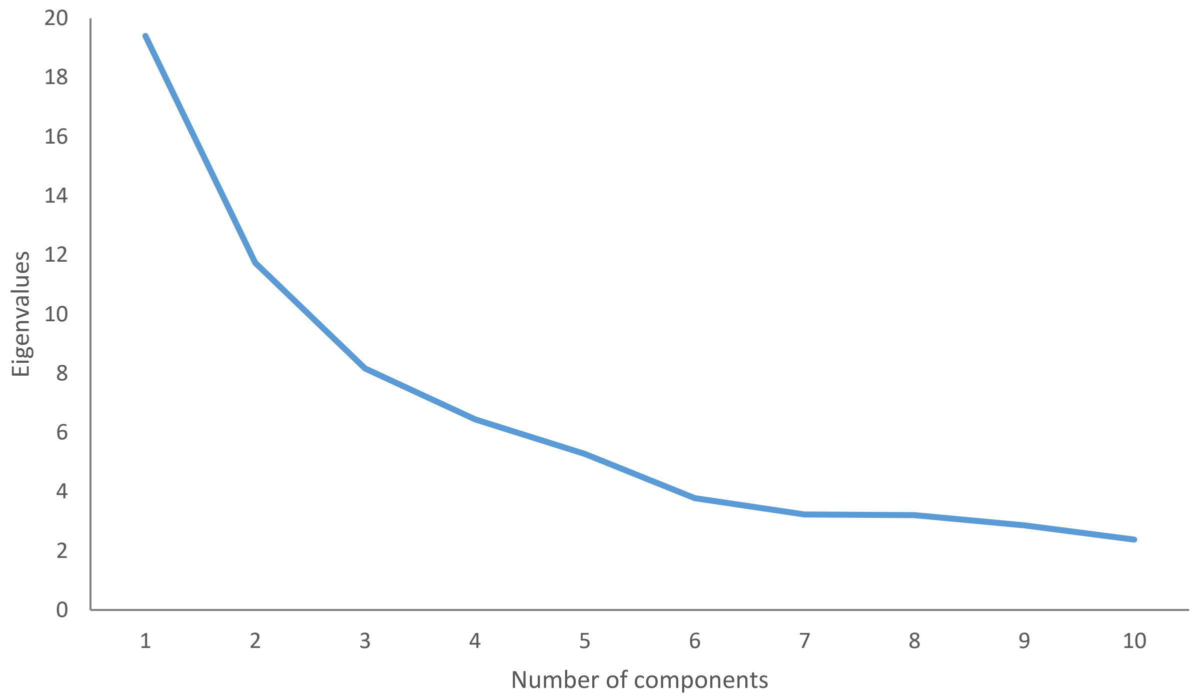


**Supplementary Figure S2**. Eigenvalues of the ten principal components calculated using 17 goat populations.

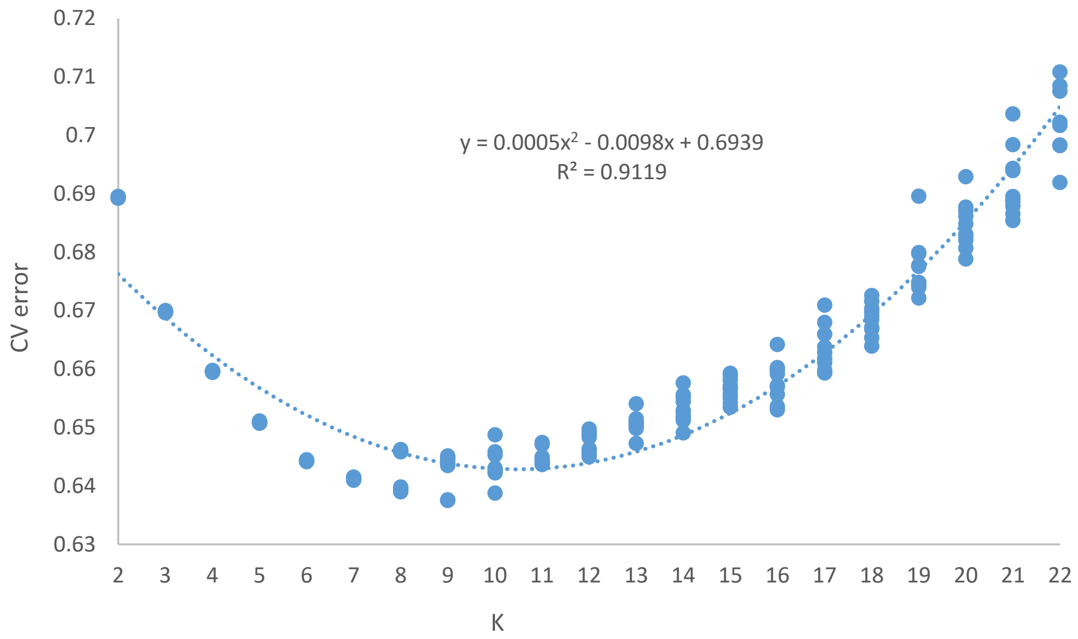


**Supplementary Figure S3**. Cross-validation (CV) error of ADMIXTURE analysis with 10 runs per K.

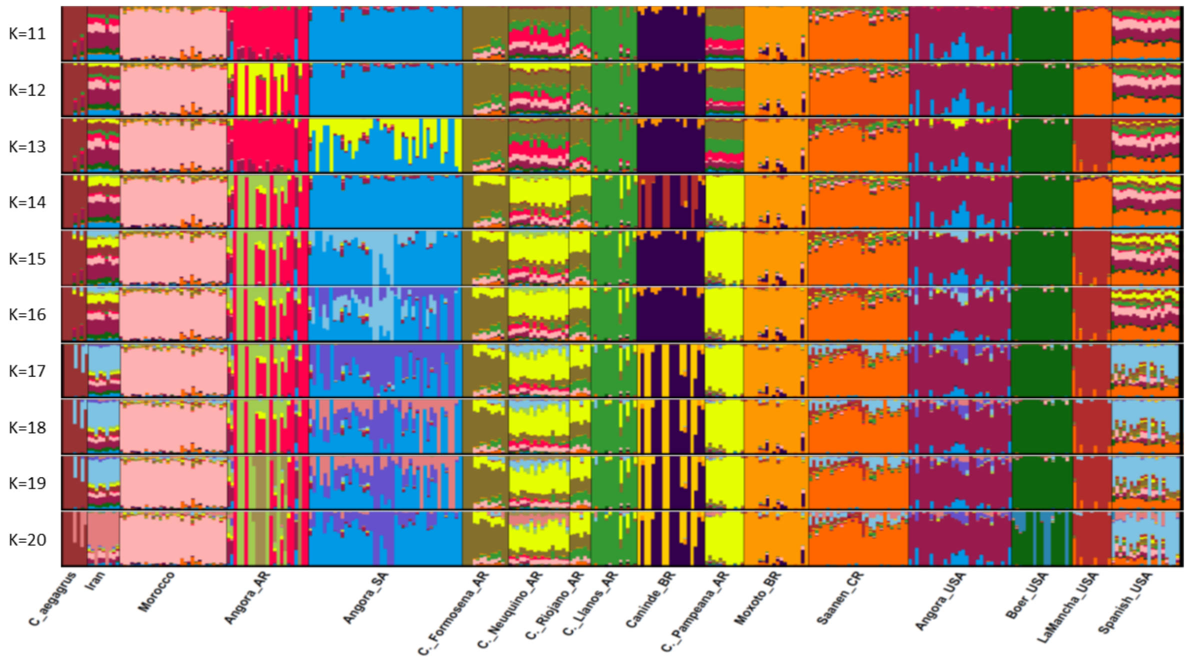


**Supplementary Figure S4**. Clustering plot from K equal 11 to 20 using 17 goat populations.

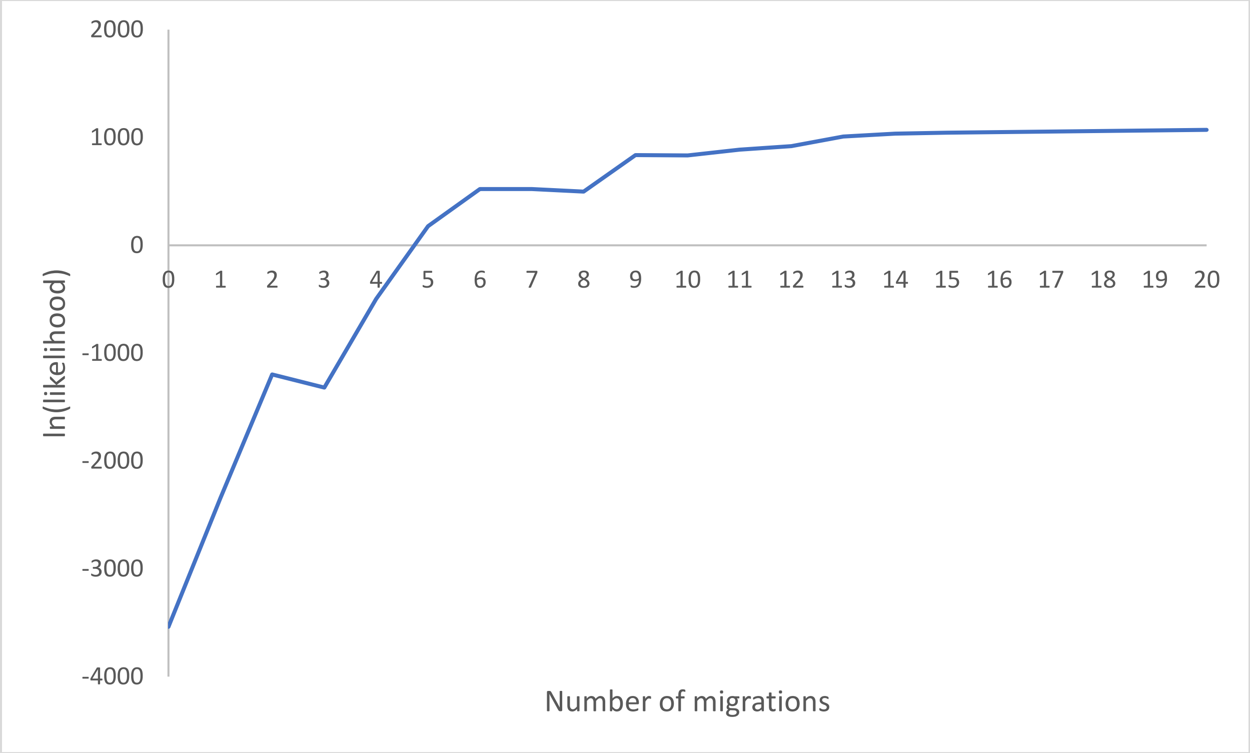


**Supplementary Figure S5**. Logarithm of likelihood of the average between three runs of TREEMIX software simulating 0 to 20 migration events.

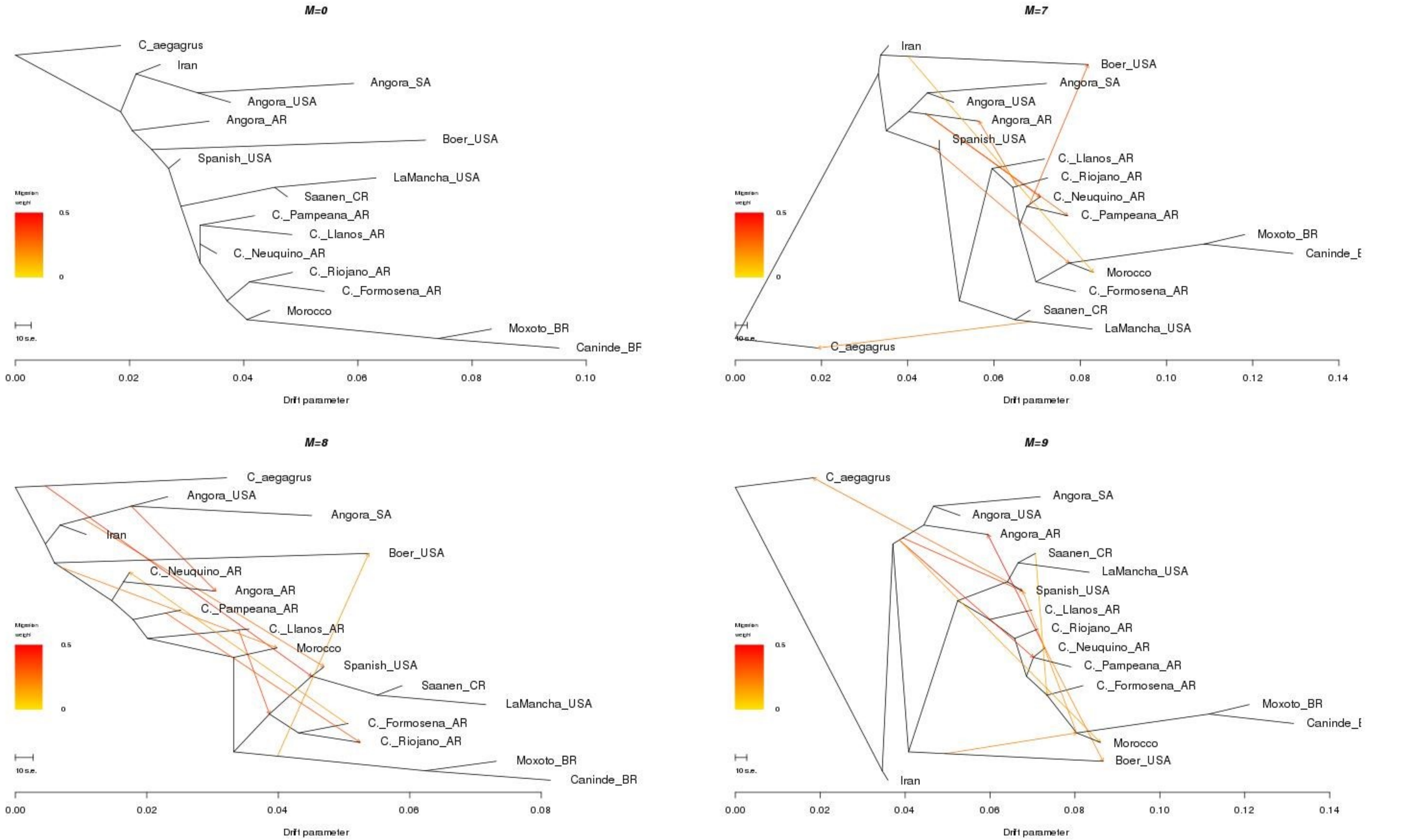


**Supplementary Figure S6**. Population tree (TREEMIX software) using *Capra aegagrus* as root and simulating 0, 7, 8 and 9 migration (M) events.

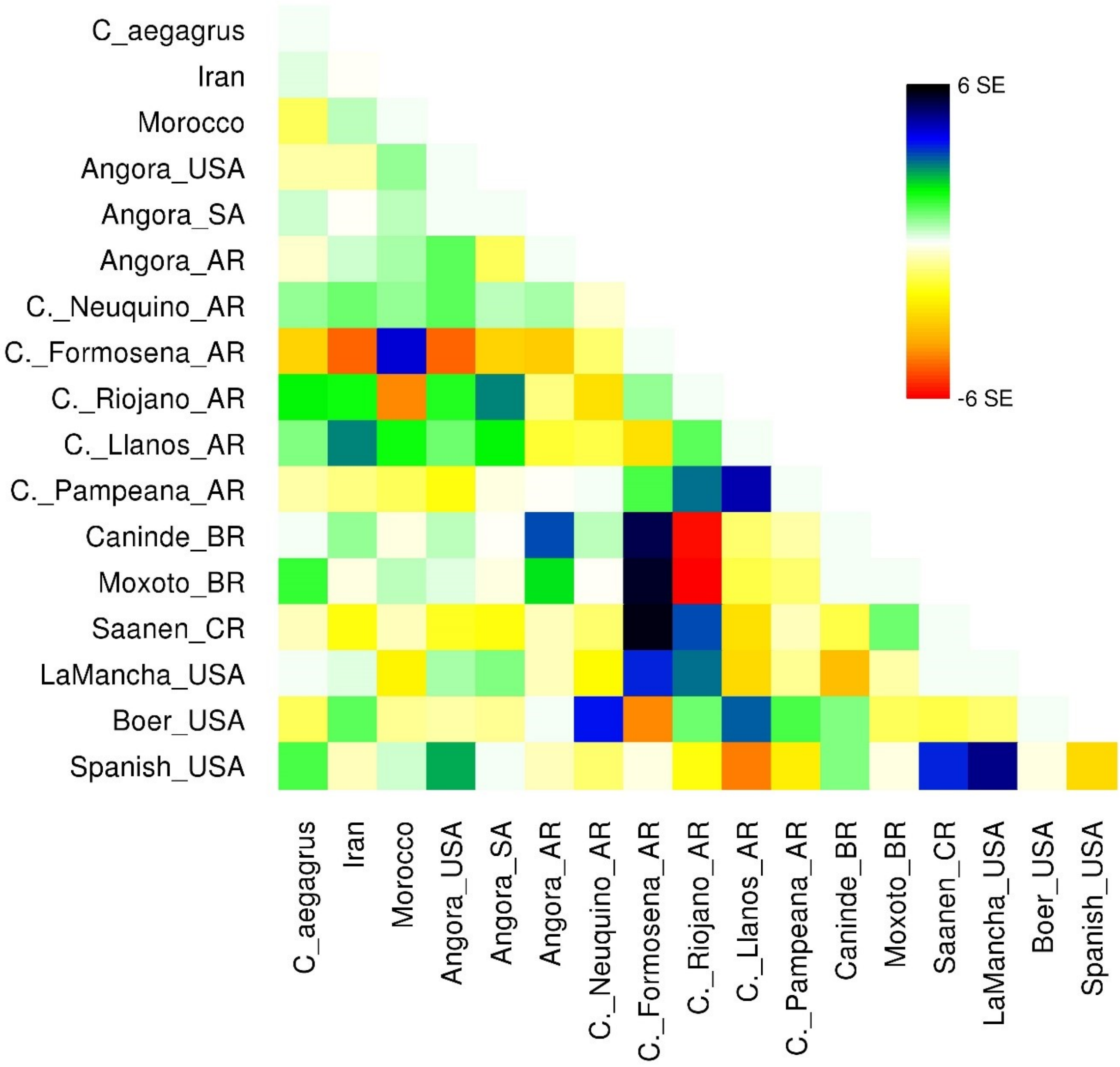


**Supplementary Figure S7.** Pairwise residual values of the model for population tree with six migration events (TREEMIX software) using *Capra aegagrus* as root.

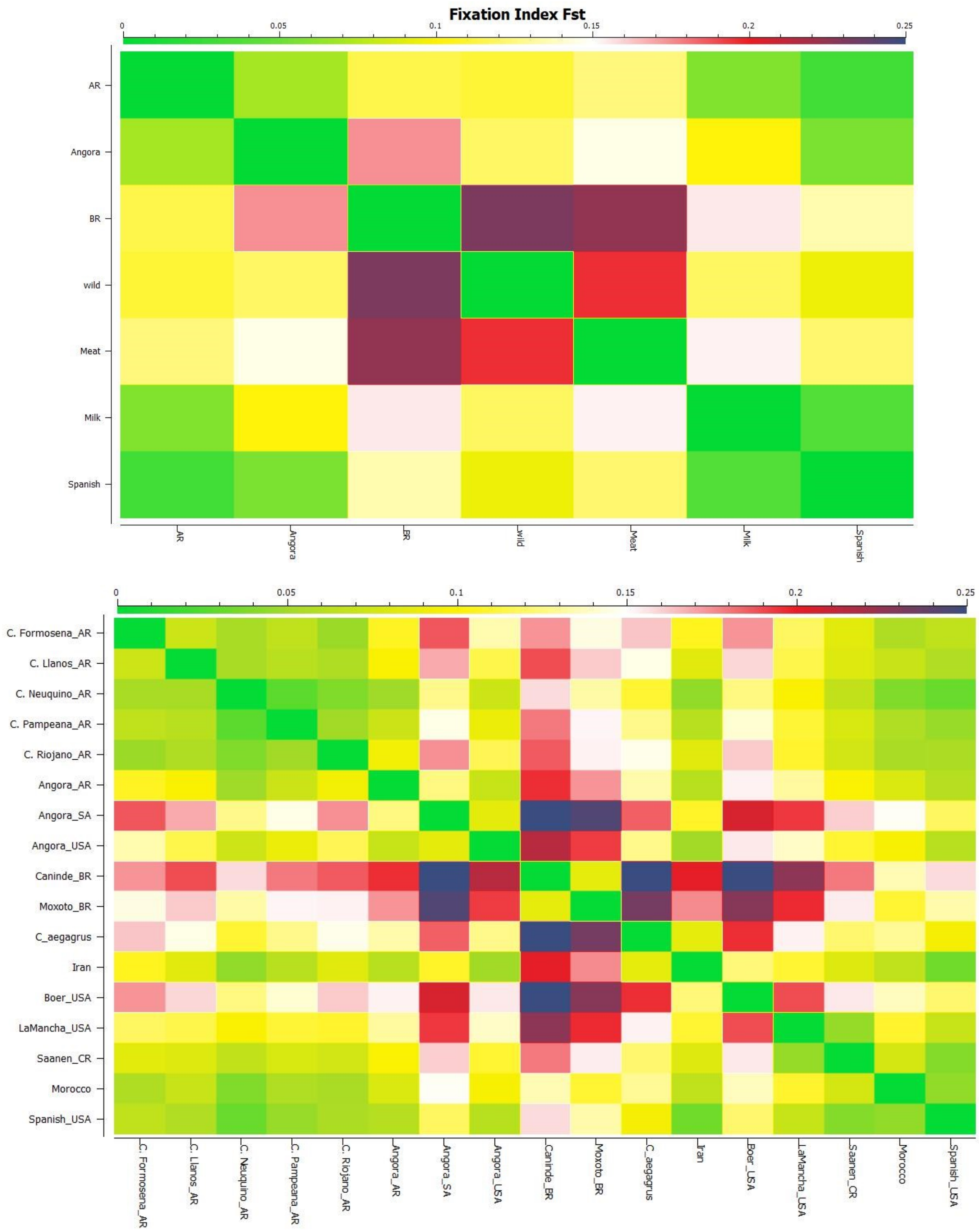


**Supplementary Figure S8.** Pair-wise Fst result comparing the 7 groups of breeds detected in diversity analysis and17 goat populations. AR: Argentinean local breeds; Angora: include only Angora from US and South Africa; BR: Brazilian local breeds; wild: *C. aegagrus*; Meat: Boer; Milk: Saanen and LaMancha.

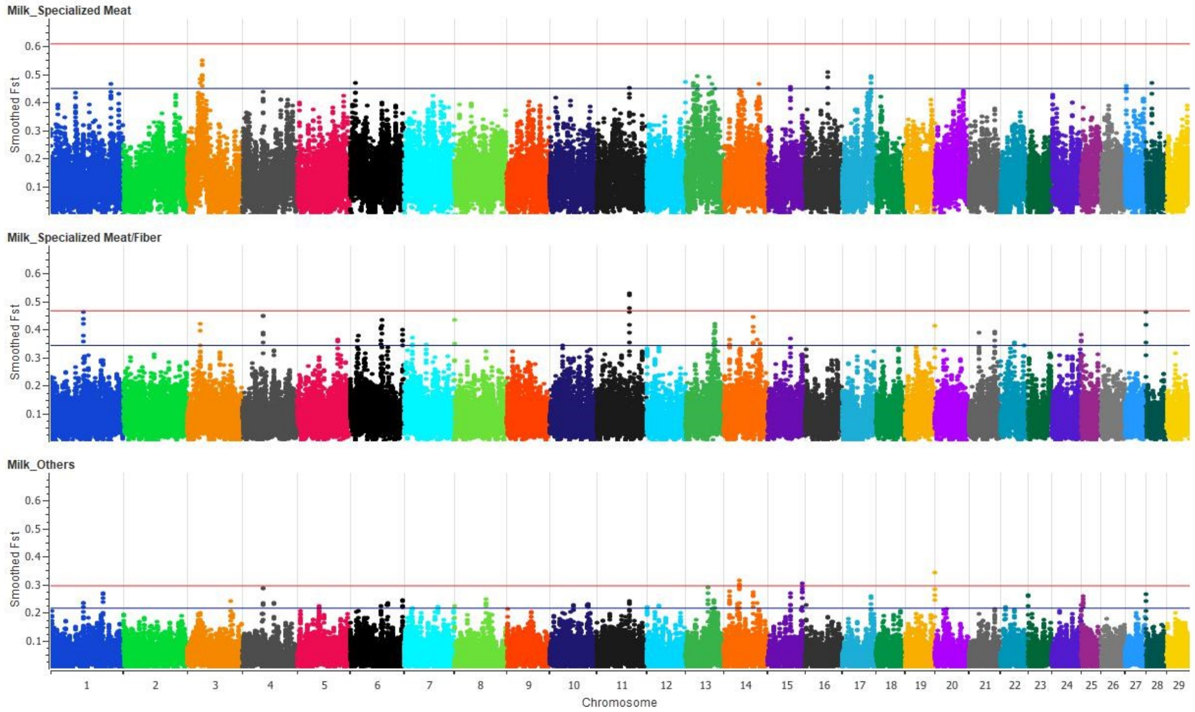


**Supplementary Figure S9.** Smoothed Fst per SP for comparison of milk specialized breeds versus meat specialized breed (Boer), milk specialized breeds versus meat and fiber specialized breeds (Boer and Angora) and milk specialized breeds versus all others goat breeds in the analyses. Red line: significant threshold of three times standard deviation above the mean. Blue line: threshold of two times standard deviation above the mean.

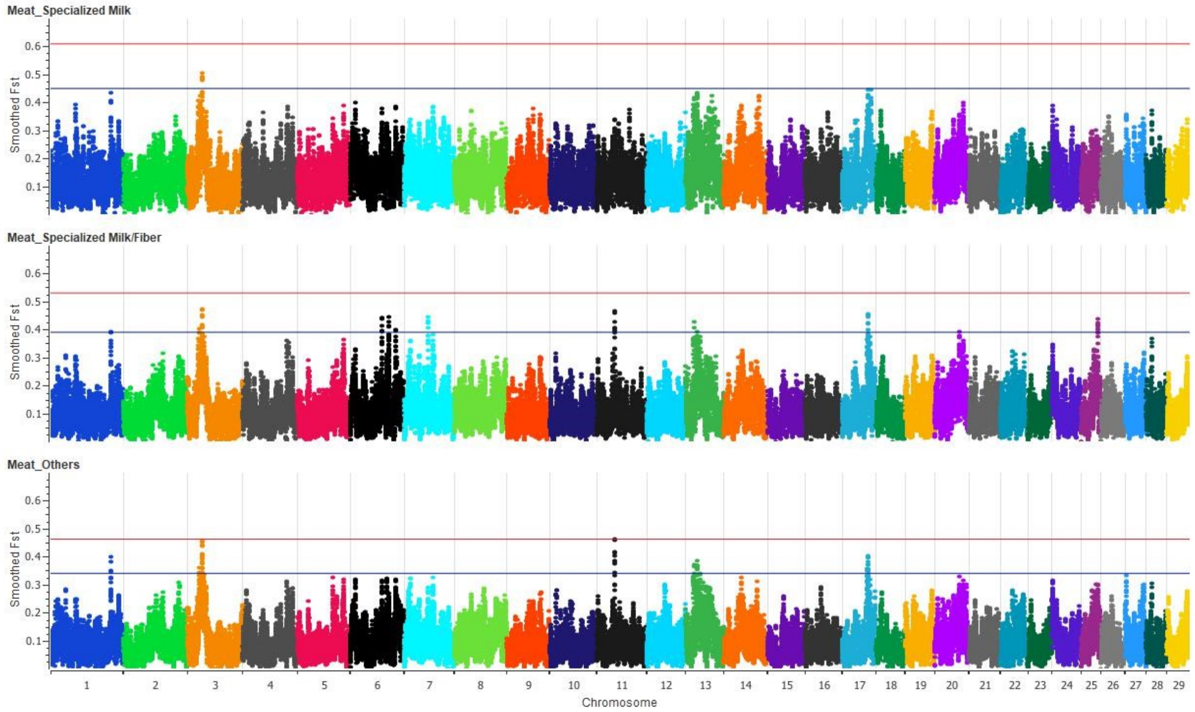


**Supplementary Figure S10.** Smoothed Fst per SP for comparison of meat specialized breed (Boer) versus milk specialized breeds (Saanen and LaMancha), meat specialized breed (Boer) versus specialized breeds (milk and fiber) and meat specialized breed (Boer) versus all others goat breeds in the analyses. Red line: significant threshold of three times standard deviation above the mean. Blue line: threshold of two times standard deviation above the mean.

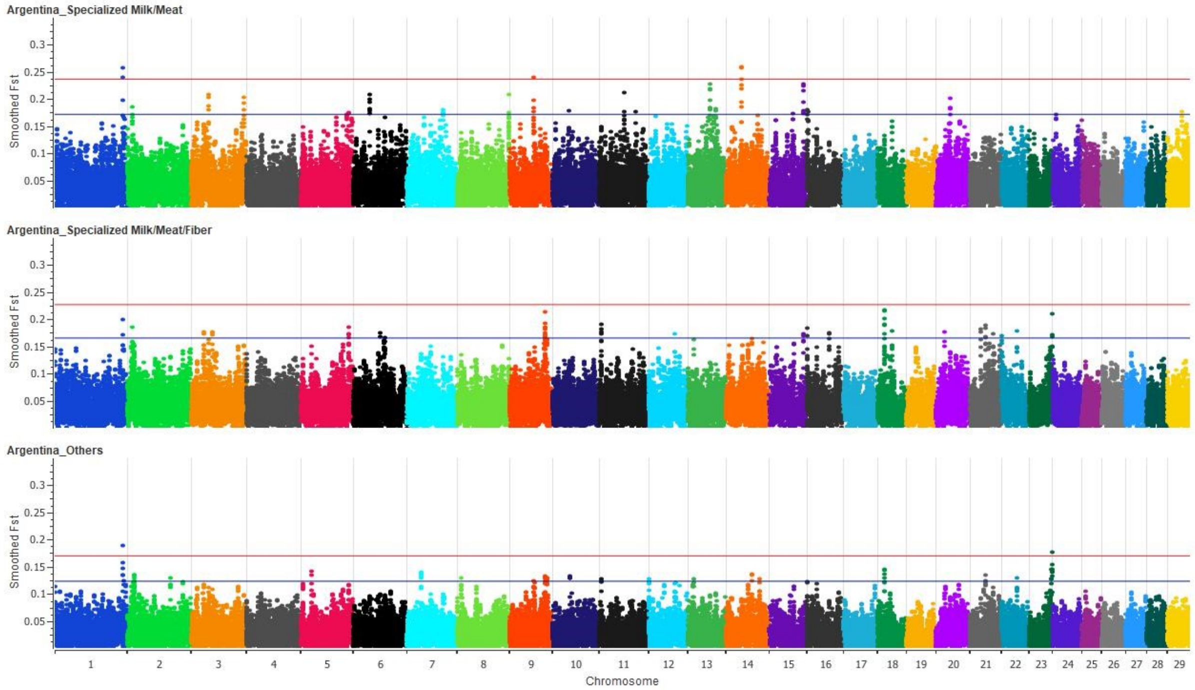


**Supplementary Figure S11.** Smoothed Fst per SP for comparison of Argentinean local breeds versus specialized breeds (Milk, Meat and Fiber), Argentinean local breeds versus Milk and Meat specialized breeds and Argentinean local breeds versus all others goat breeds in the analyses. Red line: significant threshold of three times standard deviation above the mean. Blue line: threshold of two times standard deviation above the mean.

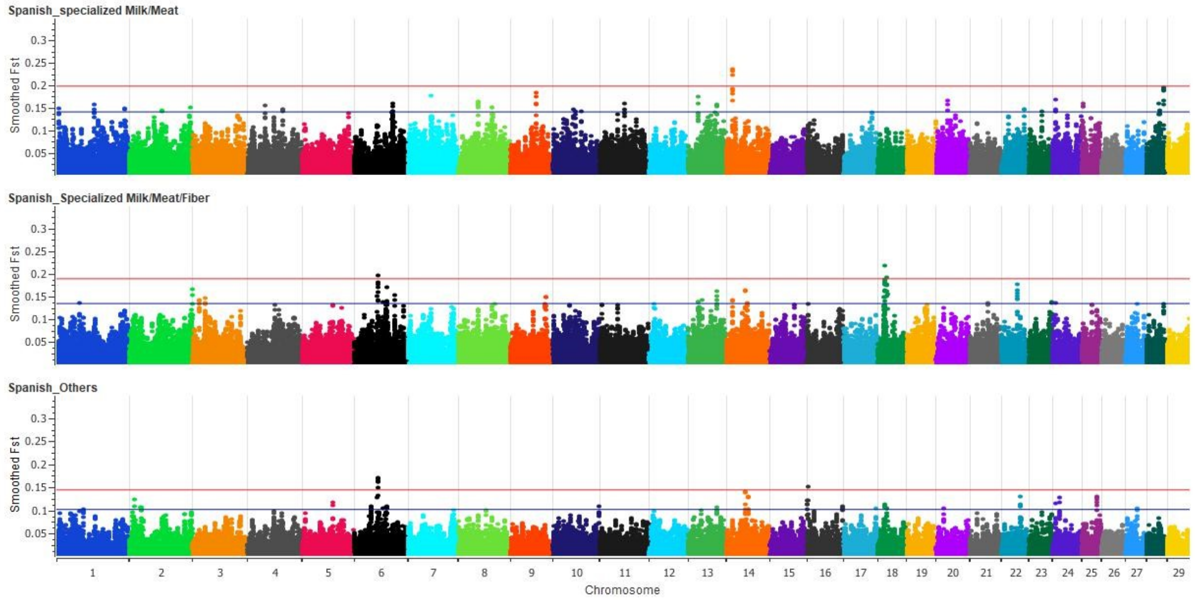


**Supplementary Figure S12.** Smoothed Fst per SP for comparison of Spanish breed versus specialized breeds (milk and meat), Spanish breed versus specialized breeds (milk, meat and fiber) and Spanish breed versus all others goat breeds in the analyses. Red line: significant threshold of three times standard deviation above the mean. Blue line: threshold of two times standard deviation above the mean.

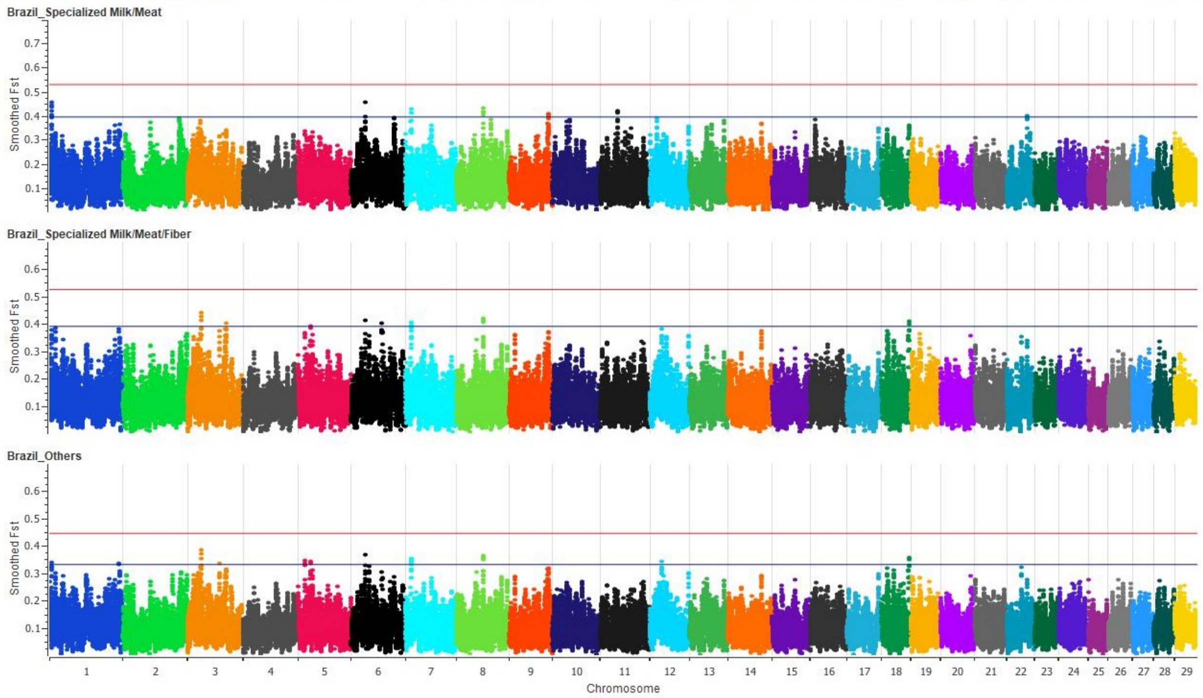


**Supplementary Figure S13.** Smoothed Fst per SP for comparison of Brazilian local breeds versus specialized breeds (Milk and Meat), Brazilian local breeds versus specialized breeds (Milk, Meat and Fiber), Brazilian local breeds versus all others goat breeds in the analyses. Red line: significant threshold of three times standard deviation above the mean. Blue line: threshold of two times standard deviation above the mean.

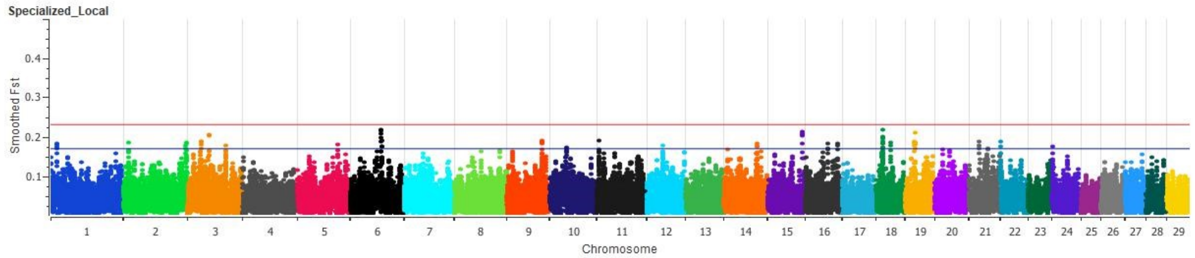


**Supplementary Figure S14.** Smoothed Fst per SP for comparison of local breeds versus specialized breeds (milk, meat and fiber). Red line: significant threshold of three times standard deviation above the mean. Blue line: threshold of two times standard deviation above the mean.

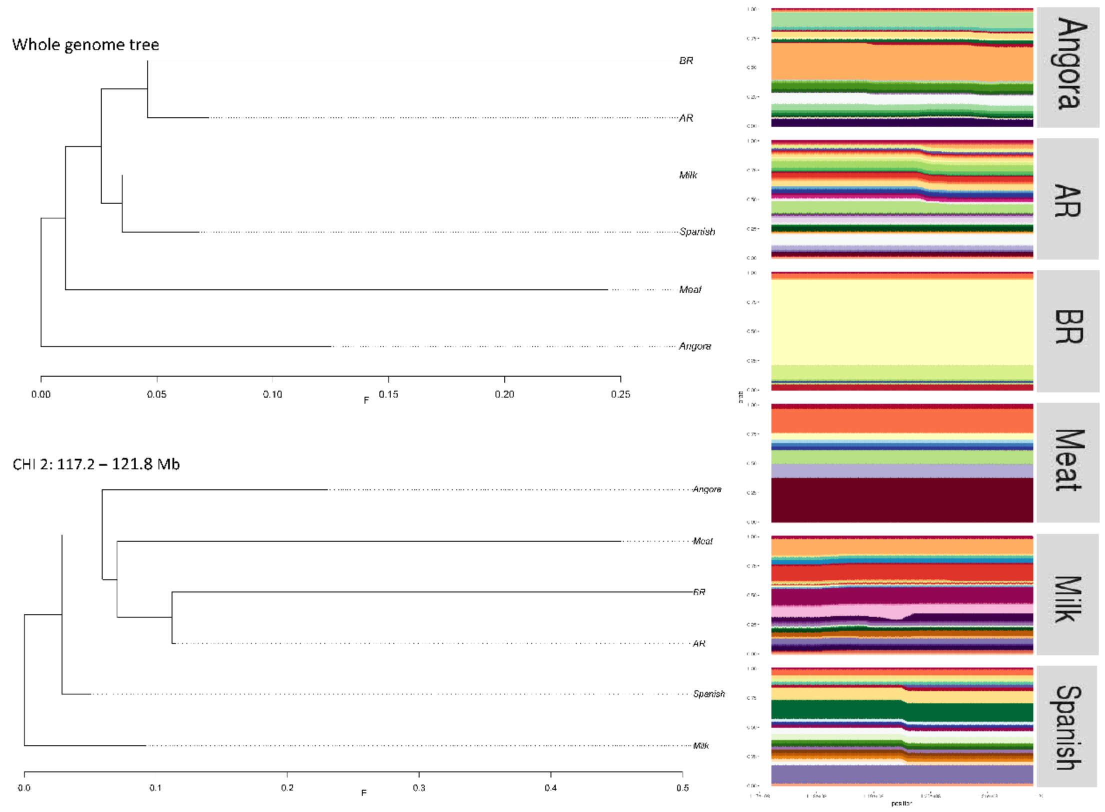


**Figure S15.** Group trees (at left) generated using all available SNPs and only 101 SNPs surrounding the hapFLK peak in chromosome 2 analyzing the six groups. Haplotype clusters frequencies (at right) in the region of chromosome 2 for each group used in the test. AR: Argentinean breeds; BR: Brazilian breeds.

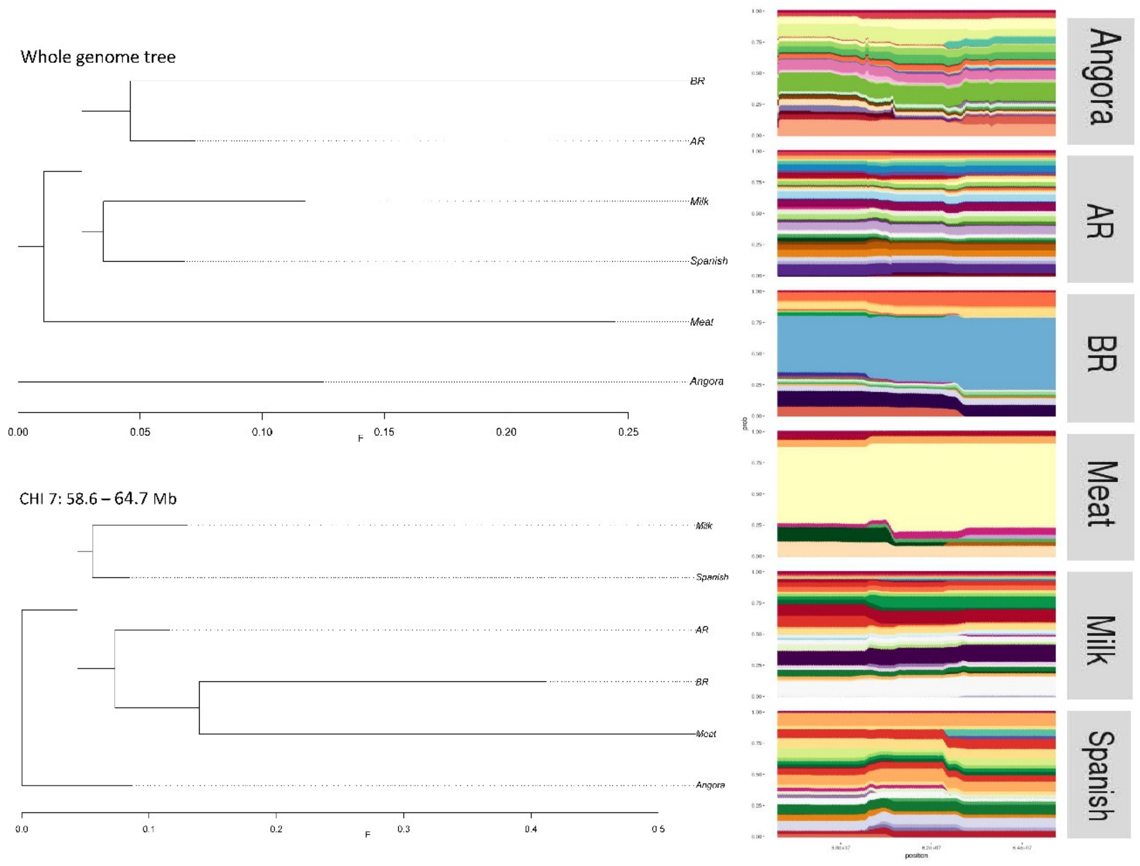


**Figure S16.** Group trees (at left) generated using all available SNPs and only 132 SNPs surrounding the hapFLK peak in chromosome 7 analyzing the six groups. Haplotype clusters frequencies (at right) in the region of chromosome 7 for each group used in the test. AR: Argentinean breeds; BR: Brazilian breeds.

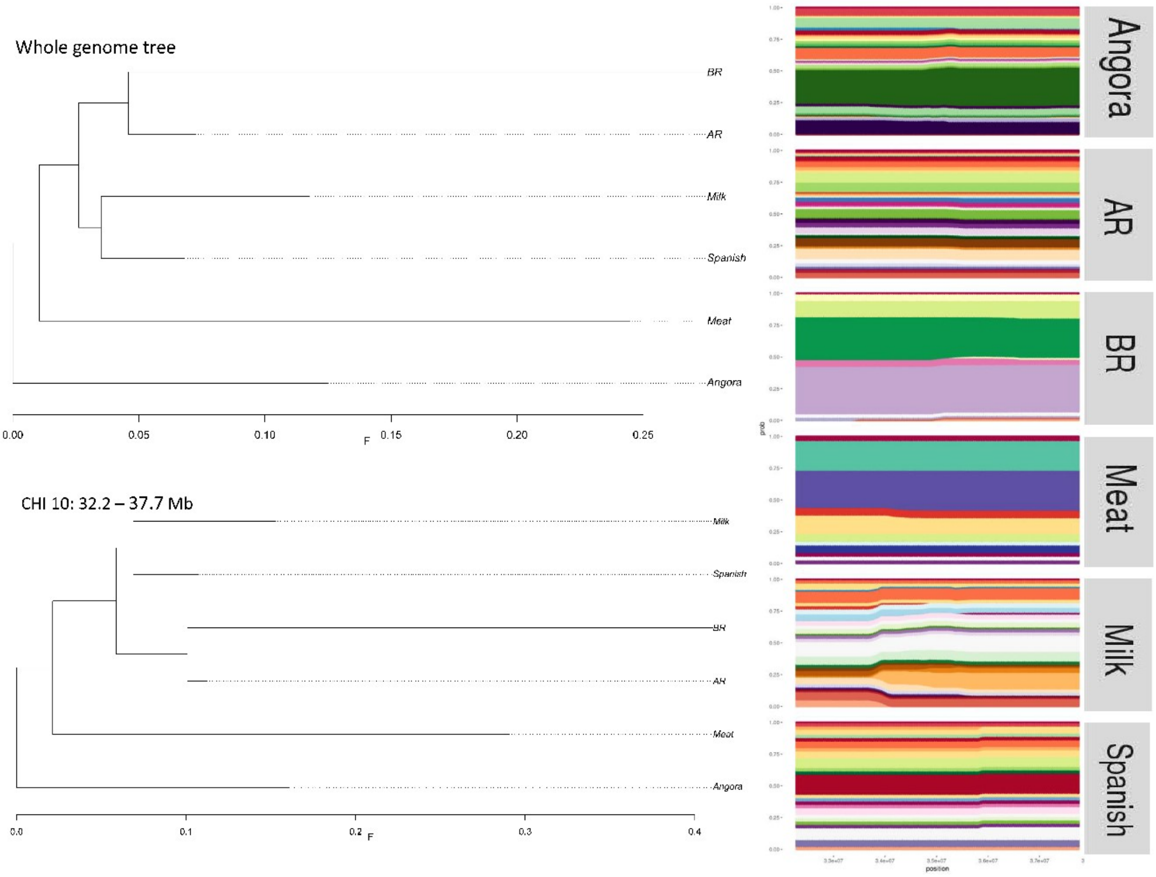


**Figure S17.** Group trees (at left) generated using all available SNPs and only 110 SNPs surrounding the hapFLK peak in chromosome 10 analyzing the six groups. Haplotype clusters frequencies (at right) in the region of chromosome 10 for each group used in the test. AR: Argentinean breeds; BR: Brazilian breeds.

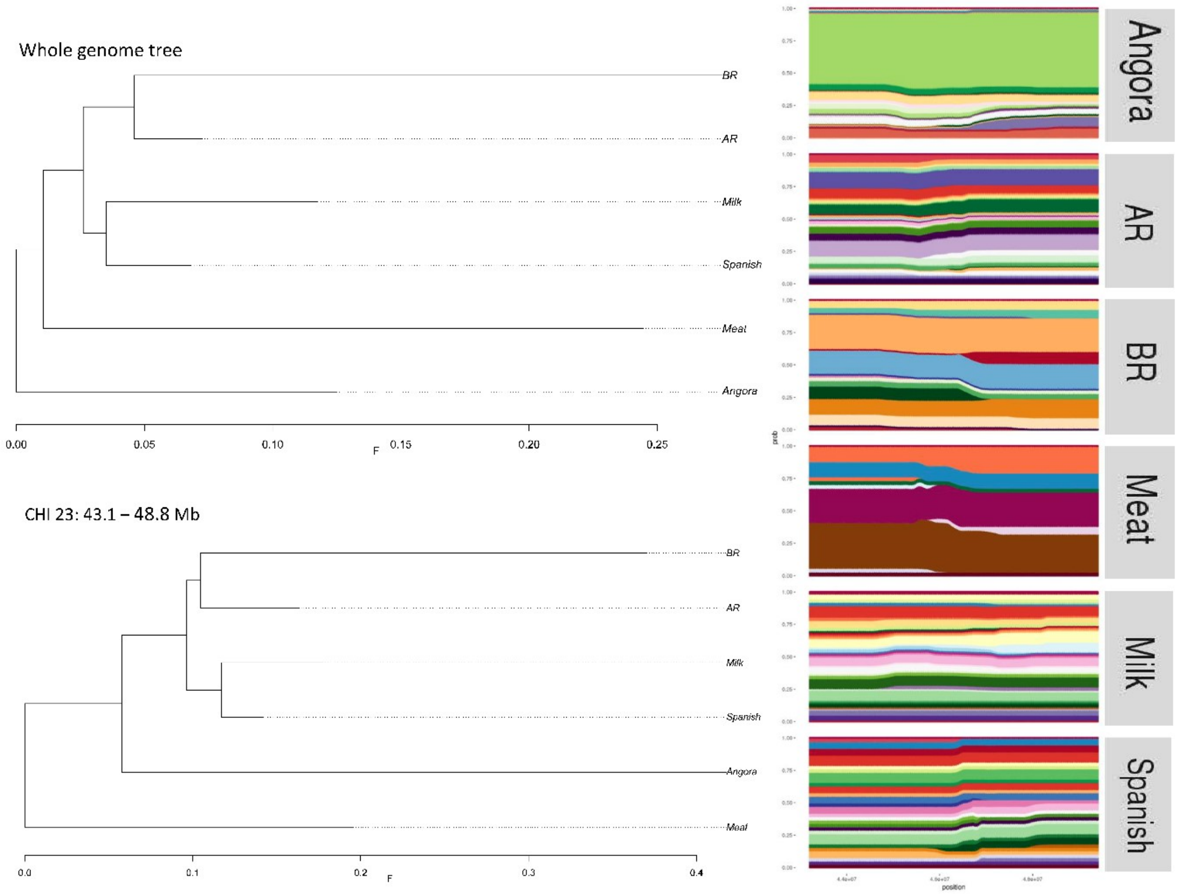


**Figure S18.** Group trees (at left) generated using all available SNPs and only 125 SNPs surrounding the hapFLK peak in chromosome 23 analyzing the six groups. Haplotype clusters frequencies (at right) in the region of chromosome 23 for each group used in the test. AR: Argentinean breeds; BR: Brazilian breeds.

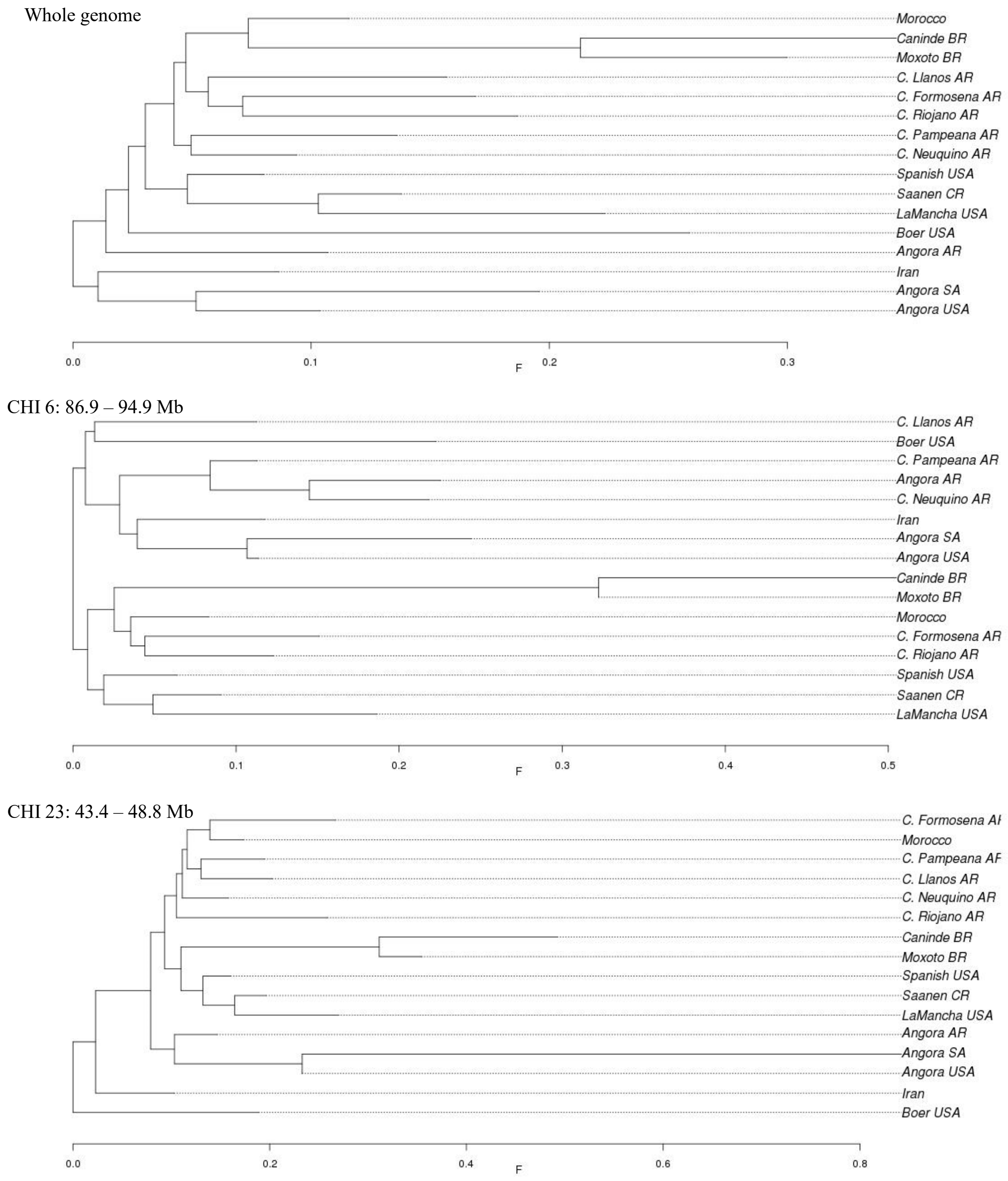


**Supplementary Figure S19.** Population trees generated using all available SNPs, only 168 SNPs surrounding the second peak in chromosome 6 (showing selection pressure in Caninde_BR) and 119 SNPs surrounding the hapFLK peak in chromosome 23 (showing selection pressure in Angora_SA) analyzing all the 16 populations.

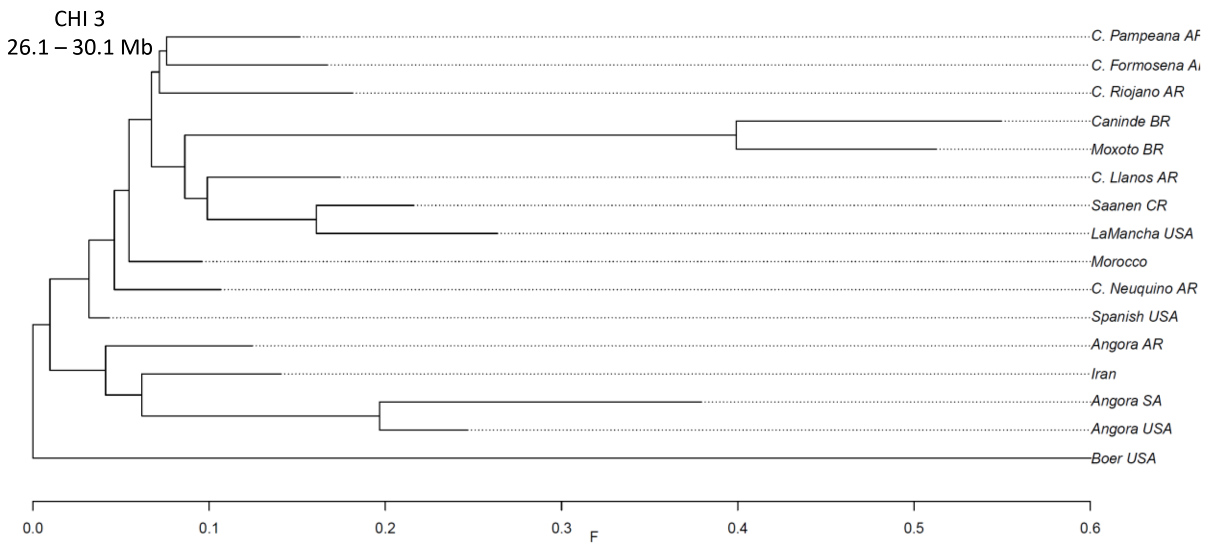


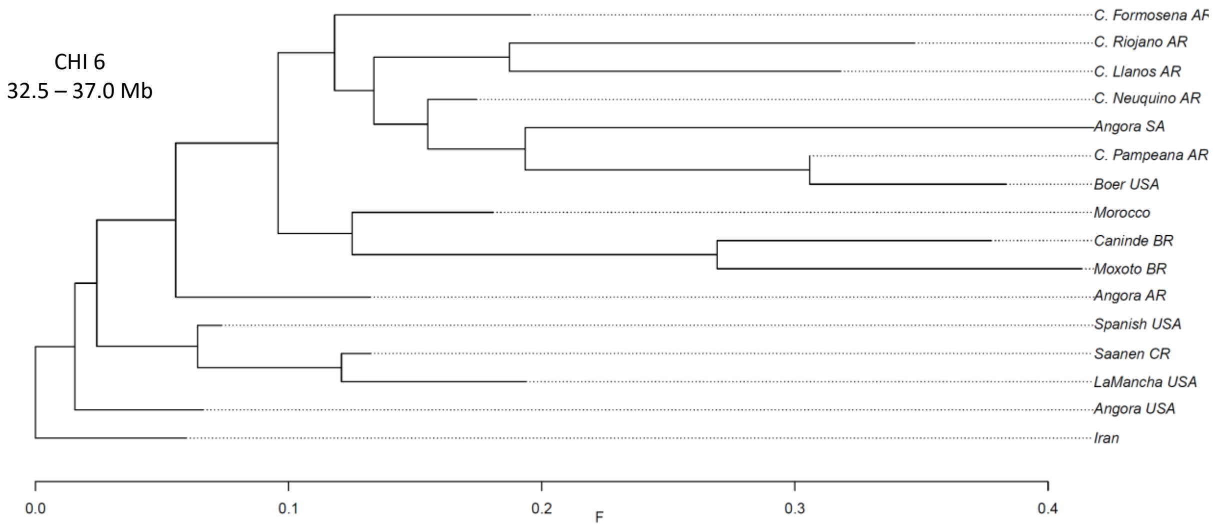


**Supplementary Figure S20.** Population trees generated using only 78 SNPs surrounding the hapFLK peak in chromosome 3 and 90 SNPs in the first peak in chromosome 6 analyzing all the 16 populations.

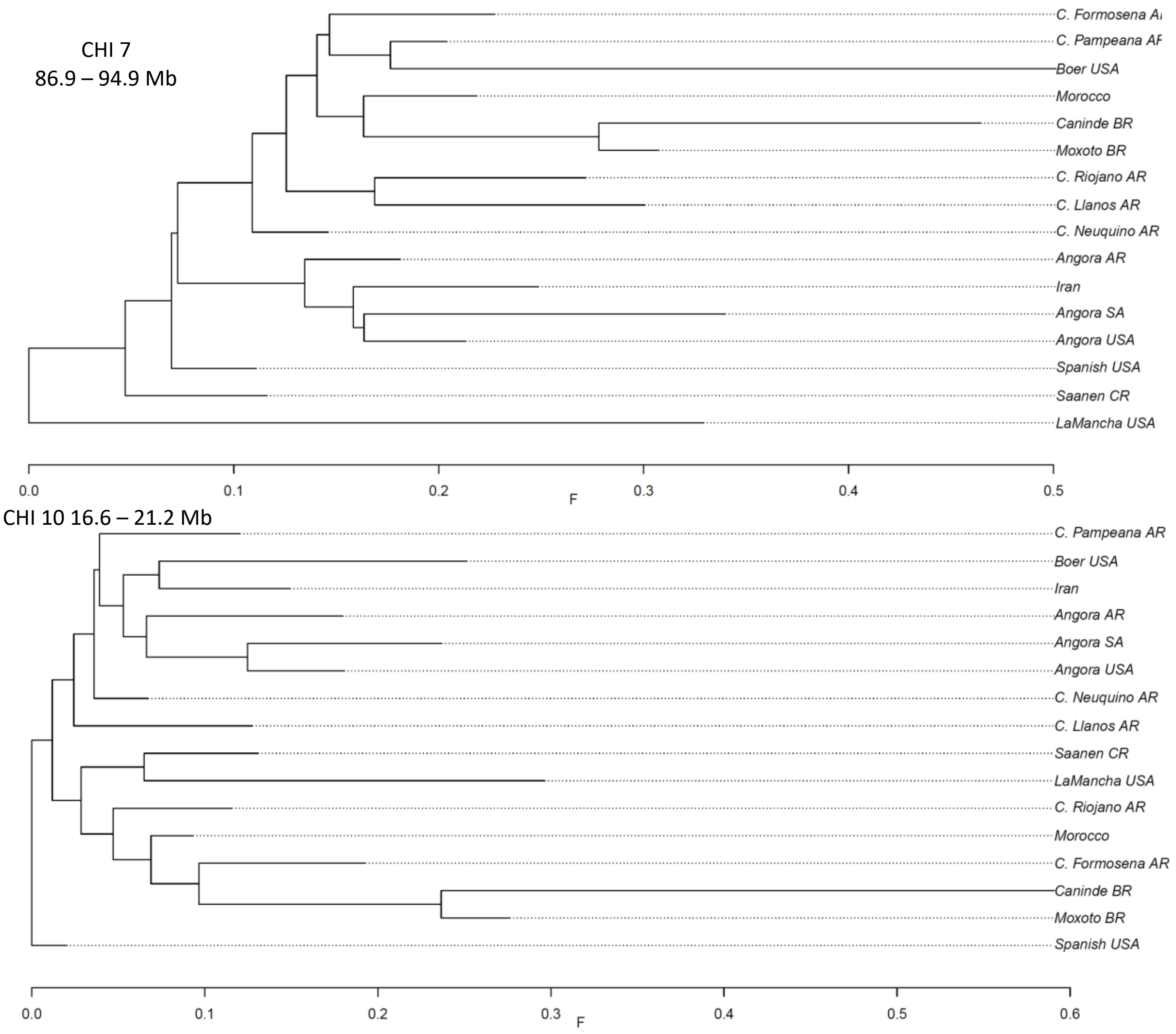


**Supplementary Figure S21.** Population trees generated using 112 SNPs in chromosome 7 and 94 SNPs in chromosome 10 analyzing all the 16 populations.

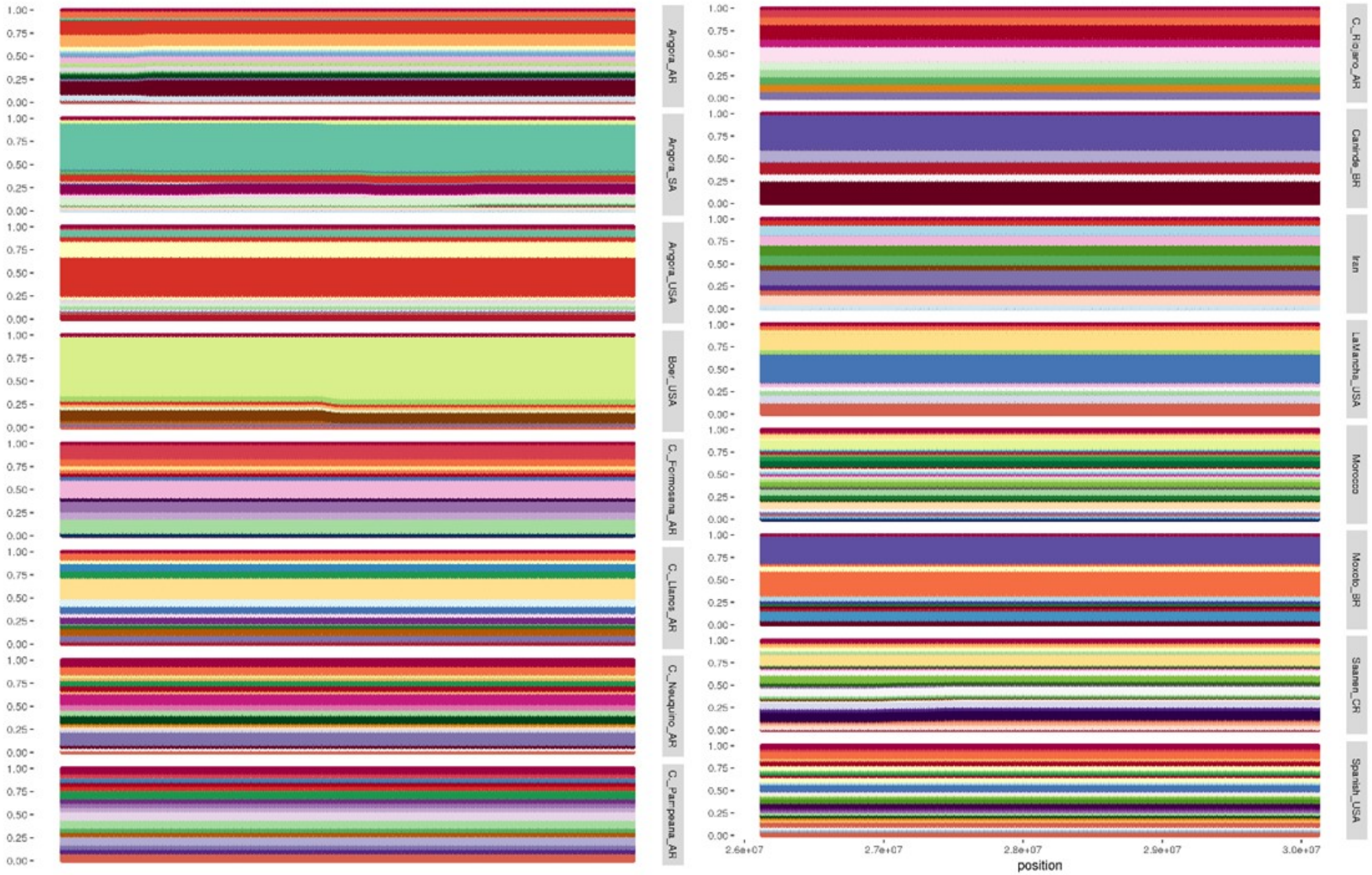


**Supplementary Figure S22**. Haplotype clusters frequencies in detected region in chromosome 3 for each of the 16 goat populations used in the test.

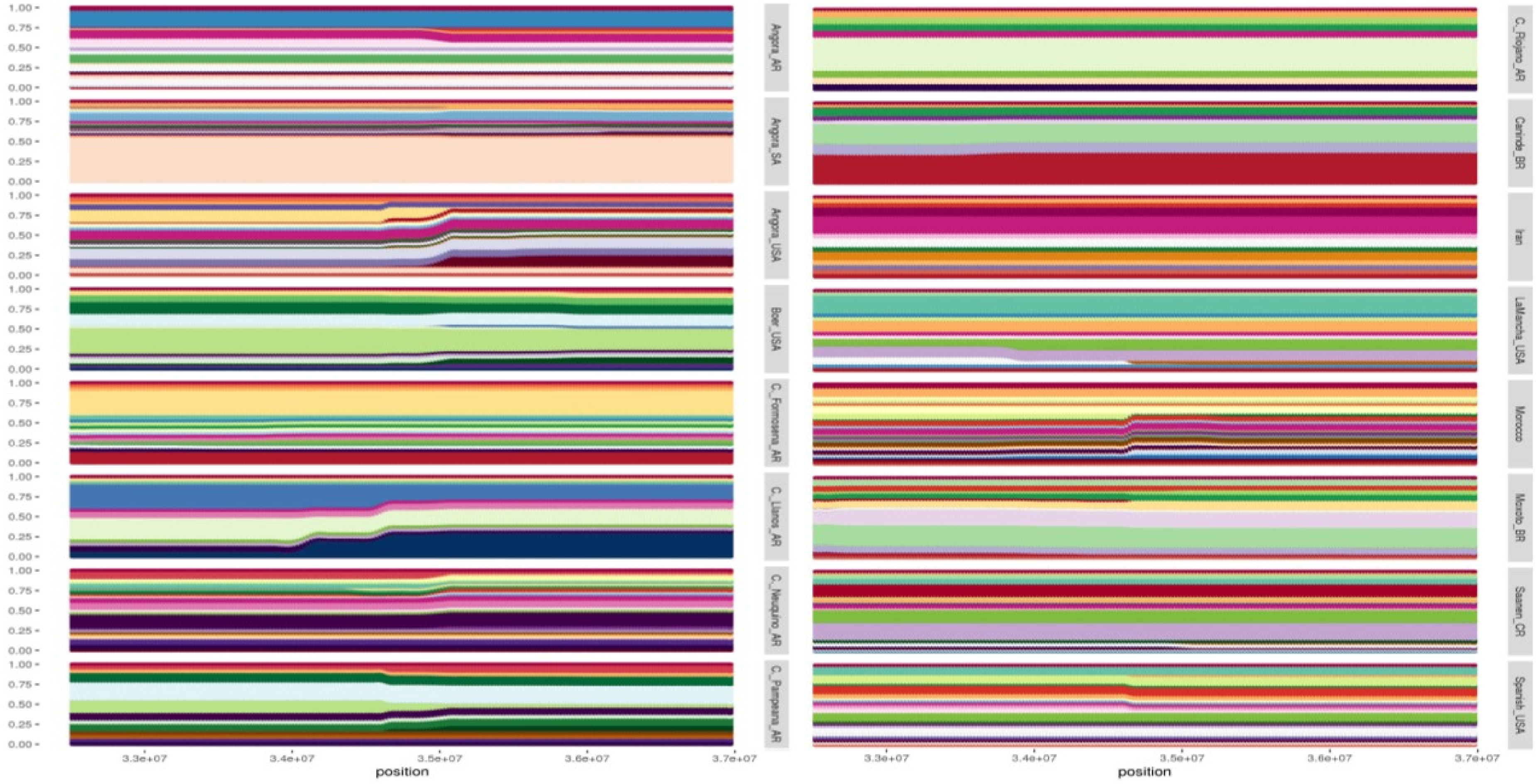


**Supplementary Figure S23**. Haplotype clusters frequencies in the first detected region in chromosome 6 for each of the 16 goat populations used in the test.

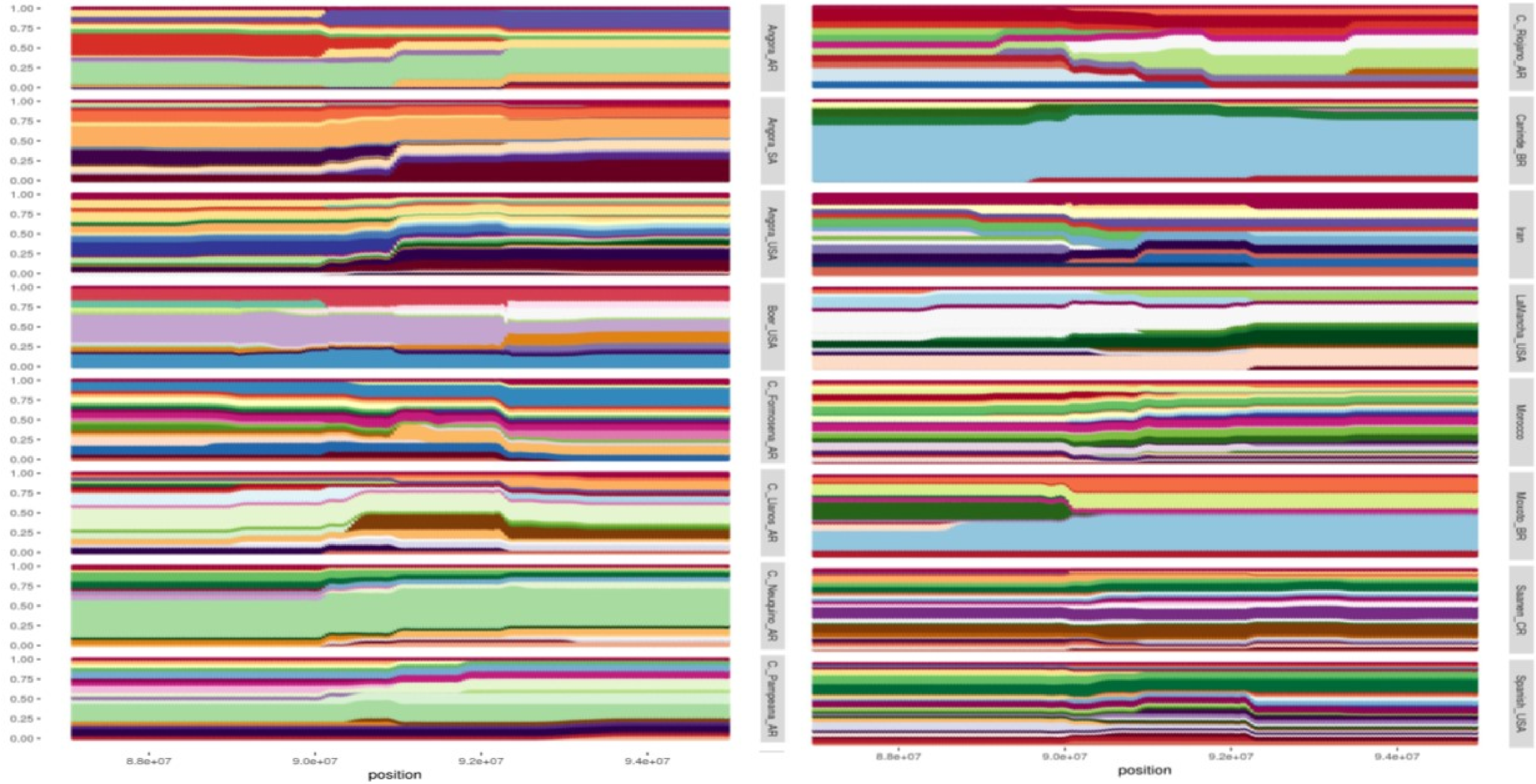


**Supplementary Figure S24**. Haplotype clusters frequencies in the second detected region in chromosome 6 for each of the 16 goat populations used in the test.

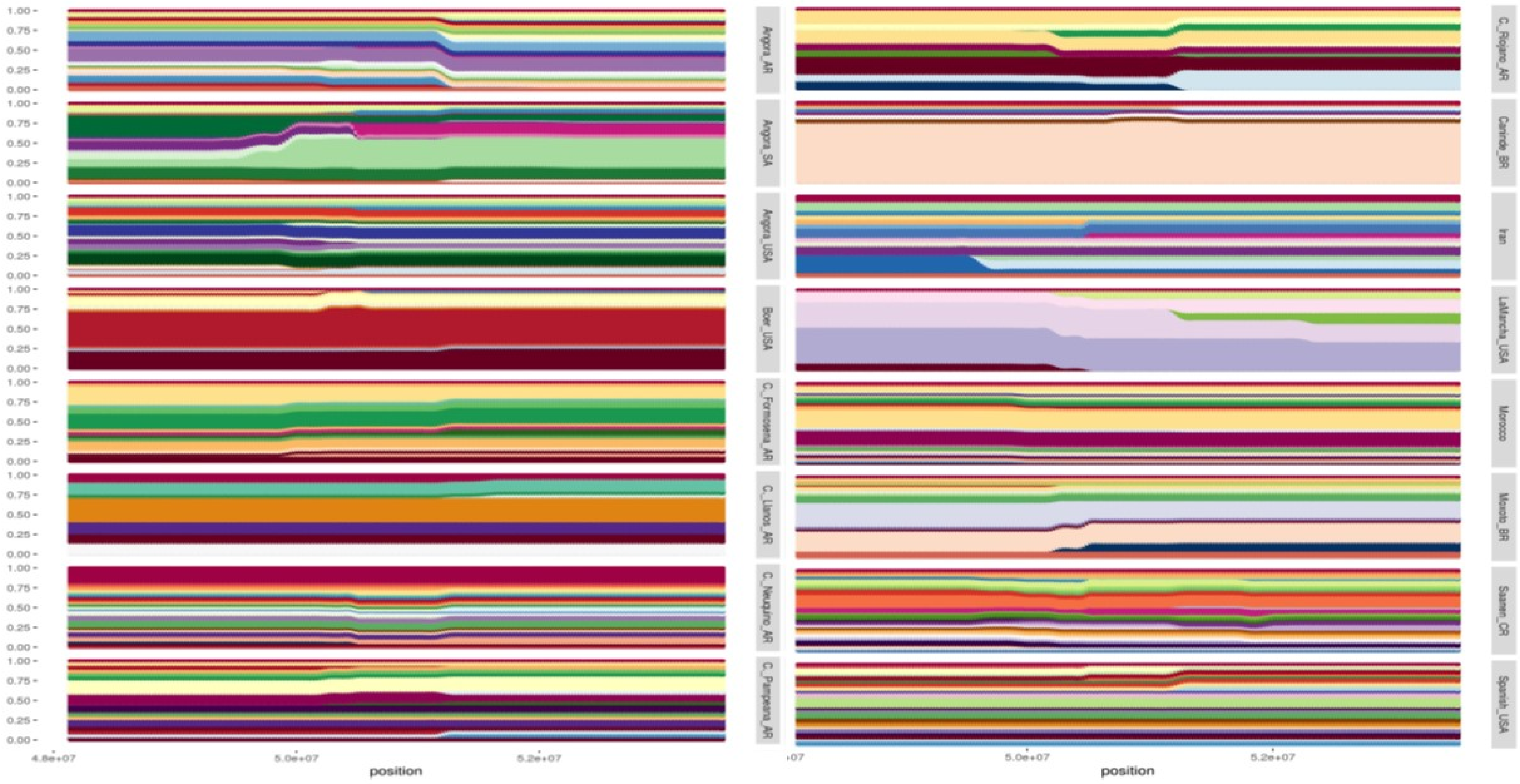


**Supplementary Figure S25**. Haplotype clusters frequencies in detected region in chromosome 7 for each of the 16 goat populations used in the test.

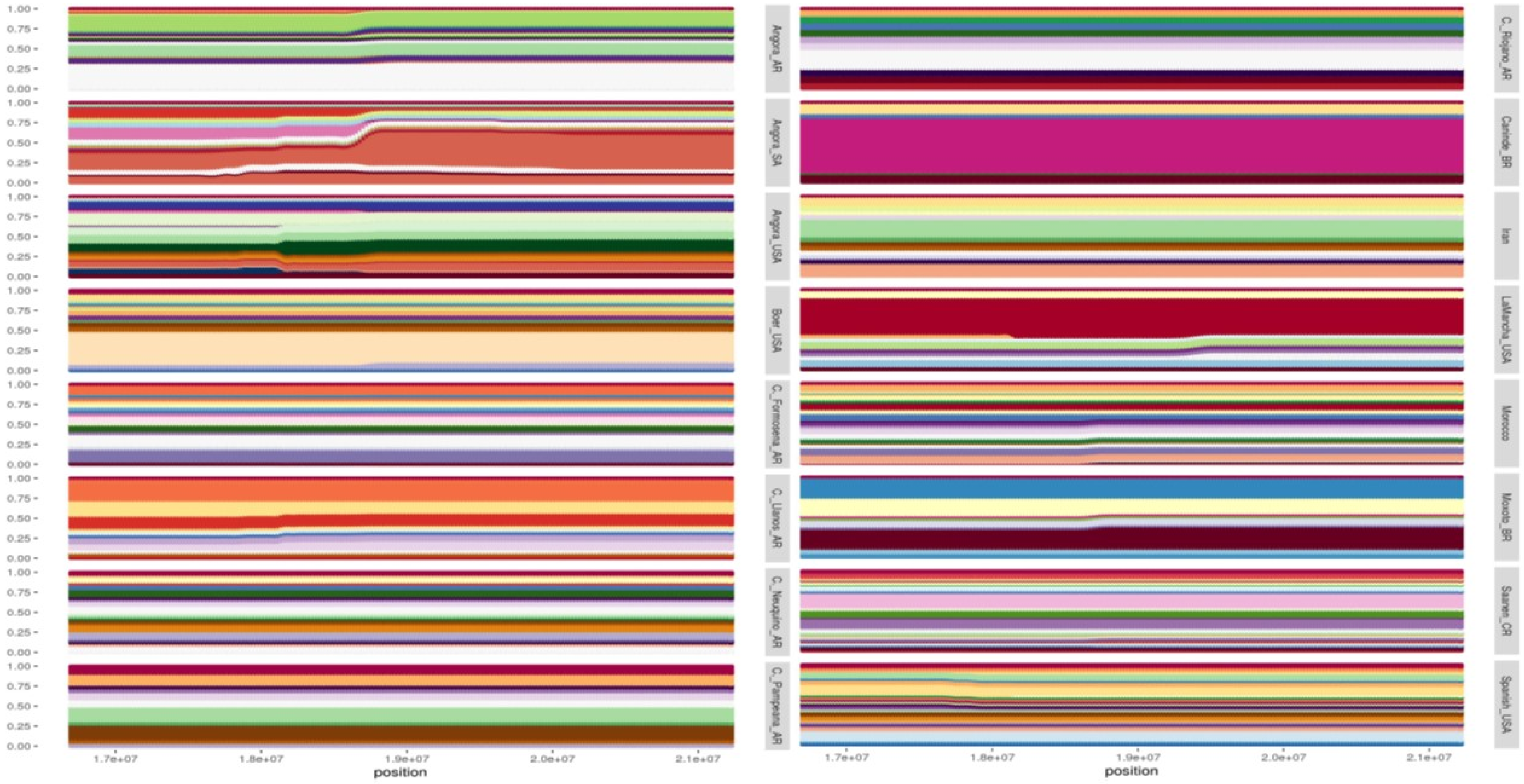


**Supplementary Figure S26**. Haplotype clusters frequencies in detected region in chromosome 10 for each of the 16 goat populations used in the test.

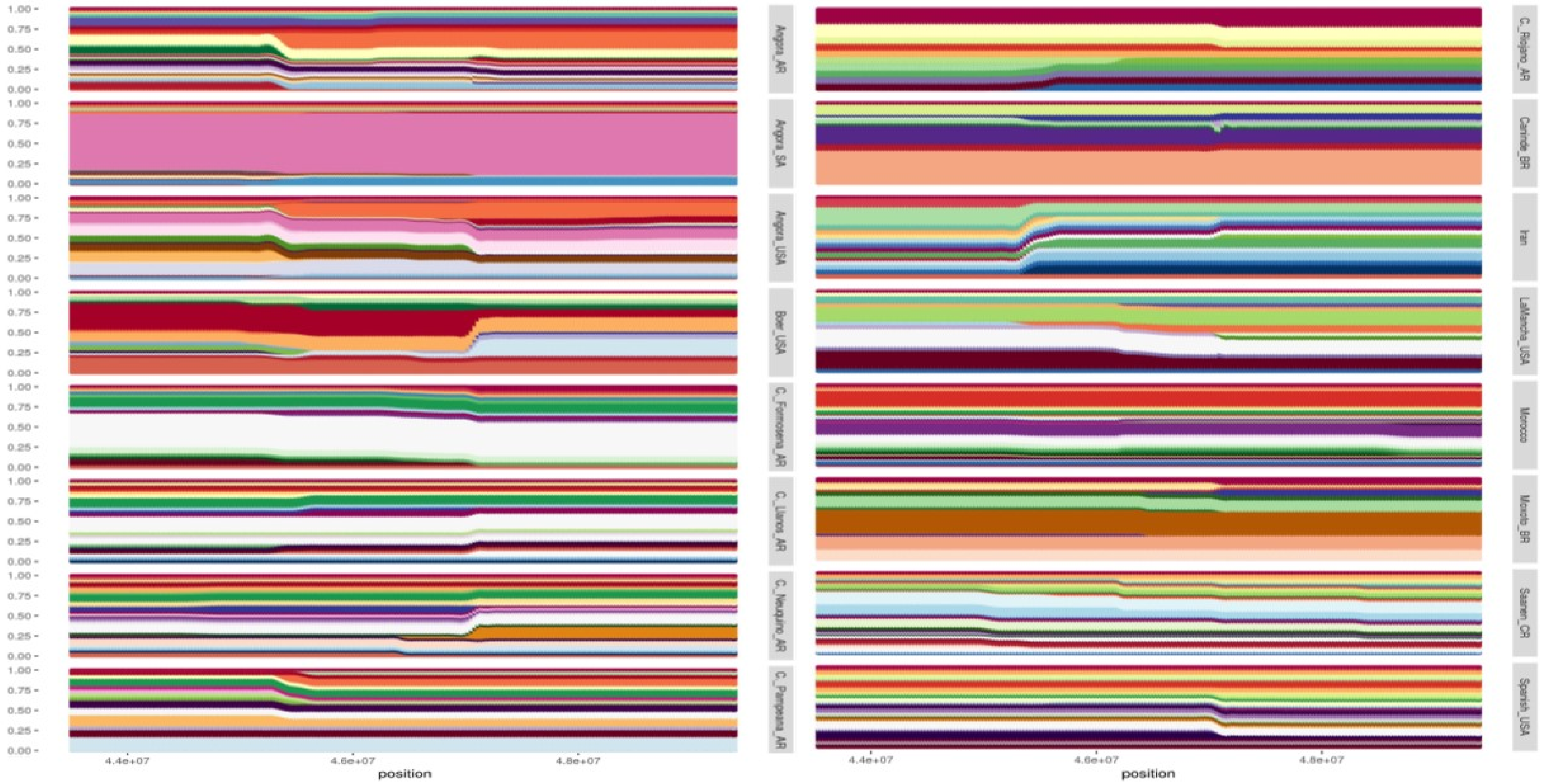


**Supplementary Figure S27.** Haplotype clusters frequencies in detected region in chromosome 23 for each of the 16 goat populations used in the test.

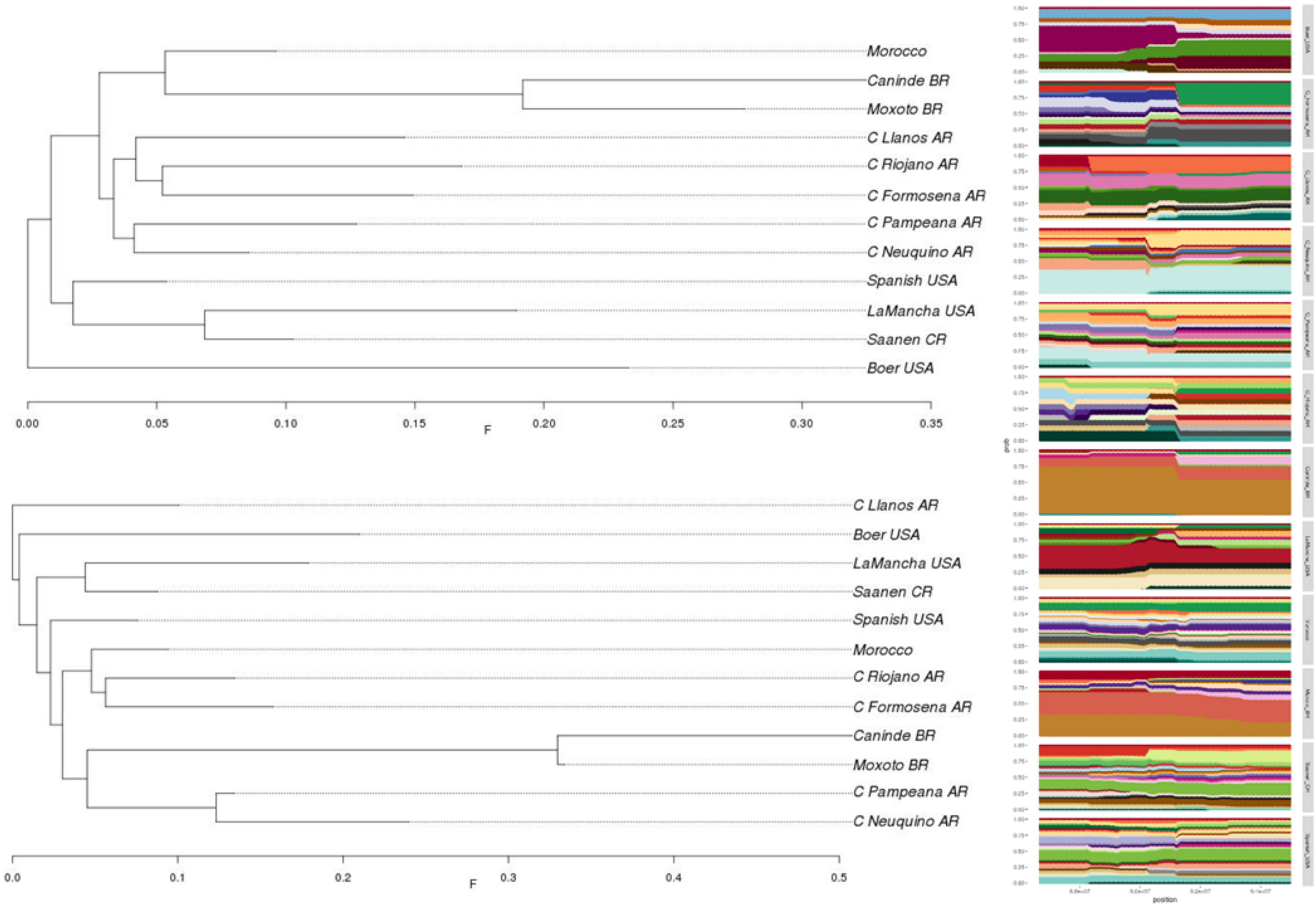


**Supplementary Figure S28.** Population trees (at left) generated using all available SNPs (top) and only 176 SNPs (bottom) surrounding the hapFLK peak in chromosome 6 analyzing 12 populations. Haplotype clusters frequencies (at right) in the region of chromosome 6 for each population used in the test.

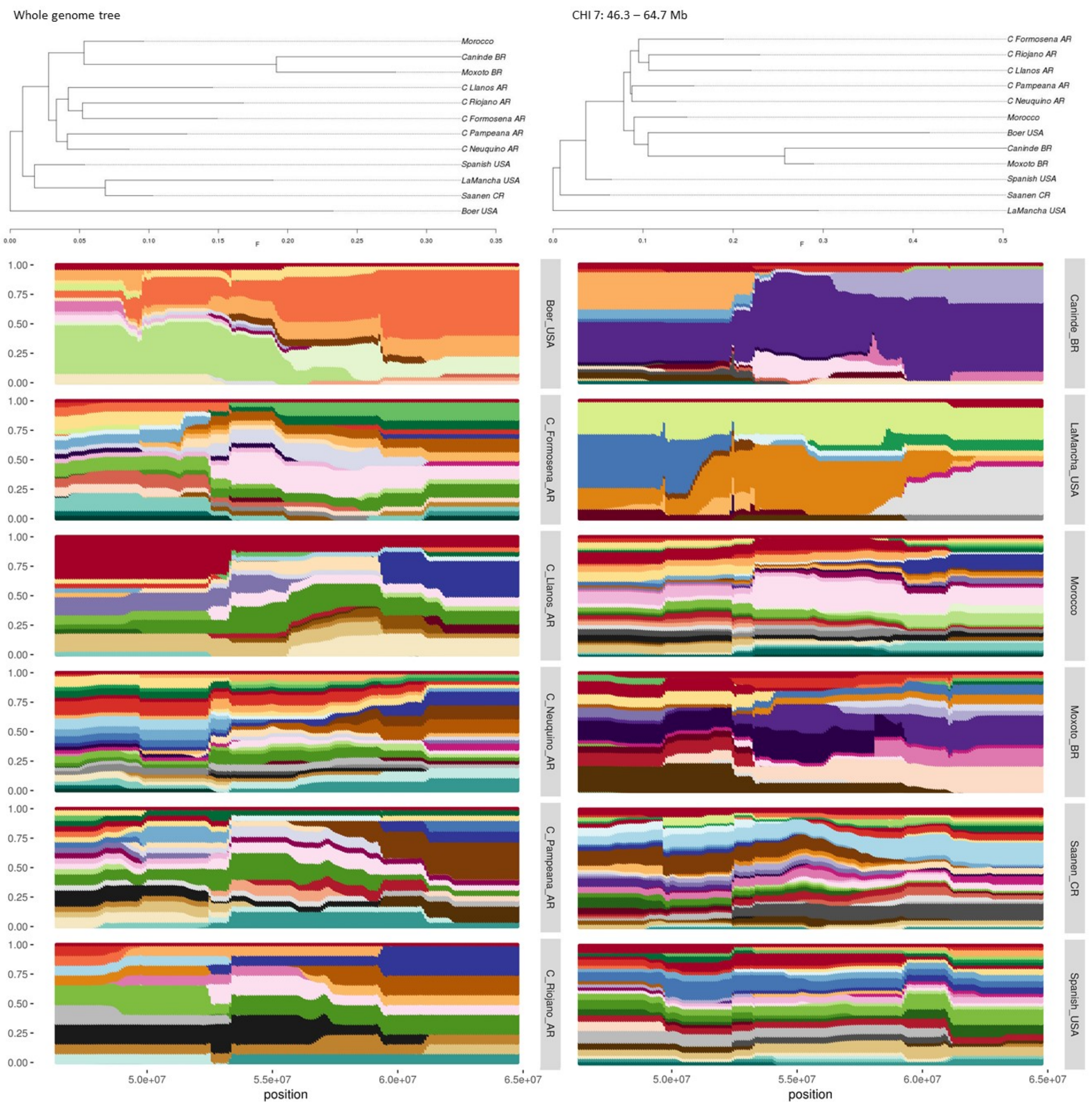


**Supplementary Figure S29.** Population trees (at top) generated using all available SNPs (left) and only 385 SNPs (right) surrounding the hapFLK peak in chromosome 7 analyzing 12 populations. Haplotype clusters frequencies (at bottom) in the region of chromosome 7 for each population used in the test.

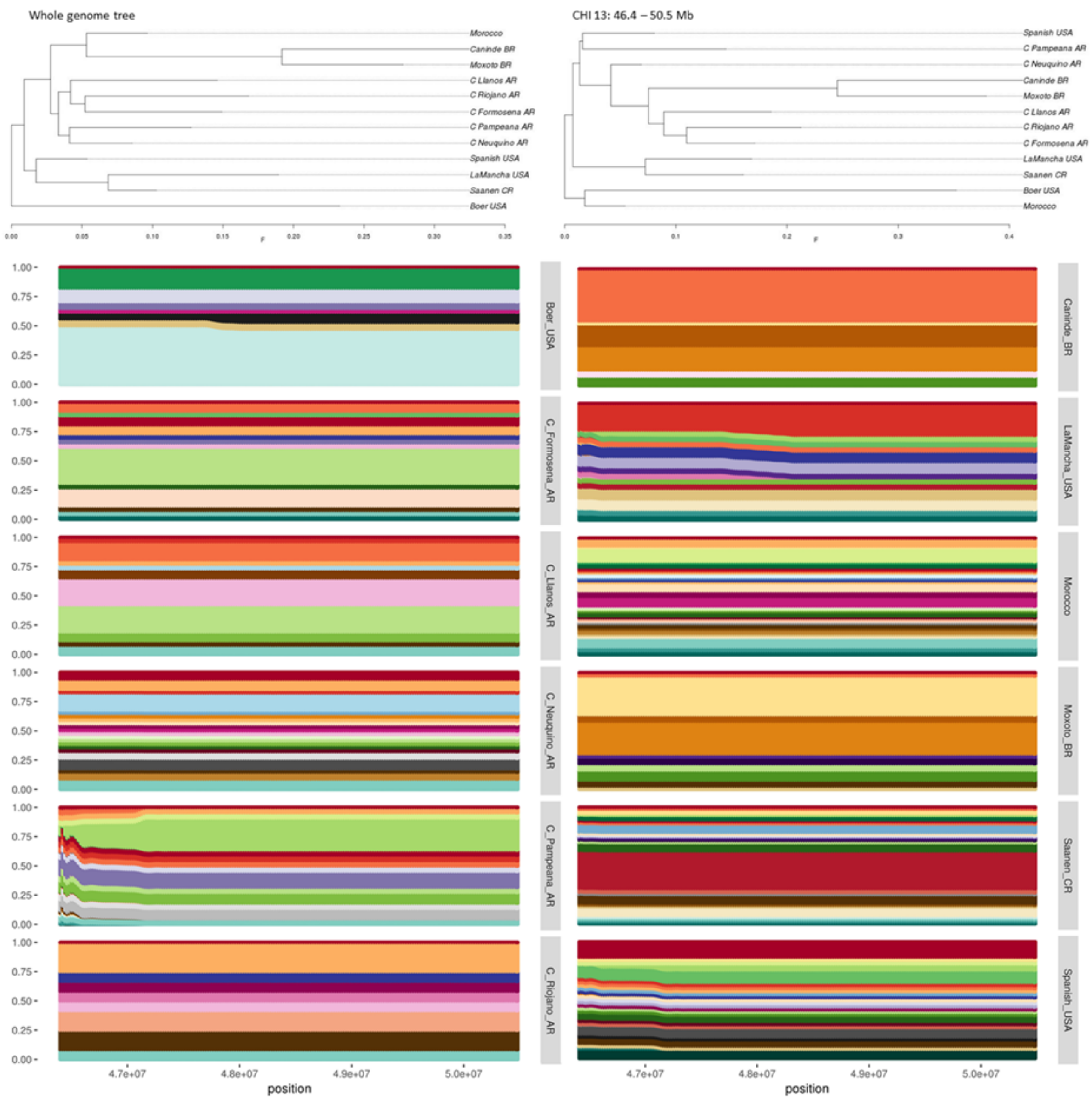


**Supplementary Figure S30.** Population trees (at top) generated using all available SNPs (left) and only 77 SNPs (right) surrounding the hapFLK peak in chromosome 13 analyzing 12 populations. Haplotype clusters frequencies (at bottom) in the region of chromosome 13 for each population used in the test.

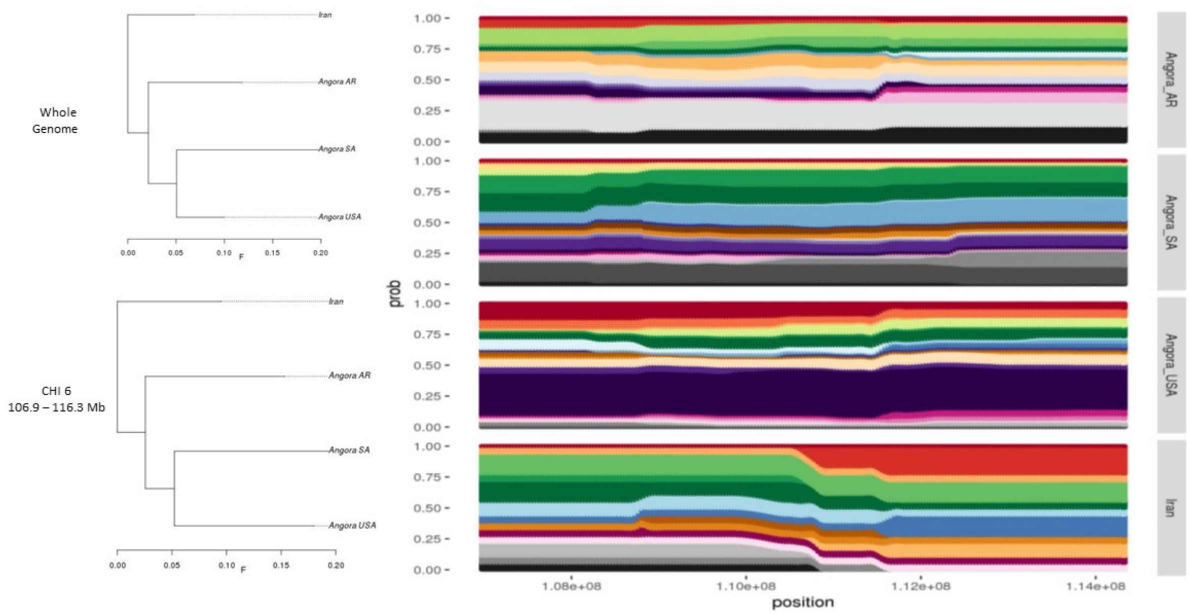


**Supplementary Figure S31.** Population trees (at left) generated using all available SNPs and only 140 SNPs surrounding the hapFLK peak in chromosome 6 analyzing only the Angora and Iran populations. Haplotype clusters frequencies (at right) in the region of chromosome 6 for each population used in the test.

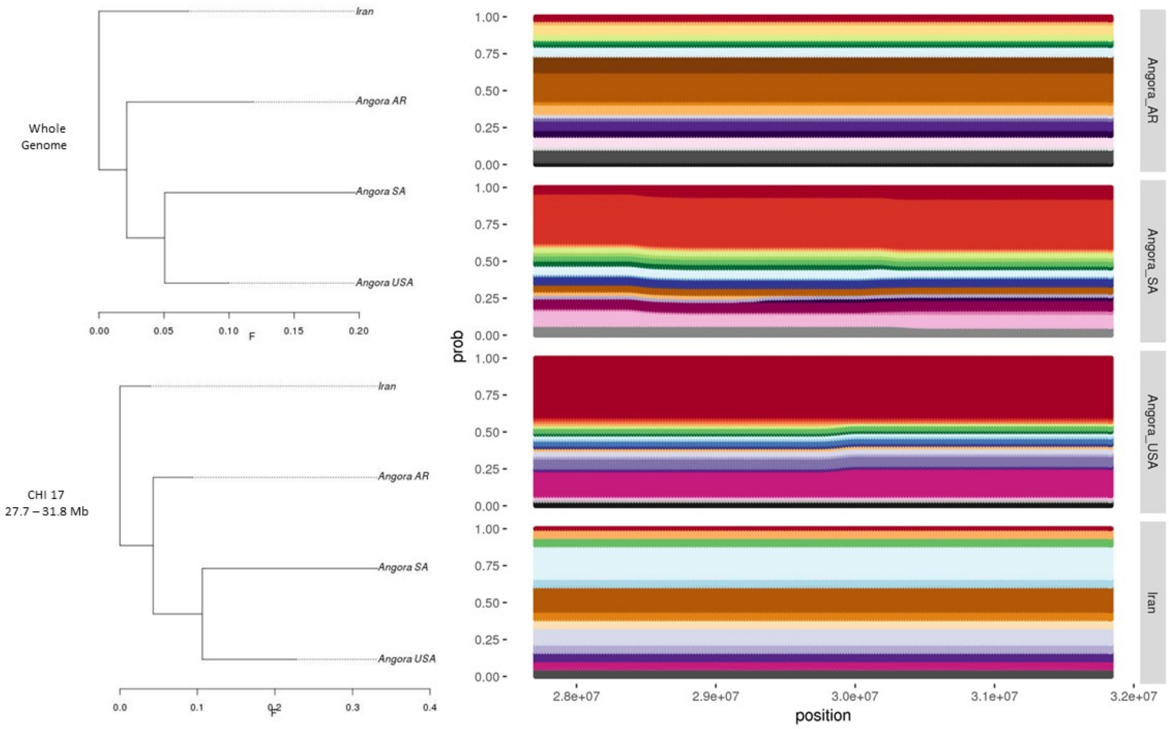


**Supplementary Figure S32.** Population trees (at left) generated using all available SNPs and only 82 SNPs surrounding the hapFLK peak in chromosome 17 analyzing only the Angora and Iran populations. Haplotype clusters frequencies (at right) in the region of chromosome 17 for each population used in the test.

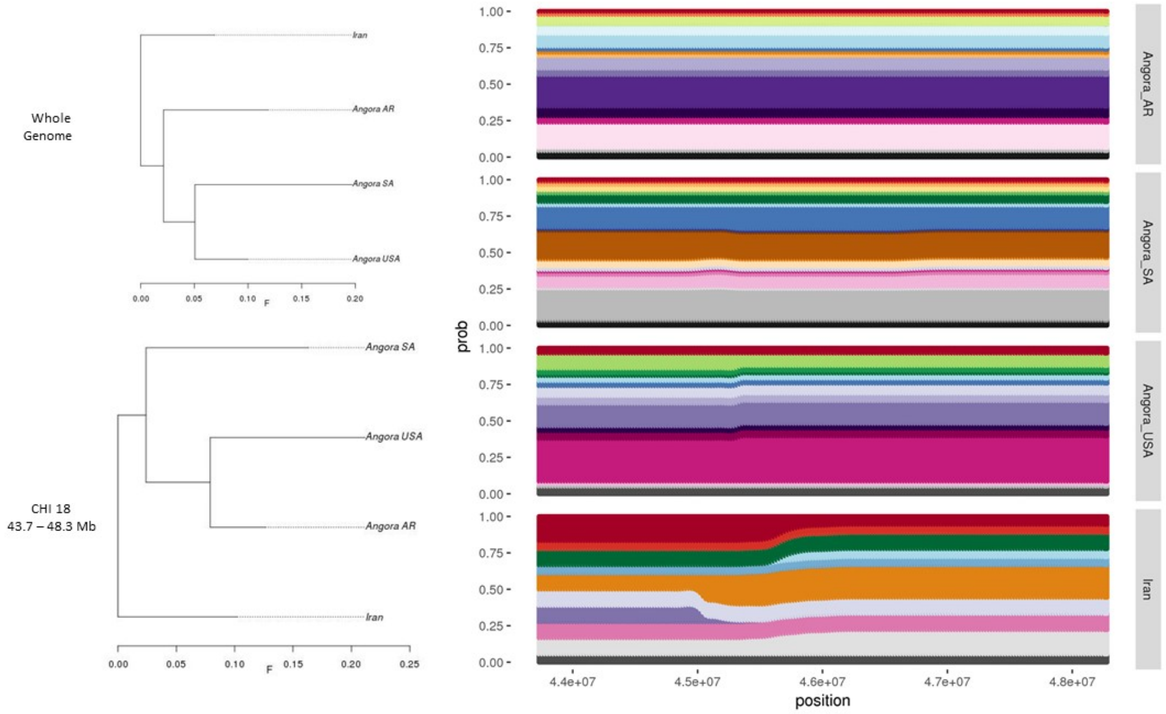


**Supplementary Figure S33.** Population trees (at left) generated using all available SNPs and only 91 SNPs surrounding the hapFLK peak in chromosome 18 analyzing only the Angora and Iran populations. Haplotype clusters frequencies (at right) in the region of chromosome 18 for each population used in the test.

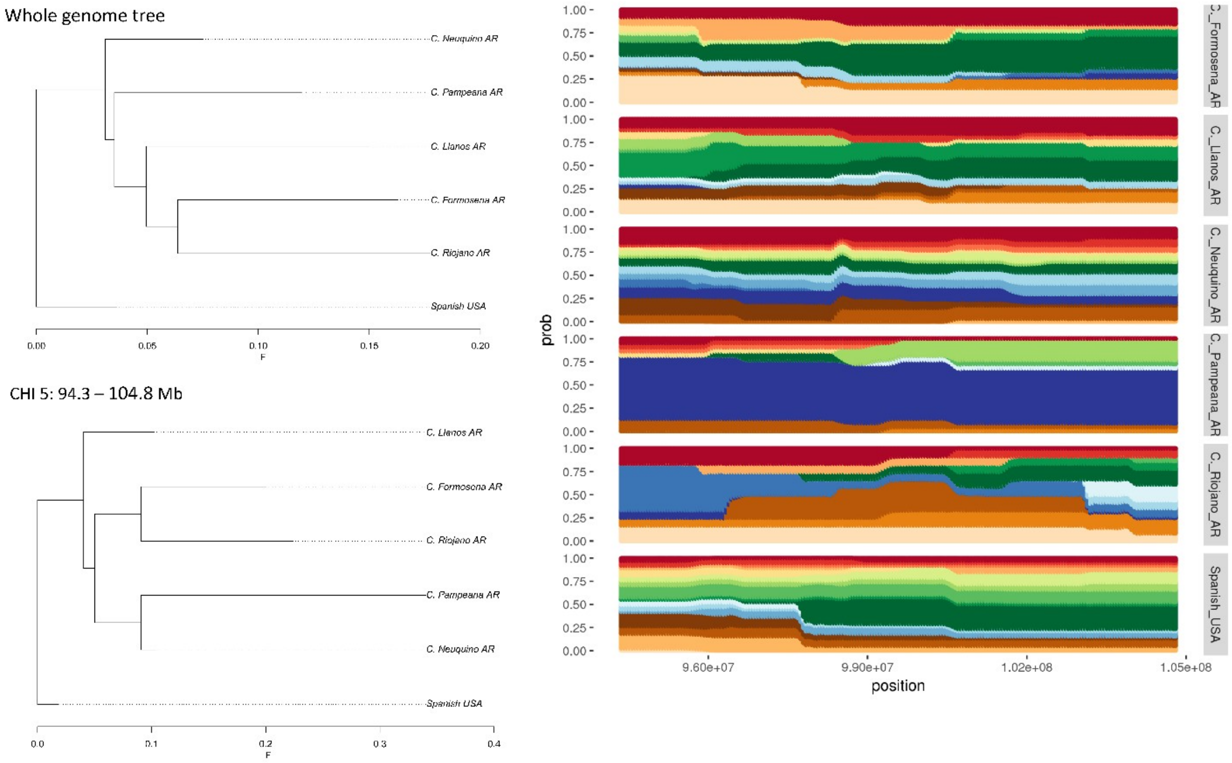


**Supplementary Figure S34.** Population trees (at left) generated using all available SNPs and only 204 SNPs surrounding the hapFLK peak in chromosome 5 analyzing only the Argentinean and Spanish populations. Haplotype clusters frequencies (at right) in the region of chromosome 5 for each population used in the test.

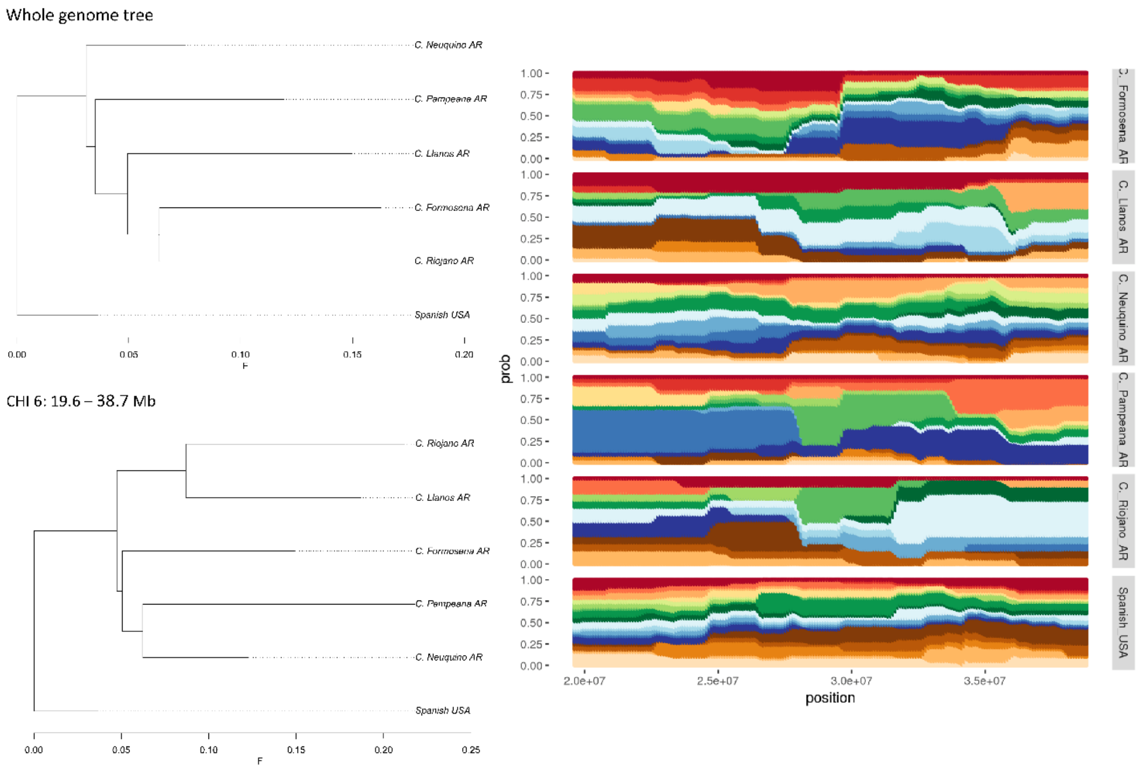


**Supplementary Figure S35.** Population trees (at left) generated using all available SNPs and only 381 SNPs surrounding the hapFLK peak in chromosome 6 analyzing only the Argentinean and Spanish populations. Haplotype clusters frequencies (at right) in the region of chromosome 6 for each population used in the test.

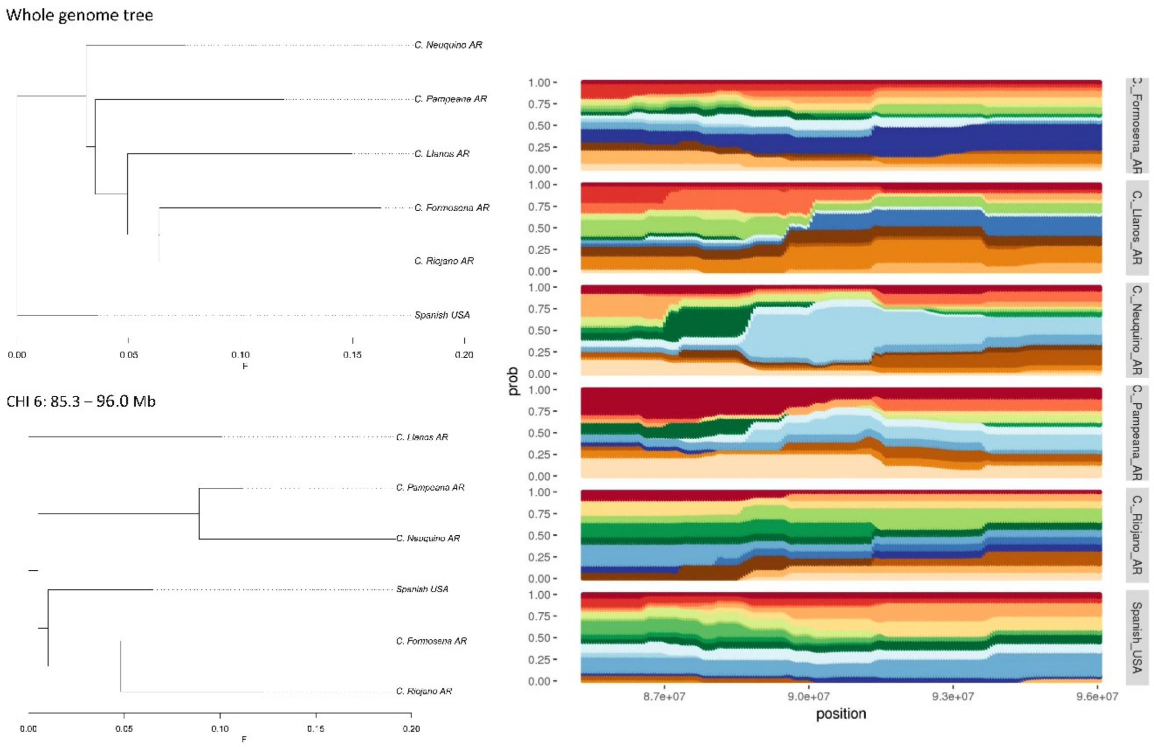


**Supplementary Figure S36.** Population trees (at left) generated using all available SNPs and only 225 SNPs surrounding the hapFLK peak in chromosome 6 analyzing only the Argentinean populations and Spanish breed. Haplotype clusters frequencies (at right) in the region of chromosome 6 for each population used in the test.

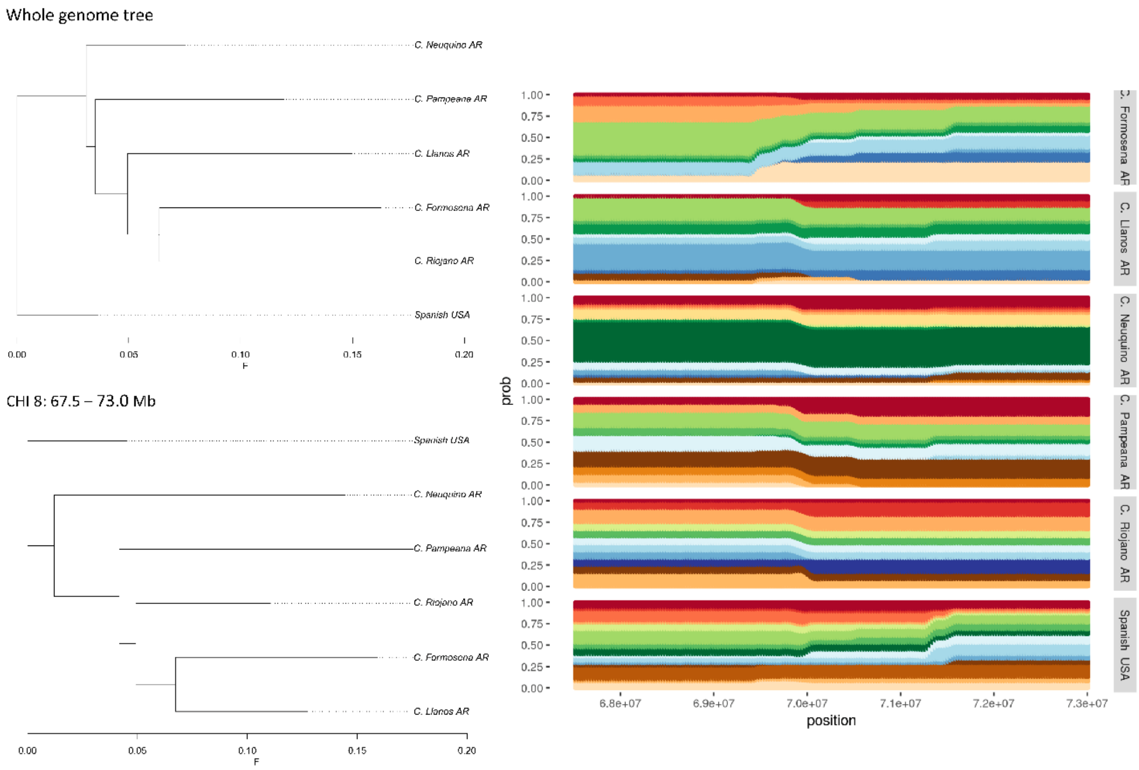


**Supplementary Figure S37.** Population trees (at left) generated using all available SNPs and only 113 SNPs surrounding the hapFLK peak in chromosome 8 analyzing only the Argentinean and Spanish populations. Haplotype clusters frequencies (at right) in the region of chromosome 8 for each population used in the test.

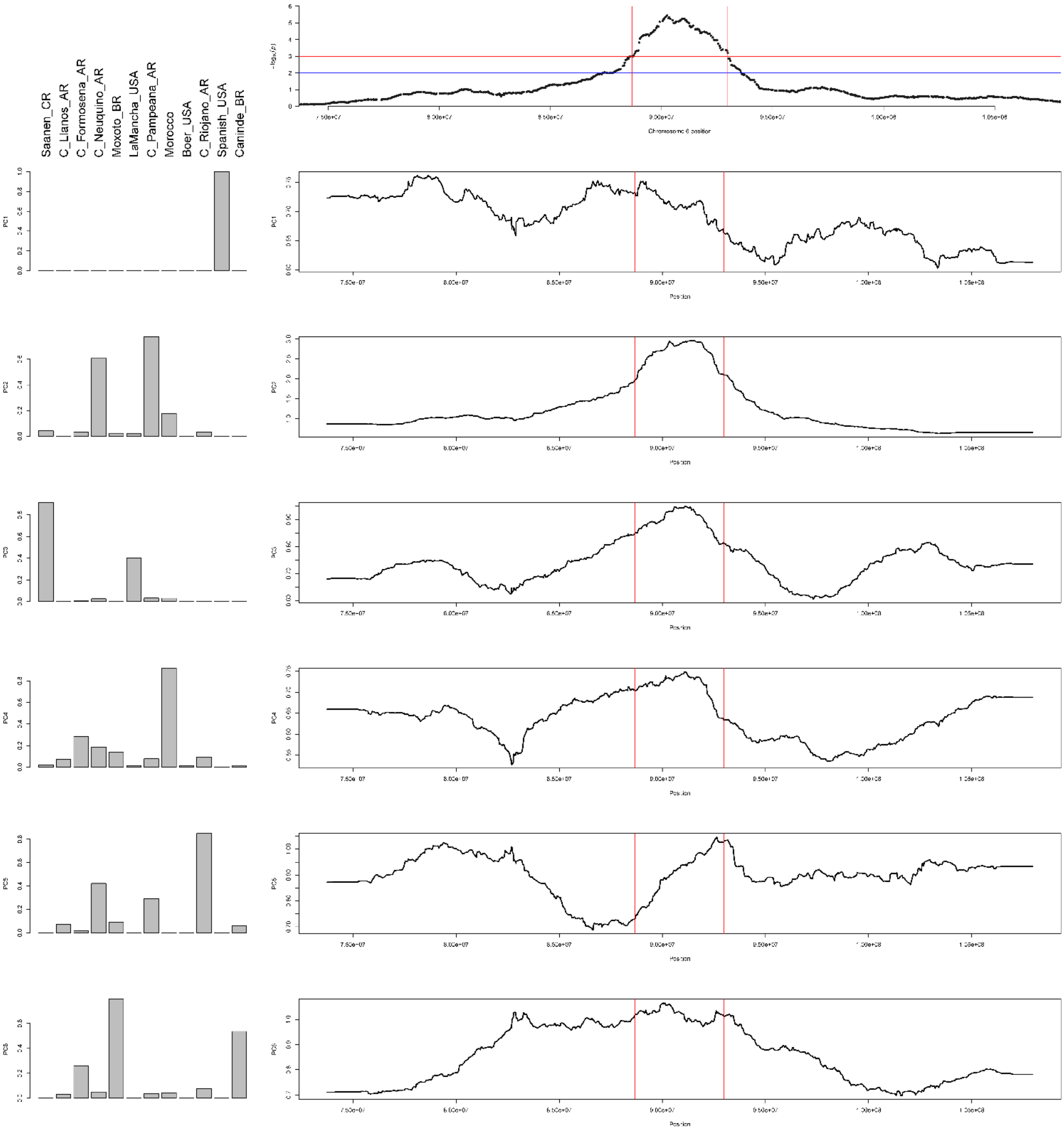


**Supplementary Figure S38**. Principal components decomposition of hapFLK for detected region on chromosome 6 using 12 populations. First line: hapFLK p-values. Each of the following lines corresponds to an orthogonal component of the population kinship matrix. For each component, populations eigenvectors are shown on the left and the projection of the test loadings plotted on the right. Red vertical lines indicate the limits of the candidate region. This plot shows, as example, the other method used for all the selected regions to identify which population was selected on each region and to identify the causal variants in each region. PC2, PC3 and PC6 had the peak pattern (at right) and shows C. Neuquino_AR and C. Pampeana_AR, Saanen_CR and LaMancha_US and Brazilian breeds as possible selected populations respectively.

## References


1. Burren, A. *et al.* Genetic diversity analyses reveal first insights into breed-specific selection signatures within Swiss goat breeds. *Anim. Genet.* **47,** 727–739 (2016).

2. Brito, L. F. *et al.* Genetic diversity and signatures of selection in various goat breeds revealed by genome-wide SNP markers. *BMC Genomics* **18,** 229 (2017).

3. Kim, E. S. *et al.* Multiple genomic signatures of selection in goats and sheep indigenous to a hot arid environment. *Heredity (Edinb).* **116,** 255–264 (2016).

4. Martin, P. *et al.* A genome scan for milk production traits in dairy goats reveals two new mutations in Dgat1 reducing milk fat content. *Sci. Rep.* **7,** 1–13 (2017).

5. Alberto, F. J. *et al.* Convergent genomic signatures of domestication in sheep and goats. *Nat. Commun.* **9,** 813 (2018).

6. Abo-Ismail, M. K. *et al.* Single nucleotide polymorphisms for feed efficiency and performance in crossbred beef cattle. *BMC Genet.* **15,** 14 (2014).

7. Song, Y. *et al.* Genome-wide association study reveals the PLAG1 gene for Knuckle, Biceps and Shank weight in Simmental beef cattle. *PLoS One* **11,** 1–10 (2016).

8. Zhang, Z. *et al.* Genome-Wide Association Study Reveals Constant and Specific Loci for Hematological Traits at Three Time Stages in a White Duroc × Erhualian F2 Resource Population. *PLoS One* **8,** (2013).

9. Bertelsen, H. P. *et al.* Detection of genetic variation affecting milk coagulation properties in Danish Holstein dairy cattle by analyses of pooled whole-genome sequences from phenotypically extreme samples (pool-seq). *J. Anim. Sci.* **94,** 1365–1376 (2016).

10. Chalkias, H. *et al.* Identification of novel candidate genes for the inverted teat defect in sows using a genome-wide marker panel. *J. Appl. Genet.* **58,** 249–259 (2017).

11. Wu, X. *et al.* Genome wide association studies for body conformation traits in the Chinese Holstein cattle population. *BMC Genomics* **14,** 897 (2013).

12. Verardo, L. L. *et al.* Revealing new candidate genes for reproductive traits in pigs: combining Bayesian GWAS and functional pathways. *Genet. Sel. Evol.* **48,** 9 (2016).

13. Chung, H. Y. & Davis, M. E. Effects of calpain genotypes on meat tenderness and carcass traits of Angus bulls. *Mol. Biol. Rep.* **38,** 4575–4581 (2011).

14. Fowler, K. E. *et al.* Genome wide analysis reveals single nucleotide polymorphisms associated with fatness and putative novel copy number variants in three pig breeds. *BMC Genomics* **14,** 784–798 (2013).

15. Zhang, L. C. *et al.* A genome-wide association study of limb bone length using a Large White x Minzhu intercross population. *Genet Sel Evol* **46,** 56 (2014).

16. Marques, E., Nkrumah, J. D., Sherman, E. L. & Moore, S. S. Polymorphisms in positional candidate genes on BTA14 and BTA26 affect carcass quality in beef cattle. *J. Anim. Sci.* **87,** 2475–2484 (2009).

17. Jung, E. J. *et al.* Genome-wide association study identifies quantitative trait loci affecting hematological traits in an F2 intercross between Landrace and Korean native

pigs. *Anim. Genet.* **45,** 534–541 (2014).

18. Buitenhuis, B. *et al.* Genome-wide association and biological pathway analysis for milk-fat composition in Danish Holstein and Danish Jersey cattle. *BMC Genomics* **15,** 1112 (2014).

19. Wang, Z. *et al.* Genome-wide association study for wool production traits in a Chinese merino sheep population. *PLoS One* **9,** 3–10 (2014).

20. Do, D. N. *et al.* Genome-Wide Association Study Reveals Genetic Architecture of Eating Behavior in Pigs and Its Implications for Humans Obesity by Comparative Mapping. *PLoS One* **8,** e71509 (2013).

21. Fan, B. *et al.* Large-scale association study for structural soundness and leg locomotion traits in the pig. *Genet. Sel. Evol.* **41,** 14 (2009).

22. Sasago, N., Abe, T., Sakuma, H., Kojima, T. & Uemoto, Y. Genome-wide association study for carcass traits, fatty acid composition, chemical composition, sugar, and the effects of related candidate genes in Japanese Black cattle. *Anim. Sci. J.* **88,** 33–44 (2017).

23. Liu, X. *et al.* Novel single nucleotide polymorphisms of the bovine methyltransferase 3b gene and their association with meat quality traits in beef cattle. *Genet. Mol. Res.* **11,** 2569–2577 (2012).

24. Liu, X. *et al.* Polymorphisms in epigenetic and meat quality related genes in fourteen cattle breeds and association with beef quality and carcass traits. *Asian-Australasian J. Anim. Sci.* **28,** 467–475 (2015).

25. Peng, W.-F. *et al.* A genome-wide association study reveals candidate genes for the supernumerary nipple phenotype in sheep ( *Ovis aries* ). *Anim. Genet.* 570–579 (2017). doi:10.1111/age.12575
